# In situ structure of the mouse sperm central apparatus reveals mechanistic insights into asthenozoospermia

**DOI:** 10.1038/s41422-025-01135-2

**Published:** 2025-06-05

**Authors:** Yun Zhu, Tingting Lin, Guoliang Yin, Linhua Tai, Lianwan Chen, Jing Ma, Guoning Huang, Yi Lu, Zhiyong Zhang, Binbin Wang, Suren Chen, Fei Sun

**Affiliations:** 1 National Laboratory of Biomacromolecules, Institute of Biophysics, Chinese Academy of Sciences, Beijing, China; 2 Chongqing Key Laboratory of Human Embryo Engineering and Precision Medicine, Center for Reproductive Medicine, Women and Children’s Hospital of Chongqing Medical University, Chongqing, China; 3 Chongqing Clinical Research Center for Reproductive Medicine, Chongqing Health Center for Women and Children, Chongqing, China; 4 School of Life Sciences, University of Chinese Academy of Sciences, Beijing, China; 5 Department of Physics, University of Science and Technology of China, Hefei, Anhui China; 6 MOE Key Laboratory for Cellular Dynamics, University of Science and Technology of China, Hefei, Anhui China; 7 Graduate School, Chinese Academy of Medical Sciences and Peking Union Medical College, Beijing, China; 8 Department of Urology, Peking Union Medical College Hospital, Beijing, China; 9 Center for Genetics, National Research Institute of Family Planning, Beijing, China; 10 Education Key Laboratory of Cell Proliferation and Regulation Biology, College of Life Sciences, Beijing Normal University, Beijing, China; 11 Center for Biological Imaging, Institute of Biophysics, Chinese Academy of Sciences, Beijing, China; 12 Guangzhou Institutes of Biomedicine and Health, Chinese Academy of Sciences, Guangzhou, Guangdong China

**Keywords:** Cryoelectron tomography, Cilia

## Abstract

The central apparatus (CA) within the sperm axoneme is vital for sperm motility, yet its molecular architecture and functional mechanisms remain incompletely understood. Combining cryo-electron tomography and AlphaFold2, we resolved the in-cell structure of mouse sperm CA at a subnanometer resolution and built a near-complete atomic model. Our analysis identified 39 CA-associated proteins, including eight previously unreported components. By presenting the full-length structures of CFAP47 and HYDIN, we elucidate their molecular roles in tethering the C1 and C2 microtubules within the CA. Specifically, HYDIN forms a semicircular chain that encircles C1 and C2, with its N-terminal half driving the C1–C2 connection and its C-terminal half providing axial support in C2. CFAP47, the core structural component of the bridge, binds C1 through its N-terminal domains, interacts with HYDIN via its central CFAP47-ring, and anchors to C2 through its C-terminal region. The significantly reduced sperm motility and impaired CA structure observed in *Cfap47-*knockout mice confirmed the important role of CFAP47. Furthermore, genetic analysis of infertile Chinese men with asthenozoospermia identified previously unreported mutations in the *CFAP47*. The CA structural model elucidates the pathogenic mechanisms of these mutations, establishing a direct link between CFAP47 dysfunction and impaired sperm motility. Therefore, our study provides mechanistic insights into CA-related fertility disorders.

## Introduction

Cilia play crucial roles in various life processes, such as embryonic development, fertility, airway function, and cerebrospinal fluid circulation.^1^ Defects in ciliary structure and function lead to ciliopathies, which include conditions such as congenital heart defects, hydrocephalus, and primary ciliary dyskinesia (PCD).^2,3^ The axoneme, a cytoskeletal scaffold of cilia, typically exhibits a “9 + 2” microtubule arrangement in motile cilia, comprising 9 peripheral doublet microtubules (DMTs), inner and outer dynein arms (IDAs and ODAs), radial spokes (RSs), and nexin-dynein regulatory complexes (N-DRCs), all surrounding a central pair of microtubules named the central apparatus (CA).^4,5^ The CA primarily governs ciliary motility, functioning as a mechanical force distributor that interfaces with RSs to transmit signals throughout the axoneme.^6,7^

Cilia from diverse organisms have been studied to understand the CA.^8,9^ However, since the CA has a dynamic nature and numerous components, the research has been limited to an exploration of the overall morphology and constituents of the CA.^10^ Recent advances based on the use of cryo-electron microscopy (cryo-EM) on purified samples from *Chlamydomonas reinhardtii* have elucidated the high-resolution structures of the CA, revealing six projections (C1a–f) on its C1 microtubule and five (C2a–e) on its C2 microtubule, offering foundational insights into the CA structure.^11,12^ However, separation and purification processes disrupt the structure of the CA, obscuring critical structural details, especially in the bridge region connecting C1 and C2. A more comprehensive understanding of CA assembly is still needed, especially in higher-order animals, where significant differences in CA components are observed compared with those in lower-order species.^13^

The resolutions of the in situ structural studies of CA reported thus far are all below 26 Å.^14,15^ By utilizing visual proteomics technology^16^ and combining cryo-electron tomography (cryo-ET) with AlphaFold2 modeling, we determined the near-complete native CA structure in mouse sperm. Subtomogram averaging (STA) enabled CA reconstruction at a resolution of up to 5.5 Å, providing well-resolved tertiary structures to build models of 39 CA-associated proteins. Among these, eight proteins were newly identified as CA components, including GRK3, ANKMY1, LRRC43, GOT1L1, LRRD1, MAP1S, GMCL1 (BTBD16), and SPACA9. Notably, we resolved the full-length structures of CFAP47 and HYDIN, providing new insights into their architecture and interactions with various CA components. In the sperm of *Cfap47*-knockout (KO) mice, a hollow bridge region within the CA structure was observed, correlating with abnormal sperm motility and confirming the essential role of CFAP47 in CA function. Furthermore, genetic analysis of 320 Chinese men with asthenozoospermia identified previously unreported mutations in the *CFAP47* gene in two patients. Our structural model elucidates the pathogenic mechanisms of these mutations, establishing a direct link between *CFAP47* dysfunction and impaired sperm motility. Therefore, our study elucidates the molecular mechanisms underlying the role of CA components in ciliary motility, as represented by CFAP47, and provides a framework for understanding sperm axoneme-related fertility disorders through a visual proteomics approach.

## Results

### In-cell structural determination

**Fig. 1:**
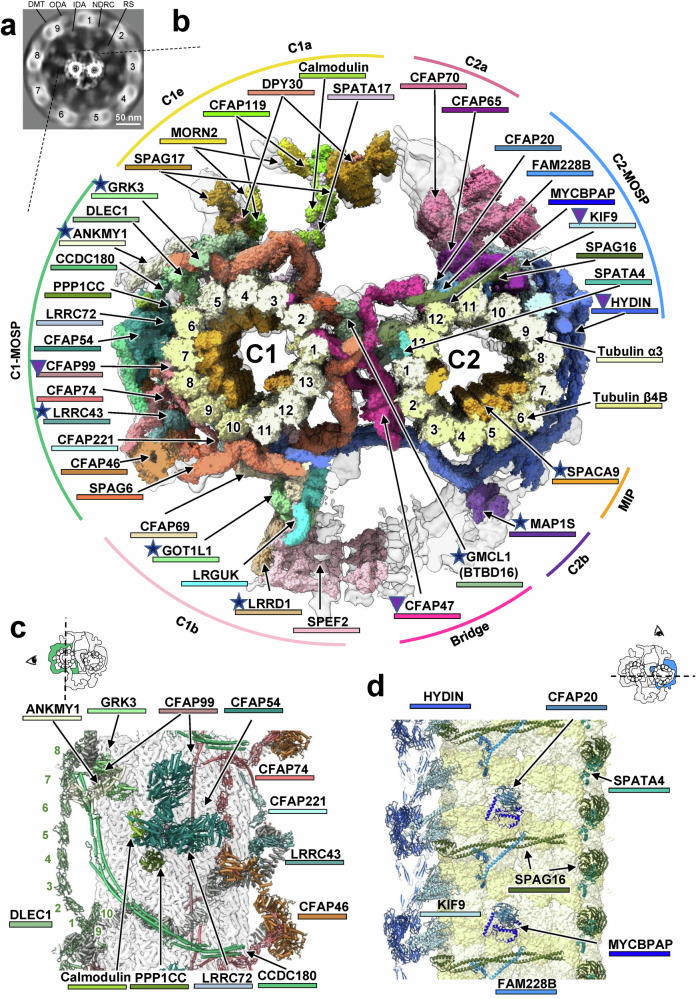
Overall structure of the mouse sperm CA. **a** Slice view of the entire mouse sperm axoneme structure, highlighting the major components: DMT, RS, ODA, IDA, and N-DRC. The numbers indicate the components in different directions. **b** Molecular composition of the CA architecture. The protein components are colored differently (Supplementary information, Table S10) and categorized based on their specific locations within the CA, including MOSPs in C1 and C2, five large projections (C1a/C1e, C1b, C2a, and C2b), the bridge linking C1 and C2, and MIP. The pentagram symbols indicate proteins newly identified in mouse sperm CA, while triangle symbols represent components with more complete structural models. **c** Depiction of the overall protein composition of C1-MOSP in the 32-nm repeat. The numbers indicate the order of the 10 ASH domains of DLEC1. **d** Depiction of the overall protein composition of C2-MOSP in the 16-nm repeat.

In prior research, we successfully determined the native structure of mouse sperm DMT at a 4.5–7.5 Å resolution and built a model with 36 non-tubulin components.^17^ Given the lower particle number of CAs compared to DMTs in tomograms, we collected extensive cryo-ET data on the sperm axoneme. Mouse sperms were vitrified on EM grids, and 200 nm lamellae were prepared via cryo-FIB milling, with the structural features and orientations of CA particles discernible in the tomograms (Supplementary information, Fig. S1). By employing solely data-driven templates, we identified a 32-nm repeating CA unit through 3D classification and refinement of subtomograms (Supplementary information, Fig. S2). Enlarging the box size of the CA map revealed distinct densities of the 9 DMTs (designated DMT1 to DMT9) in the axoneme, confirming structural integrity (Fig. 1a). Local refinements yielded resolutions of 7.7 Å for C1 microtubule and 6.6 Å for C2 microtubule, with various components achieving resolutions ranging from 5.5 Å to 18 Å (Supplementary information, Fig. S3 and Table S1). By combining local maps, we generated a composite reconstruction of the native CA structure (Supplementary information, Fig. S2).

This map was compared to the reported cryo-EM maps of CA across species (Supplementary information, Figs. S4, S5). The map of CA isolated from *C. reinhardtii* revealed significant density loss between C1 and C2 microtubules and in multiple projections,^11,12^ whereas our map maintained these densities (Supplementary information, Fig. S4a). There were also noticeable shifts in projection positions in their maps compared to ours (Supplementary information, Fig. S4), which may due to several factors, including the loss of CA components or projections, the absence of RS, and species-specific differences. Additionally, we observed the disappearance of the C1f projection in mouse sperm, which is consistent with the reported CA structure in pigs and horses^15^ (Supplementary information, Fig. S4a). Our higher resolution CA map aligns with the previously reported in situ CA structures across various species (Supplementary information, Fig. S5), demonstrating both the reliability of our structural analysis and the conservation of the CA architecture across species. Notably, the structures of human and mouse sperm CA were remarkably similar, differing primarily in the absence of certain microtubule inner proteins (MIPs) within the human C2 lumen (Supplementary information, Fig. S5a).

The clearly resolved secondary and tertiary structures in most regions of our CA map enabled us to interpret the maps and build pseudoatomic models (Supplementary information, Figs. S6–S19). This was accomplished by incorporating various visual proteomics methods,^16^ including the use of homologous structural data from *C. reinhardtii* cilia CA,^11,12^ mass spectrometry (MS) of mouse sperm (Supplementary information, Table S2),^18^ screening of AlphaFold2-predicted models with the DomainFit tool,^19^ and mutagenesis studies. Our final model of the 32-nm CA repeating unit in mouse sperm comprises 260 tubulin and 206 non-tubulin chains. Among these, there are 45 different polypeptide chains, including 39 known proteins and 6 unknown proteins. And among the 39 known proteins, there are eight proteins newly identified as CA components: GRK3, ANKMY1, LRRC43, GOT1L1, LRRD1, MAP1S, GMCL1 (BTBD16), and SPACA9. Additionally, we provided more complete structural models for four previously identified proteins: CFAP47, HYDIN, CFAP99, and KIF9 (Supplementary information, Fig. S19 and Table S3).

The mouse sperm CA structure is an asymmetric complex with various projections around the C1 and C2 microtubules (Fig. 1b; Supplementary information, Video S1). Each microtubule consists of 13 protofilaments with seams facing each other at their connecting bridge. In addition to microtubules, CA organization can be classified into four categories: (1) microtubule outer surface proteins (MOSPs) in C1 and C2, (2) five large projections (C1a/C1e, C1b, C2a, and C2b), (3) MIPs within the C1 and C2 lumens, and (4) the bridge linking C1 and C2 (Fig. 1b). The C1a, C1b, C2a, and C2b projections exhibit 16-nm repeats, whereas the C1e projection shows a 32-nm repeat (Supplementary information, Fig. S20a, b). Notably, C1-MOSP displays mainly 32-nm repeats, whereas C2-MOSP and the bridge predominantly feature 16-nm repeats.

### Structural assembly of CA MOSPs

The CA of mouse sperm is structurally similar to that of *C. reinhardtii* cilia^11^ for conserved components. In C1-MOSP, SPAG6 — a conserved armadillo repeat protein homologous to PF16 — forms a spring-like scaffold around the C1 microtubule at 32 nm and 16 nm intervals, which is critical for structural stability of CA (Supplementary information, Fig. S20c). Proteins containing the ASH (ASPM, SPD-2, HYDIN) domain are commonly associated with CA assembly,^20^ and notably, DLEC1 has 10 ASH domains and integrates into CA structures at 32-nm intervals (Fig. 1c). DLEC1 interacts with SPAG6 via terminal domains, forming the base of the C1e projection, parallel to the C1 protofilament-4 (Supplementary information, Fig. S20d). Mutations in its homolog, FAP81, result in the absence of the C1e projection in *C. reinhardtii* cilia.^21^ Arranged along the longitudinal axis of C1 microtubules, proteins such as CCDC180, CFAP99, and CFAP221 contribute to periodic structures, recruiting globular proteins such as ANKMY1, PPP1CC, LRRC43, GRK3, CFAP54, and CFAP46 (Fig. 1c). It is important to note that the assignment of PPP1CC remains uncertain due to its structural similarity to PPP1CA and PPP1CB (Supplementary information, Fig. S14). CFAP54 and CFAP46 differ structurally from their *C. reinhardtii* counterparts (Supplementary information, Fig. S21a, b). The N-terminal domain (NTD) of CCDC180 interacts with ANKMY1, GRK3, and CFAP99 NTD (Supplementary information, Fig. S20e); CFAP46 connects to SPAG6, LRRC43, and CFAP74 (Supplementary information, Fig. S20f); and CFAP54 encases calmodulin (CaM) and binds to LRRC72 and PPP1CC (Supplementary information, Fig. S20g). Notably, ANKMY1, GRK3, and LRRC43 are absent from the *C. reinhardtii* CA structure,^11,12^ suggesting that they are novel CA components specific to mammals or mammalian sperm. All these three CA components are positioned near RS interfaces, suggesting their potential involvement in CA–RS interactions. Additionally, GRK3, a G protein-coupled receptor kinase, was found to localize to elongating spermatids in the testis and to the midpiece of mature sperm,^22^ indicating its potential involvement in chemoreceptor responses or the regulation of motility through phosphorylation. However, it is important to note that the assignment of GRK3 remains uncertain due to its structural similarity to GRK2 (Supplementary information, Fig. S9).

Unlike C1-MOSP, C2-MOSP lacks PF16 spirals and displays 16-nm and 8-nm periodicities (Supplementary information, Fig. S20b). SPAG16 features a C-terminal β-propeller domain that binds to the seam of the C2 microtubule with a periodicity of 8 nm and a coiled-coil domain that dimerizes with adjacent copies, resulting in a transition from 8-nm to 16-nm periodicity (Fig. 1d). SPAG16 serves as the foundation for CFAP65 and the C2a projection, while the mutation of its homolog protein PF20 in *C. reinhardtii* leads to the loss of the entire CA.^23^ The β-propeller domain of SPAG16 binds with SPATA4 at the seam (Supplementary information, Fig. S21c). Both SPATA4 and FAM228B have additional C-terminal domains (CTDs) compared with their homologous proteins in *C. reinhardtii*, which enhances interactions with the C2 microtubule (Supplementary information, Fig. S21c, d). CFAP20 links the A and B tubules in DMT,^17^ but it attaches to the C2 microtubule with a minimal contact area in the CA (Fig. 1d). Like SPAG16, KIF9 has an N-terminal globular domain that binds to protofilament 9 of the C2 microtubule with a periodicity of 8 nm and a C-terminal coiled-coil domain that binds to CFAP65 in different conformations, resulting in a transition from 8-nm to 16-nm periodicity (Supplementary information, Fig. S21e). Notably, the CTD of KIF9 was not identified in prior structures of the *C. reinhardtii* CA,^11,12^ whereas our in situ structure provides more complete structural information.

### Structural assembly of large CA-projections

**Fig. 2:**
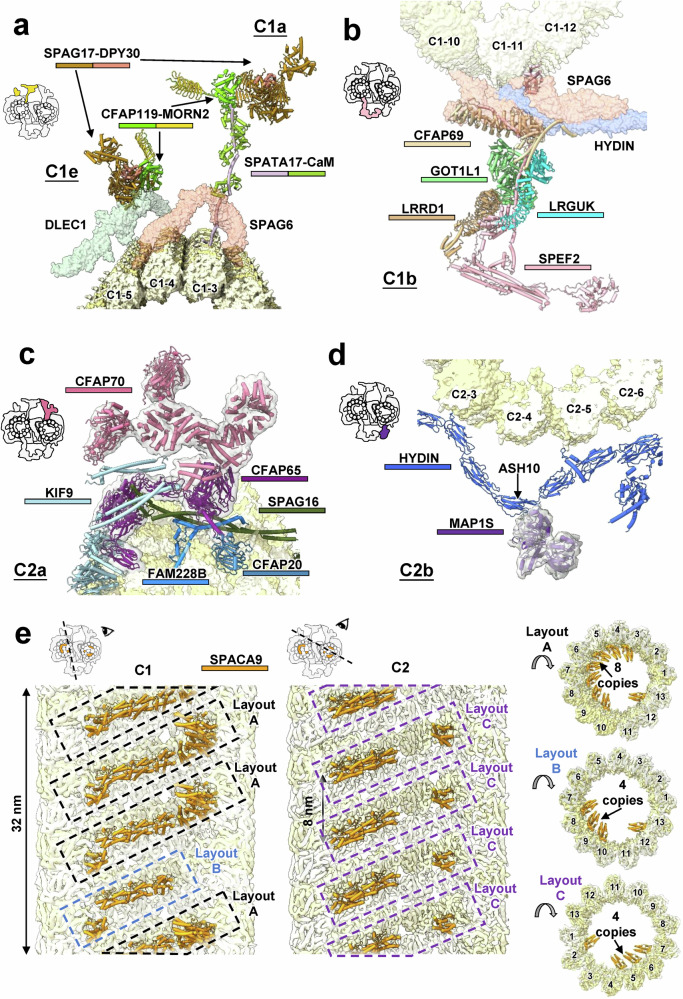
Structural composition of large projections and MIPs in the mouse sperm CA. **a** Structural composition of the C1e and C1a projections. **b** Structural composition of the C1b projection. **c** Structural composition of the C2a projection. **d** Structural composition of the C2b projection. **e** Structural composition of MIPs in the CA. SPACA9 exhibits 32-nm and 8-nm periodicities in the lumen of the C1 and C2 microtubules, respectively. SPACA9 shows three distinct patterns on the microtubule wall within the CA. Proteins are distinctly colored for clarity (Supplementary information, Table S10).

The mouse sperm CA has five large projections: C1a, C1b, C1e, C2a and C2b. Their overall structures resemble those of the *C. reinhardtii* CA.^11,12^ C1a and C1e share components such as SPAG17, MORN2, CFAP119, and DPY30. C1a’s assembly is centered on the rachis protein SPATA17, which extends from the C1 protofilament-3 and binds to SPAG6, forming stalks with three calmodulin molecules bound, whereas SPAG17–DPY30 forms their heads (Fig. 2a). Compared with its homolog PF6 in *C. reinhardtii*, SPAG17 possesses an additional globular domain and several longer helices (Supplementary information, Fig. S22a). In *Spag17*-KO mice, the C1a projection disappears in the axoneme CA.^24^ FAP114 and FAP119 in *C. reinhardtii* share homology with the mouse protein CFAP119 (Supplementary information, Fig. S22c). In addition, the algal-specific protein FAP7, the α-helical connection network between C1a and C1e, and the stalk-attached protein FAP101 in *C. reinhardtii* are absent in our map (Supplementary information, Fig. S22b), highlighting evolutionary differences.

The C1b projection is assembled around SPEF2 (CPC1 homolog), which protrudes from the C1 protofilament-11/12 and interacts with SPAG6, CFAP69, and HYDIN NTD (Fig. 2b), organizing additional components such as GOT1L1, LRGUK, and LRRD1 (Supplementary information, Fig. S22d, e). Compared with *C. reinhardtii* CA, SPEF2 and LRGUK differ structurally from their counterparts, and FAP42, HSP70A, and Enolase that are present in *C. reinhardtii* are absent in mice (Supplementary information, Fig. S22f–h). Notably, GOT1L1 and LRRD1 are absent from the *C. reinhardtii* CA structure,^11,12^ suggesting that they are novel CA components specific to mammals or mammalian sperm. Data from the Human Protein Atlas indicate that GOT1L1 and LRRD1 are highly expressed in spermatids, supporting their likely role as sperm-specific components. Recently, LRRD1 has been identified as a component of the mouse sperm axoneme RS3,^25^ suggesting that it may play different roles at various locations within the axonemal structure. Furthermore, GOT1L1 is potentially involved in D-aspartate synthesis, which is spatiotemporally regulated in mouse testes and modulates spermatogenesis.^26,27^ However, it is important to note that the assignment of GOT1L1 in the C1b projection remains uncertain due to its structural similarity to GOT1 and GOT2 (Supplementary information, Fig. S10). Further research is required to identify this component and elucidate its biological function.

We identified two major components of the C2a projections: CFAP65 and CFAP70; the latter forms a homodimer involved in CA–RS interactions (Fig. 2c). CFAP65 is situated on a structural base composed of KIF9, FAM228B, CFAP20, and SPAG16. In contrast to C2a, the structural composition of C2b has remained unclear for a long time due to its dynamic nature.^11,12^ Here, we identified HYDIN as the primary component of the C2b region (discussed later), and found that MAP1S is also located within C2b (Fig. 2d). MAP1S is known to control microtubule stability in cells,^28^ and its mutations have been identified in male patients from couples experiencing implantation failure.^29^ Data from the Human Protein Atlas indicate that MAP1S is highly expressed in spermatids, suggesting its critical role in sperm function. Our structure shows that MAP1S binds to the ASH10 domain of the HYDIN protein, forming the core structure of the C2b projection and potentially facilitating the assembly of other C2b components (Fig. 2d).

### MIP in sperm CA

In contrast to sperm DMT,^17^ the C1 and C2 microtubules in the CA show significantly lower MIP density, with SPACA9 being the most prominent (Fig. 2e). SPACA9 specifically binds at the intradimer interface between α- and β-tubulin, arranging itself in a spiral layout along the microtubule with an 8-nm longitudinal periodicity. Notably, SPACA9 was previously proposed as a CA component,^30^ and our CA structure provides direct evidence supporting this notion. Its arrangement pattern in CA differs from those in sperm DMT and in microtubule singlets.^17,30^

In the CA, we identified three spiral layouts of SPACA9, designated as layout A, B, and C. In C1, SPACA9 exhibits a repeating A-A-A-B layout, resulting in a 32-nm longitudinal periodicity. In C2, SPACA9 displays only layout C, with an 8-nm periodicity (Fig. 2e). Layout A contains eight copies of SPACA9 in each spiral layer, with seven of them located between protofilaments 3 and 10, and one between protofilaments 12 and 13. Layout B has four copies of SPACA9 in each spiral layer, with three of them located between protofilaments 7 and 10, and one between 12 and 13. Layout C also contains four copies of SPACA9 in each spiral layer, with three copies located between protofilaments 4 and 7 and one between 1 and 2 (Fig. 2e). Layouts B and C may reinforce CA binding to RS on DMT7 (denoted DMT7_RS) and DMT4_RS, respectively, whereas layout A enhances the binding to RS on DMT7/8/9 (Fig. 1a).

The MIPs in mammalian CAs differ from those in *C. reinhardtii*, where the MIPs are primarily FAP196, FAP225, and others. In the *C. reinhardtii* CA, FAP275 and FAP105 are predominant in the C1 lumen, with their binding sites different from those of SPACA9, while the position of FAP196 overlaps with that of SPACA9 in the C2 lumen, suggesting that SPACA9 replaces FAP196 in the stabilization of CA in mammals (Supplementary information, Fig. S23a, b). Additionally, our CA structures reveal some MIP densities that remain unassigned, likely due to their flexible nature and lack of clear structural features needed for protein identification (Supplementary information, Fig. S23c). These unresolved components may contribute to the establishment of the SPACA9 pattern.

### HYDIN externally connects C1, C2 and the bridge

**Fig. 3:**
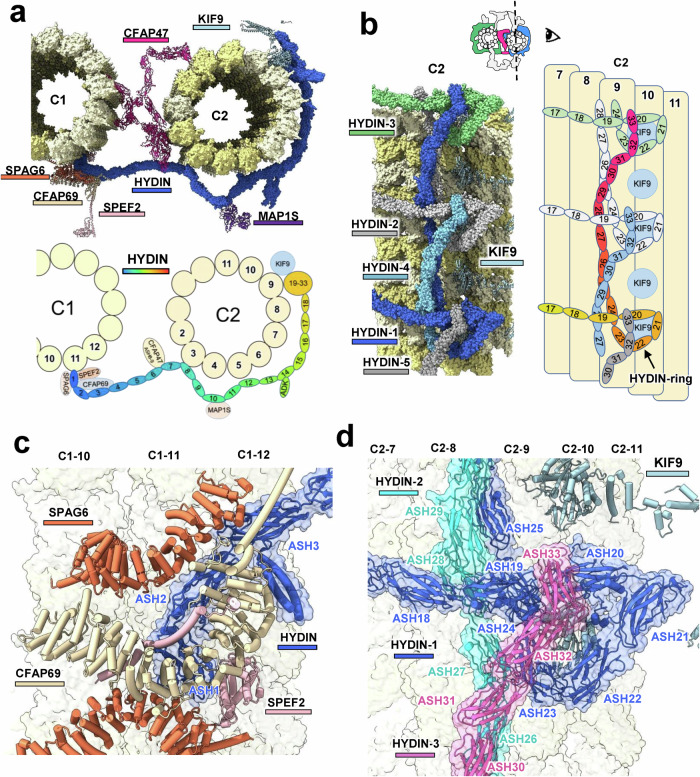
Full-length structure of HYDIN in mouse sperm CA. **a** Overall structure and schematic representation of HYDIN, shown in cross-sectional view. The numbers within the HYDIN structure indicate the order of the ASH domains. **b** Structure and schematic representation of the HYDIN CTD from the side view, highlighting the ASH17–33 domains. Different HYDIN proteins are shown in distinct colors. **c** Detailed view of the interactions between the HYDIN NTD and the C1 microtubule. **d** Detailed view of the interactions within the HYDIN CTD trimer on the C2 microtubule. The three HYDIN proteins are displayed in different colors, with their ASH domains labeled.

CFAP47 and HYDIN, two long-chain ASH domain-containing proteins, were previously poorly characterized due to their limited structural information within the CA. Here, we present the first full-length structures of both proteins, revealing their unique architectural features and interactions that contribute to the stabilization of C1 and C2 microtubules within the CA. HYDIN, the longest CA protein with 33 ASH domains and over 5000 residues (Supplementary information, Fig. S24), adopts a semicircular chain conformation that encircles C1 and C2 microtubules (Fig. 3a). It directly interacts with CFAP47 at the bridge region and reinforces the CA architecture in both the circular and axial directions. Specifically, ASH1–18 domains promote C1–C2 connection, while ASH19–33 domains provide axial support (Fig. 3a, b).

The N-terminus of HYDIN, encircled by SPAG6, CFAP69, and SPEF2, originates from protofilament-11 of the C1 microtubule and extends toward the C2 microtubule (Fig. 3c). In the bridge region, the ASH6–7 domains of HYDIN interact with the ASH8–9 domains of CFAP47, forming the HYDIN–CFAP47 complex (Fig. 3a). The HYDIN ASH8 domain binds to protofilament-3 of the C2 microtubule, curving the ASH8–12 domains into a scaffold for the C2b projections, whereas the ASH13–18 domains extend laterally toward protofilament-8. Next, the ASH19–33 domains of HYDIN exhibit a unique 270-degree bend extending longitudinally along the C2 protofilaments (Fig. 3b). These domains exceed 32 nm in length, allowing three HYDIN proteins to overlap every 16 nm (Supplementary information, Fig. S25a, b). The ASH19–24 domains of HYDIN-1 create a triangular ring (HYDIN-ring), while ASH25–28 pass through the gap between the HYDIN-2 ring and the C2 microtubule, and ASH29–33 rest atop the HYDIN-3 ring (Fig. 3b). This arrangement tightly clamps each HYDIN ring with two additional HYDIN molecules at distances of 16 nm and 32 nm, resulting in a compact structure termed the HYDIN-ring complex, which further binds to the KIF9 globular domain on the C2 microtubule (Fig. 3d). The presence of the HYDIN-ring complex in various low-resolution CA models indicates its evolutionary conservation (Supplementary information, Figs. S4, S5).

Additionally, we identified the adenylate kinase-like (ADK) domain of HYDIN in C2b projections (Fig. 3a). This domain, which catalyzes the interconversion of adenosine phosphates, is positioned near the RS4 head interface and likely plays a regulatory role in nucleotide metabolism, given the presence of nucleotide-binding sites in the RS heads.^31,32^ Considering that axonemes utilize ~230,000 ATP molecules per beat cycle,^33^ they may be sensitive to local ATP/ADP ratio fluctuations, influenced by the proximity of the HYDIN-ADK domain and RS head.

### CFAP47 links C1 and C2 at the CA center

**Fig. 4:**
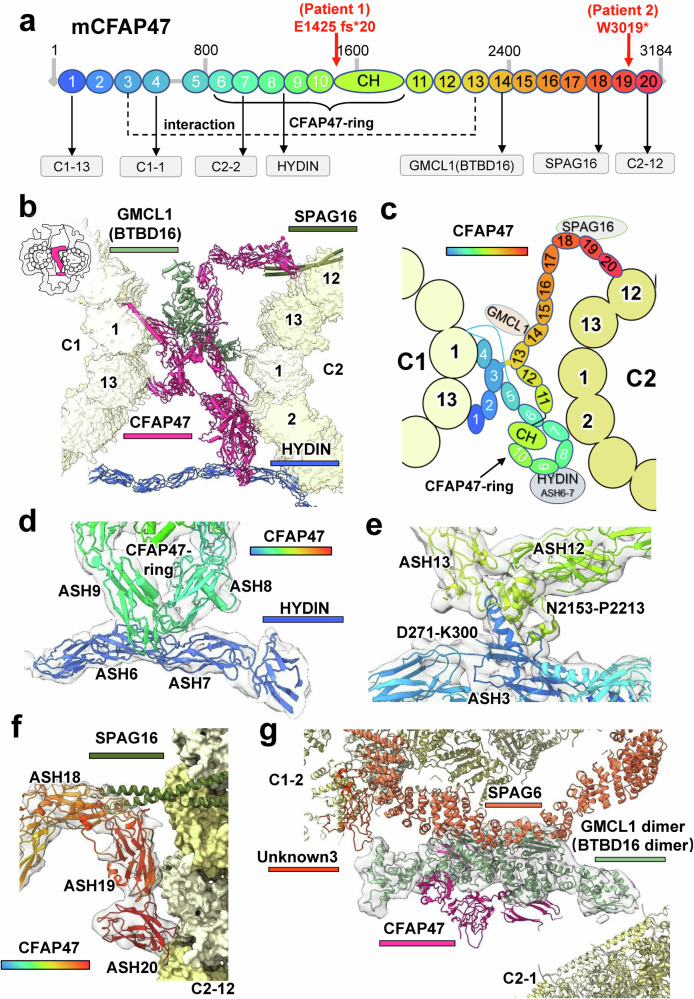
Full-length structure of CFAP47 in mouse sperm CA. **a** Schematic diagram of the domain composition of the mouse CFAP47 protein (mCFAP47). The numbers indicate the order of the ASH domains. The black arrows indicate binding proteins associated with the domains. The dashed lines represent interactions between the domains. The ASH6 to CH domains form the CFAP47-ring structure. Red arrows indicate the mCFAP47 amino acid positions corresponding to the truncation variants in patients 1 and 2. **b** The protein composition of the CA bridge within a 16-nm repeat. **c** Schematic representation of the CFAP47 structure and its interactions in the bridge. **d** Detailed view of the interactions between CFAP47 and HYDIN. **e** Detailed view of the interactions between the ASH3 and ASH13 domains of CFAP47. **f** Detailed view of the interactions between the CFAP47 CTD and the C2 microtubule. **g** Localization and structure of GMCL1 (BTBD16) dimer within the bridge.

In the CA structure, C1 and C2 are tightly connected through four parts: a central bridge, HYDIN, and two lateral connections between adjacent projections of C1a/C1b and C2a/C2b (Fig. 1b). While HYDIN wraps around C1 and C2 in an extended conformation, CFAP47 adopts a folded structure at the CA center (Fig. 4). CFAP47 comprises 20 ASH domains, with its NTD attaching to the C1 microtubule and extending to C2 through its middle domain and CTD (Fig. 4a). The ASH1 and ASH4 domains of CFAP47 bind to protofilament-13 and protofilament-1 of C1, respectively, reinforcing the C1 seam. From ASH5, CFAP47 bends toward the C2 microtubule. The ASH6–10 domains, along with a calponin homology (CH) domain composed of α-helices, form a stable structure known as the CFAP47-ring (Fig. 4b, c). This ring interacts with protofilament-2 of the C2 microtubule via its ASH7 domain and connects to the ASH6–7 domains of HYDIN through its ASH8–9 domains (Fig. 4d; Supplementary information, Fig. S25c, d). The remaining CFAP47 domains extend laterally along the C2 microtubule, with ASH13 forming stable interactions with the ASH3 domain. This arrangement enhances the linkage between the NTD and CTD of CFAP47, reinforcing the connection between C1 and C2 microtubules (Fig. 4e; Supplementary information, Fig. S25e, f). Additionally, the ASH18 domain of CFAP47 binds SPAG16, and its ASH20 domain interacts with protofilament-12 of the C2 microtubule (Fig. 4f). Most of the ASH domains of CFAP47 are connected by flexible loops, suggesting intrinsic flexibility in the overall structure. This configuration may help ensure the stability of the links between C1 and C2 during the rapid swimming of the sperm.

The density for another protein with approximate C2 symmetry is situated within the CA bridge, closely associated with CFAP47. DomainFit searches identified GMCL1 and BTBD16 — neither of which had been previously recognized as components of CA — as the top candidates, though their similar scores complicated precise identification (Supplementary information, Fig. S1^2^). Based on the better alignment of the predicted structure of GMCL1 with the observed density, along with its reported associations with spermatogenesis abnormalities,^34,35^ we provisionally designated it as a GMCL1 homodimer, denoted as GMCL1 (BTBD16), while also considering the alternative possibility of a BTBD16 homodimer or a GMCL1/BTBD16 heterodimer. GMCL1 (BTBD16) is a globular protein primarily composed of α-helices, with its CTD forming homodimers (Fig. 4g). It facilitates interactions between SPAG6 in C1-MOSP and ASH14 of CFAP47, strengthening the binding of CFAP47 to the C1 microtubule (Fig. 4g). Furthermore, the NTD of GMCL1 (BTBD16) interacts with an unidentified protein (termed Unknown3) on protofilament-2 of the C1 microtubule (Fig. 4g), highlighting its role in enhancing the structural integrity between C1 and C2 microtubules.

### In situ structure of CA from *Cfap47*-KO mice

**Fig. 5:**
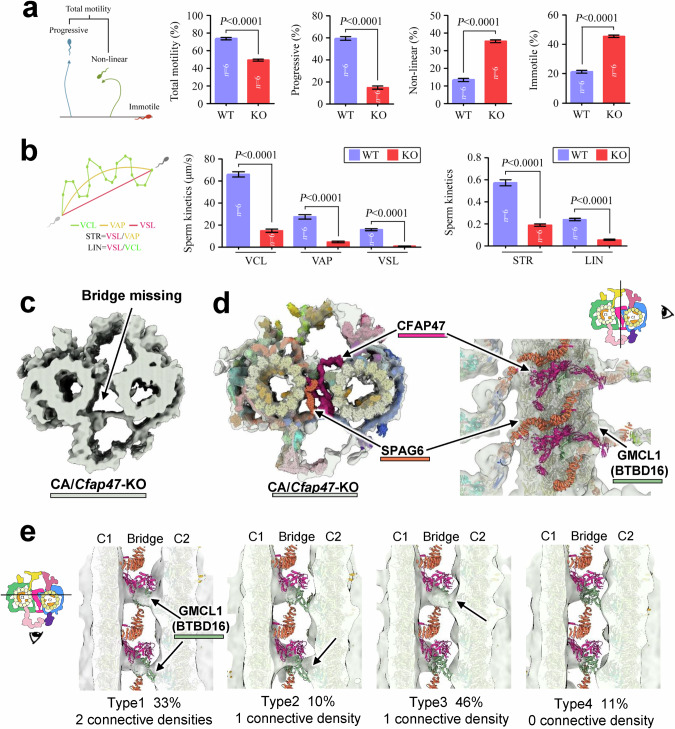
*Cfap47*-KO mouse sperm exhibits reduced progressive motility and impaired bridge density in the CA structure. **a** Analysis of semen characteristics via a CASA system to compare total motility, progressive motility, nonlinear motility, and immobility between WT and *Cfap47*-KO (KO) mice. **b** Measurement of VCL, VAP, and VSL of the sperm from WT and *Cfap47*-KO mice. Straightness (STR) is defined as VSL/VAP, and linearity (LIN) is defined as VSL/VCL. For all statistical analyses in this figure, Student’s *t*-test was used, and the error bars represent the SD (*n* = 6). **c** The overall structure of the CA in *Cfap47*-KO mice. The black arrow indicates the missing bridge density. **d** The WT CA model was fitted into the CA structure of *Cfap47*-KO mice, providing both top and side views from the bridge region. *Cfap47*-KO mice lack density associated with the CFAP47 and SPAG6 proteins in the bridge region of the CA structure. **e** Four types of CA structures in the *Cfap47*-KO dataset. In each 32-nm repeat, the two connective densities of the GMCL1 (BTBD16) proteins remaining in the bridge area (black arrow) gradually diminished. The proportions of different types in the dataset are indicated below. Raw data can be found in Supplementary information, Table S11.

To validate the role of CFAP47 in CA assembly and function, we generated *Cfap47*-KO mice by targeting exons 2–50 of the *Cfap47*-205 transcripts via CRISPR-Cas9 (Supplementary information, Fig. S26a). Sanger sequencing confirmed a 171,604-bp deletion, including 7249 bp of the coding sequence (Supplementary information, Fig. S26b). Real-time quantitative PCR (qRT-PCR) revealed nearly complete loss of *Cfap47* mRNA in the testes of *Cfap47*-KO mice (Supplementary information, Fig. S26c). While *Cfap47*-KO mice were otherwise healthy, males display drastically reduced fertility, as evidenced by a lower percentage of two-cell embryos produced from *Cfap47*-KO sperms than from wild-type (WT) sperms (Supplementary information, Fig. S26d). Despite similar sperm counts and morphologies (Supplementary information, Fig. S26e–g), *Cfap47*-KO sperms exhibited significantly reduced motility, including total and progressive motility, along with increased proportions of nonlinear and immotile sperms (Fig. 5a; Supplementary information, Video S2). Additionally, the kinematic parameters — curvilinear velocity (VCL), average path velocity (VAP), and straight-line velocity (VSL) — were markedly reduced in *Cfap47*-KO sperms compared to WT sperms (Fig. 5b). This phenotype, characterized by asthenozoospermia with normal morphology, is hereafter referred to as ANM.

We subsequently resolved the in situ structure of the sperm CA from *Cfap47*-KO mice. In the raw tomogram, the mutant axoneme exhibited a regular “9 + 2” structure, but the bridge region appeared more vacant than that in WT mice (Supplementary information, Fig. S26h). Upon data processing, the structure of the mutant CA revealed increased structural heterogeneity (Supplementary information, Fig. S27). Ideally, 25% of CA particles selected at 8 nm intervals contribute to the 32 nm repeat reconstructions. While 18% of the WT CA particles contributed to a 32-nm repeat structure, only 11% contributed to the structure in *Cfap47*-KO dataset (Supplementary information, Fig. S27), indicating a widespread structural impairment in the mutant CA.

After STA analysis, the mutant CA structure was resolved to a resolution of 25 Å (Supplementary information, Fig. S27). Overall, it largely resembles the WT structure, except for the missing density in the bridge region (Fig. 5c; Supplementary information, Fig. S28). The mutant structure confirmed the absence of the entire CFAP47 density (Fig. 5d). However, GMCL1 (BTBD16) remained present, indicating that GMCL1 (BTBD16) might independently stabilize the C1‒C2 linkage even without CFAP47. Moreover, the absence of the SPAG6 proteins in the CA bridge, which coincides with the absence of CFAP47, suggests that the stability of these SPAG6 proteins depends on the presence of CFAP47 (Fig. 5d). Further analysis revealed that the remaining bridge region connectivity in the CA of *Cfap47*-KO sperm is unstable. Each 32-nm repeat has two connective densities corresponding to GMCL1 (BTBD16). In the *Cfap47*-KO CA, only 33% of the particles retained both connective densities, while 56% retained one and 11% lost both (Fig. 5e). Different degrees of CA damage may lead to irregular sperm movement or even immotility. In addition, in the *Cfap47*-KO CA, the edge distance between C1 and C2 microtubules displayed increased dynamics (10.71 nm ± 1.45 nm vs 10.34 nm ± 0.44 nm, *P* < 0.0001), confirming that *Cfap47* KO reduces CA structural stability (Supplementary information, Fig. S28c).

In *Cfap47*-KO mice, cilia from the ependyma, trachea, and nasal mucosa revealed no discernible changes in either ciliary number or morphology (Supplementary information, Fig. S29). Considering a recent study suggesting a role for CFAP47 in polycystic kidney disease,^36^ we examined the kidney histology of 10-week-old *Cfap47*-KO mice and found no apparent abnormalities (Supplementary information, Fig. S29d). Thus, CFAP47 likely has tissue-specific roles in sperm flagella and a weak effect on other cilia, similar to the RS protein IQUB.^37^

Therefore, the structural comparison between WT and mutant CA provides insights into the molecular mechanism by which *CFAP47* deletion leads to the ANM phenotype in sperm. Although CFAP47 is not essential for CA assembly or for direct interactions with the 9 neighboring RSs, its absence disrupts the bridge structure, including the assembly of SPAG6 and GMCL1 (BTBD16), thereby weakening the connections between C1 and C2. This disruption potentially reduces the efficiency of force transmission during axoneme movement and increases structural damage over time. The distinct structural changes observed in the CA of *Cfap47*-KO mice underscore the importance of *CFAP47* in axoneme/CA function, explaining the fundamental molecular basis of ANM phenotype in the sperm.

### CFAP47 truncations in two infertile men

**Fig. 6:**
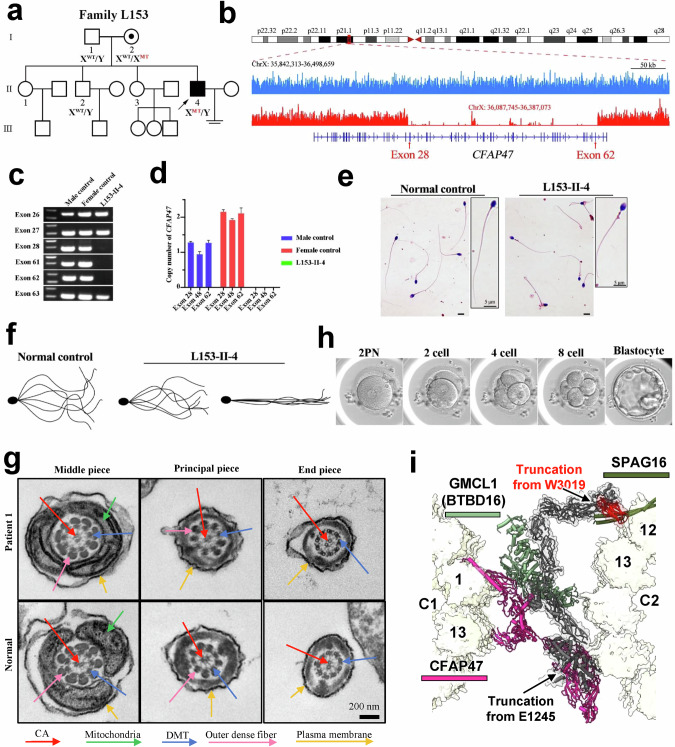
*CFAP47* deficiency in Patient 1 results in asthenozoospermia with normal sperm morphology. **a** Pedigree of Patient 1 affected by *CFAP47* deficiency. Male family members are denoted by squares, and female family members are denoted by circles. Patient 1 is indicated with a black-filled square and a black arrow. WT wild type, MT mutant. **b** Validation of the ~300-kb deletion on the X chromosome of Patient 1 (red). WGS revealed the location and length of the microdeletion covering exons 28 to 62 of the *CFAP47* gene. The normal control CFAP47 is shown in sky blue. **c** Agarose gel electrophoresis images of the PCR products from Patient 1 and normal male and female controls. The primer pairs for exons 26, 27, 28, 61, 62, and 63 confirmed the absence of exons 28 to 62 in Patient 1. **d** Relative copy number of the *CFAP47* gene in Patient 1, normal males and female controls. The beta-2-microglobulin (*B2M*) gene was used as an internal control. **e** Representative images of Papanicolaou-stained sperm from Patient 1 and the normal control. The staining experiments were performed three times. Scale bars, 5 μm. **f** The sperm flagellar waveform pattern of Patient 1 and the normal control. The sperm flagellar waveform of Patient 1 was abnormal, with reduced curvature and amplitude. **g** TEM assay of spermatozoa from Patient 1 and a normal human. Scale bar, 200 nm. **h** The development process of one MII oocyte after intracytoplasmic sperm injection. **i** Structural representation of the CFAP47 truncations in patients 1 and 2. The truncated portion for Patient 2 is shown in red, whereas the truncated portion for Patient 1 is shown in black (including the red portion).

To further investigate the pathogenesis of CA-related asthenozoospermia, we recruited a total of 320 infertile Chinese males diagnosed with asthenozoospermia for whole-exome sequencing (WES) analysis. In addition to previously known genetic variants associated with multiple morphological abnormalities of the sperm flagella (MMAF) and PCDs, we identified 35 single nucleotide variations (SNVs) or copy number variations (CNVs) in the *CFAP47* gene. Notably, an exon deletion in Patient 1 and a nonsense mutation in Patient 2 were strongly linked to asthenozoospermia (Supplementary information, Table S4). Patient 1, proband L153-II-4, a 31-year-old male from a nonconsanguineous family (Fig. 6a), presented normal hormone levels, testicular volume, and chromosome karyotype, and had no Y chromosome microdeletions. Whole-genome sequencing (WGS) revealed a microdeletion from 36,087,745 to 36,387,073 (GRCh37) on the X chromosome, affecting exons 28 to 62 of the *CFAP47* gene (Fig. 6b). This deletion fragment was confirmed by both PCR and qPCR (Fig. 6c, d). This deletion, which was not previously reported or included in the human database of genomic variants, results in the loss of 4867 bp in the coding region of *CFAP47*, producing a truncated protein Asp1440Glufs*20. Patient 2, a 33-year-old male from an unrelated family, exhibited a c.9069G>A mutation, leading to a truncated protein p.W3023*.

For Patient 1, all semen parameters were normal per the World Health Organization (WHO) guidelines (Supplementary information, Table S5). However, the progressive motility rate of the spermatozoa decreased dramatically to 7.6% ± 5.1%, with rapid progressive motility at 2.4% ± 3.3% and slow progressive motility at 5.3% ± 1.8% (Supplementary information, Video S3). The kinematic parameters of the sperm, including VCL, VSL, and VAP, were significantly lower than those of control males. Similar clinical phenotypes were also observed in Patient 2. Despite normal morphology under Papanicolaou staining (Fig. 6e), the sperm flagella waveform pattern showed abnormal curvature and amplitude (Fig. 6f). Transmission electron microscopy (TEM) revealed an apparently normal “9 + 2” axoneme structure (Fig. 6g). Assisted reproductive technology, specifically intracytoplasmic sperm injection (ICSI) for Patient 1 and his wife, resulted in successful fertilization of 11 metaphase II (MII) oocytes, yielding 9 transferable embryos (Fig. 6h; Supplementary information, Table S6).

Consequently, the significant truncation of the *CFAP47* gene in Patient 1 and Patient 2 resulted in the ANM phenotype of sperm, mirroring the phenotypes observed in *Cfap47*-KO mice. Our complete structural model of CFAP47 illuminates the pathogenesis of asthenozoospermia in both patients 1 and 2. In Patient 1, more than half of the CFAP47 protein’s sequence was truncated (Fig. 4a), resulting in the loss of domains spanning from the CH domain to the ASH20 domain. This truncation is presumed to disrupt the CFAP47-ring structure and ASH3–ASH13 interactions, eliminating the external binding of CFAP47 with GMCL1 (BTBD16) and SPAG16 (Fig. 6i), which weakens connections between C1 and C2, leading to reduced sperm motility. In Patient 2, the mutation is presumed to affect ASH19 and leads to the loss of ASH20 (Fig. 4a), preventing CFAP47 from binding to protofilament-12 of C2 (Fig. 6i), thereby weakening the connection between C1 and C2 and resulting in decreased sperm motility.

### Structural basis of CA-related human diseases

**Fig. 7:**
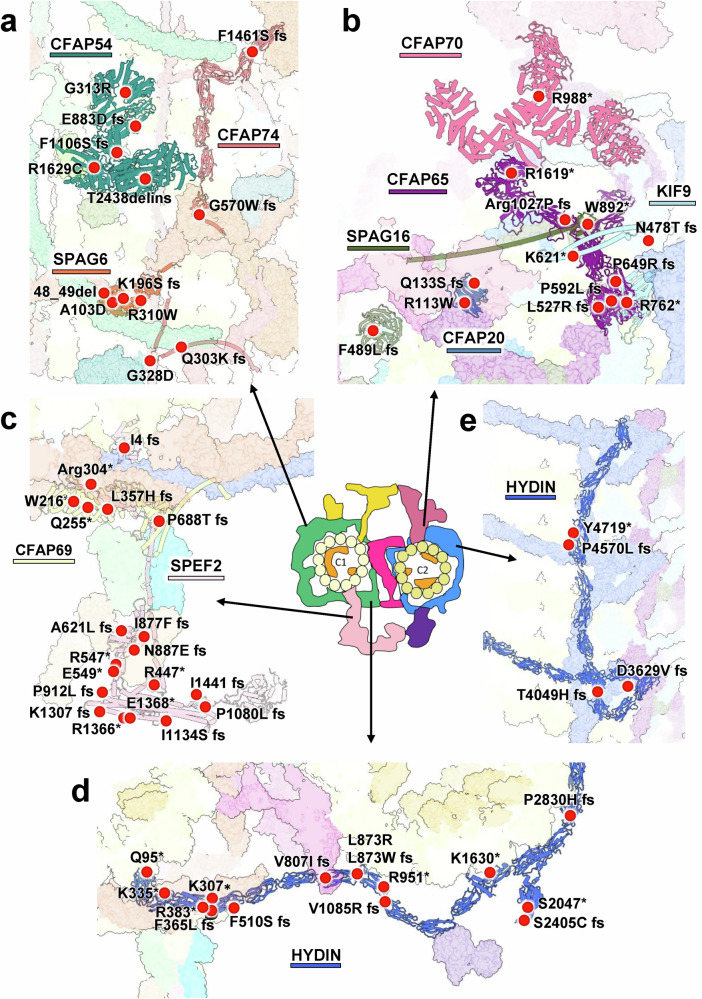
Mapping of human MMAF- and PCD-associated mutations onto the mouse sperm CA structure. **a**–**e** The CA components in various regions are shown in an enlarged view, including the C1-MOSP (**a**), C2-MOSP and C2a projections (**b**), C1b projection (**c**), HYDIN NTD (**d**), and HYDIN CTD (**e**). The locations of these human mutations are indicated by red circles on the structure on the basis of sequence alignment. Each mutation label contains a single-letter code for the residue and the associated human protein sequence number. fs frameshift, del deletion, ins insertion. An asterisk (*) denotes sequence termination, and splicing mutations are not shown.

Alignment of our structural model with the human CA map revealed a high degree of congruence, except for some SPACA9 proteins in the C2 lumen (Supplementary information, Fig. S30). These findings indicate strong structural and functional conservation of CA components between mouse and human. Thus, our CA structure offers a fundamental framework for studying mutations implicated in human ciliopathies (Fig. 7; Supplementary information, Table S7). In C1-MOSP, mutations associated with human PCDs primarily occur in SPAG6, CFAP54, and CFAP74 (Fig. 7a). SPAG6 is crucial as a structural scaffold, and its deficiency causes male infertility and hydrocephalus.^38^ In C2-MOSP, mutations mainly affect SPAG16, KIF9, and CFAP20 (Fig. 7b), among which KIF9 mutations notably impair sperm motility.^39^ In large CA projections, such as C2a and C1b, mutations occur in CFAP65, CFAP70, SPEF2, and CFAP69 (Fig. 7b, c). SPEF2, which is essential for C1b formation, presents mutations that result in immotile sperm and PCD.^40^

HYDIN has numerous mutation sites in the NTD connecting C1 and C2 and in the CTD within C2-MOSP (Fig. 7d, e), underscoring its critical role in CA assembly. Biochemical analysis revealed that HYDIN interacts with CPC1 (mouse SPEF2) and KLP1 (mouse KIF9),^41^ and the absence of HYDIN results in a complete loss of detectable KIF9.^39^ RNA interference targeting HYDIN in *Chlamydomonas* results in short flagella lacking the C2b projection,^41^ and the deficiency of HYDIN is linked to congenital hydrocephalus and fetal death in mice,^42^ both of which align with the involvement of HYDIN in the assembly of C1b, the bridge, C2b, and C2-MOSP (Fig. 3).

Overall, alterations in CA components — residue changes or protein truncations — affect CA function in two primary ways. First, mutations in MOSP components and projections, such as CFAP45, CFAP65, SPEF2, and HYDIN, can disrupt CA interactions with RS heads. Second, mutations in scaffold proteins, such as CFAP47 and HYDIN, can weaken the connections between C1 and C2, compromising CA stability. CFAP47 mutations have a milder impact on CA function since they do not directly affect CA‒RS interactions, but they weaken C1‒C2 connections, resulting in the ANM phenotype. Conversely, HYDIN truncations can eliminate multiple CA‒RS binding sites, leading to severe conditions or fetal death. Our CA structure elucidates these pathogenic mechanisms underlying CA-related diseases.

## Discussion

The axoneme in motile cilia and flagella is a complex structure essential for propulsion and fluid transport, with the CA serving as a core component. Limited data on CA structures hamper the understanding of CA-related pathologies, such as CFAP47 truncations linked to the ANM phenotypes in patients 1 and 2. Our visual proteomics approach generated a near-complete model of the mouse sperm CA, identifying 39 CA-associated proteins including eight new components: GRK3, ANKMY1, LRRC43, GOT1L1, LRRD1, MAP1S, GMCL1(BTBD16), and SPACA9. Notably, long chain-like proteins HYDIN and CFAP47 form flexible links between C1 and C2 microtubules. This model enhances our understanding of how CA mutations, such as those in CFAP47 and HYDIN, contribute to human diseases, providing essential insights into mammalian CA assembly and motility. Our study presents an integrated research methodology that combines clinical data, cryo-ET, visual proteomics, and animal models to elucidate molecular pathogenesis.

The asymmetric periodicities of the C1- and C2-MOSPs in the CA structure are driven by differences in protein composition, especially the ASH proteins. In the C1-MOSP, SPAG6 forms a 16-nm repeating scaffold, while components such as CFAP54, CCDC180, CFAP99, and CFAP46 occupy the position of SPAG6 on the side away from C2, leading to a 32-nm repeat. Additionally, the ASH protein DLEC1 contributes to the 32-nm repeat and serves as the foundation for the C1e projection. In the C2-MOSP, components like CFAP20, SPAG16, and KIF9 establish a 16-nm repeat, with the long ASH protein HYDIN forming a unique HYDIN-ring complex that preserves this periodicity. These asymmetries in C1- and C2-MOSPs are likely associated with axonemal movement patterns and the asymmetric beating of cilia and flagella,^43^ as the CA plays a key role in modulating these motions. The distinct molecular composition across the CA MOSPs may generate variations in the CA‒RS interaction forces for each DMT, leading to asymmetric and non-planar beating. Further comprehensive analysis of the entire asymmetric axoneme structure and systematic dynamic simulations will deepen our understanding of this mechanism.

Studying large biomacromolecules such as CA is challenging because of difficulties in recombinant expression and maintaining structural integrity during purification, which hinders detailed analysis of pathological mutants. Traditional techniques like TEM, though widely used for analyzing sperm defects, are limited by lower resolution and potential artifacts. In contrast, cryo-ET preserves samples in their native state, prevents structural damage caused by cell disruption and purification, and provides higher-resolution insights into CA pathogenesis. Here, we successfully resolved the mutant CA structure via 95 tomograms (one-day cryo-EM time) with less than 1% mouse sperm, revealing the impact of *Cfap47*-KO on the CA structure. Thus, cryo-ET holds promise for in situ disease diagnosis, enabling the visualization of molecular mechanisms underlying pathologies.

Previous studies on the in situ structure of CA have typically achieved resolutions below 2 nm (Supplementary information, Fig. S5), limiting detailed molecular insights. To address this, we implemented several technical optimizations. First, we employed a sperm sample preparation method as previously reported,^17^ which preserves the integrity of the axoneme structure in frozen samples, preventing axoneme deformation. Second, we carefully aligned tilt series to enhance tomogram quality, rigorously excluding low-quality data. Finally, we used state-of-the-art software tools such as AreTOMO^44^ and Warp/M^45,46^ to improve the resolution of in situ structural analysis. These advancements enabled us to significantly improve the resolution of the CA structure.

The integration of visual proteomics with cryo-ET enables the modeling of target proteins at a moderate resolution. However, this approach is limited by AlphaFold2 predictions, map quality, and MS precision. For example, accurate identification of structurally similar proteins, such as GRK3/GRK2 (Supplementary information, Fig. S9), GOT1L1/GOT2/GOT1 (Supplementary information, Fig. S10), GMCL1/BTBD16 (Supplementary information, Fig. S12), and PPP1CC/PPP1CA/PPP1CB (Supplementary information, Fig. S14), requires future studies. As instrumentation and computational methods continue to advance, the resolution of in situ structural analysis for large macromolecular complexes, such as CA and DMT, is expected to improve further, potentially reaching near-atomic levels. This progress will provide deeper insights into the molecular mechanisms underlying sperm motility and related pathologies.

The CA can rotate within the axoneme during ciliary beating in many protists like *C. reinhardtii*, but it does not exhibit rotation in the flagella and cilia of metazoan animals.^10,47,48^ The molecular mechanism behind this species-dependent difference in CA rotation remains largely unresolved. It has been reported that in the flagella and cilia of metazoan animals, the RSs of DMT3 and DMT8 are permanently connected to the CA, acting as partitions.^49^ Moreover, in the mouse sperm axoneme, while most RS heads are positioned at a slight distance from the CA, protrusions from the CA were observed extending into the head of RS1/2/3 of DMT8.^14^ Such complementary shapes may limit the lateral movement of DMT8 and stabilize its radial position. Interestingly, our structure reveals that, compared to the CA of *C. reinhardtii*, the mouse CA contains unique components such as GRK3 and ANKMY1 in the RS8-binding region (Fig. 1; Supplementary information, Fig. S20). These components may play a crucial role in reinforcing the connection between DMT8 and the CA. Future studies will require optimized sample preparation techniques to resolve high-resolution structures of the RS‒CA interface to better understand these interaction details.

The RS heads have highly negatively charged distal surfaces,^32^ yet electrostatic surfaces of CA projections and MOSPs show mostly scattered regions of positive and negative charge (Supplementary information, Fig. S31), indicating that CA and RS may not form stable complexes through opposite charges. In contrast, the predominantly negative charges on C1b and C2a support a model of electrostatic repulsion between RS and CA when they are in proximity during beating.^32^ Moreover, previous studies on the *C. reinhardtii* CA suggested that CFAP47 density enables sliding and rotation between C1 and C2, with the HYDIN density acting as a motor arm capable of sliding along microtubules. Subsequently, KLP1 arrays are proposed to transmit mechanical force, influencing CA‒RS interactions.^11^ However, our cryo-ET data did not reveal sliding or rotation between C1 and C2 or HYDIN-CTD movement along the C2 microtubule. This stable C1‒C2 connection aligns with the reported in situ CA structures from other species^15^ (Supplementary information, Fig. S5), suggesting that previous in vitro observations^11^ may stem from sample preparation artifacts or reflect inherent differences between species.

Numerous reports have demonstrated a close association between CA components and asthenozoospermia. For example, recent studies have identified CFAP47 mutations in asthenozoospermia patients, highlighting the crucial role of CFAP47 in flagellar structure and its potential as a diagnostic marker for this condition.^50–52^ Nevertheless, without a complete CA structure at hand, previous investigations were largely unable to clarify the assembly and function of the target protein, nor could they uncover the pathogenic mechanism underlying asthenozoospermia. The CA structure that we have uncovered not only corroborates previous findings, but also offers a novel avenue for exploring the relationship between various CA components and disease phenotypes, especially for those newly identified CA components in our structure.

AlphaFold2 has revolutionized structural modeling by leveraging secondary structure information from medium-resolution cryo-EM reconstructions.^53^ This approach enables the modeling of both known and novel protein components in their native states without the need of labeling or cellular disruption, which is termed visual proteomics.^16^ In this study, we employed this technology to construct near-complete models of 39 CA components within native mammalian sperm, deepening our understanding of protein‒microtubule interactions and CA assembly mechanisms. Our work establishes a framework for investigating the molecular mechanisms underlying human ciliopathies.

## Materials and methods

### Study participants and ethical approval

A total of 320 infertile Chinese males with asthenozoospermia were recruited at the Center for Reproductive Medicine, Women and Children’s Hospital of Chongqing Medical University from August 2021 to July 2024. The detailed inclusion criteria were as follows: primary infertility; decreased sperm progressive motility (≤ 20%); normal chromosome karyotype (46, XY) without Y chromosome microdeletions; and no other identified pathogenic factors, such as absence of vas deferens, varicocele, or viral infection. Written informed consent was signed prior to the collection of blood and semen samples. The ethical approval (No.: (2023) Ethics Review (Research) 030) was granted by the Ethics Committee of Chongqing Health Center for Women and Children.

### WES

Genomic DNA (gDNA) was extracted from peripheral blood samples using a QIAamp DNA blood mini kit (Qiagen, Netherlands). After that, the gDNA was treated with a SureSelect Human All Exon V6 Kit (Agilent Technologies, USA) and sequenced on the NovaSeq 6000 platform (Illumina). Clean readings were filtered using Burrows Wheeler Aligner (BWA) and aligned with the human reference genome (GRCh37/hg19).^54^ SNVs and insertion-deletion variants (InDels) were named using the Genome Analysis Toolkit (GATK)^55^ and functionally annotated using ANNOVAR.^56^ CNVs were called by DNA copy R package,^57^ and the genes included in the CNV interval were annotated by ClinVar database (https://www.ncbi.nlm.nih.gov/clinvar/), Decipher database (https://www.deciphergenomics.org/), and Online Mendelian Inheritance in Man database (OMIM, https://www.omim.org/). Moreover, PolyPhen-2 (http://genetics.bwh.harvard.edu/pph2/), Sorting Intolerant from Tolerant (SIFT, http://sift.bii.a-star.edu.sg/), MutationTaster (https://www.mutationtaster.org/), and Combined Annotation Dependent Depletion (CADD, https://cadd.gs.washington.edu/) were applied in the prediction of clinical significance of variants. The gnomAD database (http://gnomad-sg.org/) and VarSome (https://varsome.com/) were searched for the frequencies of variants in different populations and previously reported variants.

### WGS, PCR and qPCR

For WGS, gDNA extracted from peripheral blood samples was randomly fragmented using an ultrasonicator Q800R (Qsonica, USA). Subsequently, the constructed gDNA library was sequenced on the AmCareSeq-2000 platform (AmCare Genomics, China) with a sequencing strategy of 200–500-bp insertion size and 150-bp paired ends. As described in WES, the raw data were cleaned by discarding reads with base mass lower than QC20 and aligned with the human reference genome (GRCh37/hg19) using BWA. The mean and standard deviation of the proband’s WGS depth are 5.74 ± 1.56. The deletion of *CFAP47* in the human genome was validated by PCR using primers covering exons 26, 27, 28, 61, 62, and 63. Meanwhile, the copy number of *CFAP47* gene in gDNA was evaluated through qPCR with specific primers of exons 28, 48 and 62 using TB Green Premix Ex Taq II (Takara, Japan) on an ABI 7500 (Thermo Fisher Scientific, USA), and analyzed using the 2^−∆∆Ct^ method by normalizing to that of beta-2-microglobulin gene as the inner control. The primers used were listed in Supplementary information, Table S8.

### Semen analysis and Papanicolaou staining

According to the WHO guidelines,^58,59^ fresh semen samples were collected through masturbation after 2–7 days of abstinence and evaluated after liquefaction at 37 °C for 30 min. A computer-aided sperm analysis (CASA) system (Jiangsu Rich Life Science Instrument, China) was applied to evaluate the semen parameters, such as sperm concentration, sperm motility, etc. For Papanicolaou staining, the washed sperm samples were mounted on glass slides, air-dried, and fixed with 95.0% ethanol for 3 min. Then, the slides were stained using an improved Papanicolaou staining kit (Cariad Medical Technology, USA), including immersion in hematoxylin for 3 min, acidic ethanol for 5 s, and eosin and bright green for 3 min, respectively. More than 200 stained sperms were observed under an optical microscope TCS SP8 (Leica, Germany).

### Ovarian stimulation and fertilization

A short-acting GnRH-agonist (GnRH-a) long protocol was used for controlled ovarian stimulation. The spouse of Patient 1 (L153-II-5) received a daily subcutaneous injection of 0.1 mg short-acting GnRH-a decapeptyl (Ferring) from the midluteal phase of the preceding menstrual cycle for down-regulation of the pituitary gland. After 20 days of down-regulation, the levels of serum estradiol, luteinizing hormone (LH), and progesterone were less than 10 pg/mL, 5 mIU/mL, and 0.9 ng/mL, respectively. Then the gonadotrophin, recombinant follicle-stimulating hormone (rFSH), was administered with an initial dose of 150 IU, while monitoring serum hormone changes and follicle growth trends. Recombinant human chorionic gonadotropin (hCG) (Merck Serono, Germany) at a dose of 250 mg was administered to trigger the maturation of oocytes, and oocyte retrieval was performed at 34–36 h later. Consequently, 13 oocytes were obtained and all were in MII stage. After cumulus cell removal, ICSI was performed and the oocytes were transferred to G1-plus medium (Vitrolife, Sweden) droplets for further culture. A time-lapse monitoring system Embryoscope Plus (Vitrolife) was used for embryo culture and the embryo quality assessment was performed according to the previously reported criteria.^60,61^

### Mouse sperm extraction

To extract sperm from mouse, a 200 μL sperm capacitating solution^17^ was dispensed as droplets into a 35-mm dish, covered with mineral oil, and then incubated at 37 °C in a 5% CO_2_ environment for at least 30 min. Twelve-week-old C57 male mice were sacrificed via cervical dislocation, and the epididymis was carefully dissected. Subsequently, sperm retrieval was performed under a stereomicroscope, and the sperm pellets were transferred into a capacitating solution. The sperm pellets were then subjected to an incubation period exceeding 30 min until complete dispersion was achieved. The resulting sperm solution was aspirated and transferred into a 1.5-mL tube, where it was temporarily stored on ice prior to subsequent experimental procedures.

For WT mice, the animal experimentation was conducted at the Laboratory of the Animal Center, Institute of Biophysics, Chinese Academy of Sciences, in strict adherence to the National Institutes of Health Guide for the Care and Use of Laboratory Animals and the approved guidelines of the Institutional Animal Care and Use Committee at the Institute of Biophysics, under the leadership of Dr. Guangxia Gao as the committee chair. For gene knockout mice, the animal experiments were approved by the Animal Care and Use Committee of the College of Life Sciences, Beijing Normal University (2024-SW-001).

### Preparation of mouse sperm for cryo-EM analysis

Freshly harvested sperm cells were subjected to centrifugation at 4 °C and 400 G using a Legend Micro 17 R centrifuge (Thermo Fisher Scientific) for 5 min. The resulting pellet from 100 μL of the sperm solution was gently resuspended in 100 μL of pre-cooled PBS on ice, and subsequently diluted by a factor of 5.5 with PBS before immediate usage. A R3.5/1, Au 200 mesh cryo-EM grid (Quantifoil, Germany) was glow discharged for 60 s using a Solarus device (Gatan, Sweden). The sperm sample was vitrified employing either an EM GP (Leica, Germany) or EM GP2 (Leica) instrument. Subsequently, 2.7 μL of the PBS-diluted sample was applied onto the grid, rapidly blotted for 2–5 s at 100% relative humidity and 4 °C, then plunge-frozen in liquid ethane cooled to –186 °C, and stored in liquid nitrogen prior to cryo-FIB milling.

To prepare sperm lamellae, an Aquilos 2 SEM system (Thermo Fisher Scientific) was utilized. The sample underwent sequential coating with a platinum layer for 10 s, an organometallic platinum layer for 15 s, and a final platinum layer for 10 s using default parameters. Following the identification of optimal milling positions, a stepwise tilting and milling approach was executed to produce lamellae with a target thickness of ~200 nm. Once the desired thickness was achieved, the grid underwent sputtering with a final platinum layer at 30 KV, 10 mA for 1–3 s, and was then stored in liquid nitrogen in preparation for data collection in TEM studies.

### Cryo-ET data acquisition

The data collection procedures adhered to the methods reported in our prior publication.^17^ In summary, for WT sperm sample, grids were mounted onto an Autoloader and inserted into a Titan Krios G3 300 kV TEM (Thermo Fisher Scientific), equipped with a Gatan K2 direct electron detector (DED) and a BioQuantum energy filter. Tilt series were acquired at a magnification of 81,000×, yielding a physical pixel size of 1.76 Å (0.88 Å in super-resolution mode) on the K2 DED. Prior to data collection, the pre-tilt of the sample was visually assessed and set to either +10° or –9° to accommodate the pre-determined geometry induced by grid loading. The cumulative dose was fixed at 3.5 electrons/Å^2^ per tilt, divided into 10 frames over a 1.2-s exposure. The tilt range was established between –66° to +51° for a –9° pre-tilt or –50° to +67° for a +10° pre-tilt, with 3° increments, resulting in 40 tilts and a total of 140 electrons per tilt series. The slit width was maintained at 20 eV, with zero-loss peak refinement performed after each tilt series was collected. The nominal defocus was set between –1.8 μm and –2.5 μm. Tilt series acquisition utilized a dose-symmetry strategy facilitated by a beam-image-shift approach, implemented through in-house developed scripts within the SerialEM software.^62,63^ The data for the *Cfap47*-KO sperm sample were collected in the same manner, with the only variation being a physical pixel size of 3.4 Å.

### Tomogram reconstruction and STA analysis

After data collection, all fractioned movies were imported into Warp^45^ for initial processing, including motion correction, binning of the super-resolution frames by a factor of two, CTF estimation, masking of platinum islands or other high-contrast features, and tilt series generation. The tilt series were then automatically aligned using AreTOMO.^44^ The aligned tilt series were inspected in IMOD,^64^ and frames of low quality (e.g., those showing evident crystalline ice or large shifts) were excluded to create new tilt series sets in Warp. This iterative process continued with AreTOMO alignment until no further frames needed removal. Tilt series with fewer than 30 frames or unsuccessful alignment were excluded from further processing. The alignment parameters for the remaining tilt series were transferred back to Warp, where initial tomogram reconstruction was performed at a pixel size of 14.08 Å for WT dataset and 27.2 Å for *Cfap47*-KO dataset. CA particles were manually picked using the filament picking tool in Dynamo,^65^ by marking the start and end points of each CA filament, with crops separated by 8 nm along the filament axis. Dynamo automatically generated the 3D coordinates and two of the three Euler angles (excluding in-plane rotation), which were then transferred back to Warp for sub-tomogram exporting.

Sub-tomogram refinement was performed in RELION 3.1.^66^ The transformation of RELION’s star file and Dynamo’s table file was accomplished using the ABTT package. For WT CA, initially, all particles were reconstructed in a box size of 84^3^ voxels at a pixel size of 14.08 Å, and a low-pass filtered initial reference at 60 Å was created by directly averaging all selected particles. 3D classification with restrictions on the first two Euler angles (--sigma_tilt 3 and --sigma_psi 3 in RELION) was carried out to obtain the 16-nm repeat map of CA, with duplicated particles in an 8-nm interval removed. The C1 microtubule-focused mask was applied in 3D classification to derive the 32-nm repeat map of CA, with duplicated particles in a 16-nm interval removed. Both the 16-nm and 32-nm repeat maps of CA were refined to the Nyquist limit resolution. The dataset for *Cfap47*-KO CA was processed in the same manner, resulting in a reconstruction map with a resolution of 25 Å.

Subsequently, data processing focused on different local regions of WT CA. Particles for local regions with variant symmetries were obtained from the corresponding CA map using shift center method in RELION. These aligned parameters were transferred back to Warp to export sub-tomograms at a pixel size of 7.04 Å. Following classification and 3D auto-refinement, if the reported resolution reached the Nyquist limit, sub-tomograms were extracted at a pixel size of 3.52 Å. If resolution approached the Nyquist limit again after further classification and 3D auto-refinement, sub-tomograms were extracted at a pixel size of 2.64 Å. Then, all the aligned parameters for particles in the local regions were transferred back to M^46^ for further alignment, resulting in reconstruction maps with resolutions ranging from 5.5 Å to 18 Å (Supplementary information, Figs. S2, S3). The data processing statistics are summarized in Supplementary information, Table S1. Visualization of cryo-EM maps was improved by EMReady.^67^ Image creation, and refined model rendering were conducted using UCSF Chimera^68^ and UCSF ChimeraX.^69^

### MS assay

Since we used the same biological samples from our previous study of mouse sperm DMT, we just performed further analyses using previously acquired MS data.^17^ These data were collected through liquid chromatography-tandem mass spectrometry (LC-MS) utilizing the nanoLC-Orbitrap Exploris 480 instrument. An updated UniProt Mouse Proteome database (updated to March 2024) was employed for the MS search and identification process. The SEQUEST HT search engine in Thermo Proteome Discoverer (version 2.4.1.15) facilitated the identification of proteins. The MS results from samples treated with various buffers were merged and presented as Supplementary information, Table S9.

### Model building

Modeling was performed for each asymmetric unit of CA components. Since most CA components from mouse sperm did not have resolved structures in the Protein Data Bank (PDB) at the time this manuscript was prepared, the predicted models from AlphaFold2^70^ were employed as initial models. Two distinct approaches were applied to generate the final models in our study.

On one hand, several proteins possess homologs that have been identified previously in *C. reinhardtii* CA.^11,12^ Utilizing the protein location information elucidated from this structure, the predicted models from AlphaFold2 were directly fitted into the corresponding density maps. In cases where significant conformational changes were observed, the models were flexibly fitted into the density using the ISOLDE plugin within ChimeraX^69^ or were manually adjusted using Coot.^71^ Subsequently, the adjusted models underwent further refinement with real_space_refine tool in Phenix.^72^ The models for tubulin α/β, SPAG6, CFAP46, CFAP74, CCDC180, CFAP99, CFAP221, LRRC72, DLEC1, CFAP69, SPAG16, KIF9, FAM228B, CFAP20, MYCBPAP, SPATA4, and CFAP65 were built in this manner (Supplementary information, Fig. S6). For proteins that exist as subcomplexes, their structures were predicted using AlphaFold2 Multimer^70^ prior to fitting. Models of the CFAP54–CaM complex, SPAG17–DPY30 complex, SPATA17–CaM complex, CFAP119–MORN2 complex, SPEF2–LRGUK complex, and the CFAP70 dimer were all constructed in this manner (Supplementary information, Fig. S6). CFAP47 and HYDIN, predominantly composed of ASH domains, form two extended chain-like structures. The local density map of our CA structure provided sufficient resolution to distinctly identify each ASH domain (Supplementary information, Figs. S7, S8). The NTDs of both proteins, identified in *C. reinhardtii* CA,^11,12^ were used as starting points for modeling. From these NTDs, we sequentially fitted each ASH domain predicted by AlphaFold2 into the density map, and successfully built all ASH domains (Supplementary information, Figs. S7, S8). Furthermore, we confirmed typical structural features by AlphaFold2 Multimer predictions, including the HYDIN_ASH6–7_–CFAP47_ASH8–9_ complex (Supplementary information, Fig. S25d), the ASH_2–4_–ASH_12–14_ complex of CFAP47 (Supplementary information, Fig. S25f), and the HYDIN-CTD trimer complex (Supplementary information, Fig. S25b). In addition, we identified several densities that were previously categorized as unknown proteins and only had main-chain models in *C. reinhardtii* CA structure.^11,12^ Subsequently, we incorporated these models into our CA structure and designated them as Unknown1 to Unknown6 proteins.

On the other hand, for proteins or domains not present in the reported model of *C. reinhardtii* CA,^11,12^ we employed the DomainFit tool^19^ to identify proteins in local maps. We utilized proteins from both our MS results (Supplementary information, Table S9) and previously reported proteomes of mouse sperm^18^ as candidates for DomainFit searches. Based on DomainFit scores and model-map cross-correlation (CC) rankings, we selected top-scoring candidate proteins for manual verification. In cases of similar structures within the same protein family, we prioritized proteins highly expressed in late spermatids (according to the Human Protein Atlas database) to build the model. If significant conformational changes were present, the model was flexibly fitted into the density map using the ISOLDE plugin in ChimeraX or manually adjusted using Coot. The adjusted models were refined further using real_space_refine tool in Phenix. Models of GRK3 (Supplementary information, Fig. S9), GOT1L1 (Supplementary information, Fig. S10), LRRD1 (Supplementary information, Fig. S11), GMCL1 (BTBD16) (Supplementary information, Fig. S1^2^), SPACA9 (Supplementary information, Fig. S13), and PPP1CC (Supplementary information, Fig. S14) were all constructed in this manner. Additionally, we made several improvements to the DomainFit tool, which has now been renamed DomainSeeker and will be published in a separate paper, in order to enhance protein identification efficiency. Briefly, we utilized the predicted aligned error (PAE) generated from AlphaFold2 to partition each protein structure into accurate and rigid units, thereby minimizing incorrect domain division. And we also standardized the correlation scores of overlapping regions between structure and density with comparable sizes, which we refer to as local z-scores. Regions with higher local z-scores exhibit superior local matching details. Models of CFAP99-NTD (Supplementary information, Fig. S15), ANKMY1 (Supplementary information, Fig. S16), and LRRC43 (Supplementary information, Fig. S17) were all built using this enhanced tool. Furthermore, FAP312 was previously identified as a C2 component through comprehensive proteome analysis comparing axoneme samples from *C. reinhardtii* with and without the CA complex.^73^ The Alphafold2-predicted structure of its homologous protein in mouse, MAP1S (Supplementary information, Fig. S18), was then utilized to search against the unidentified densities in C2 using DomainFit. As a result, we identified the position of MAP1S in the C2b projection and built its model.

### Generation of *Cfap47*-KO mice

The mouse *Cfap47* gene has 6 transcripts and is located on the chromosome X. Exons 2–50 of the *Cfap47*-205 (ENSMUST00000197180.6) transcript were selected as the knockout region. This region contains a 7249-bp coding sequence and knocking out the region will result in disruption of protein function. In this project, we used CRISPR-Cas9 technology to modify *Cfap47* gene. The brief process is as follows: gRNAs were transcribed in vitro. Cas9 (TriLink BioTechnologie) and gRNAs were microinjected into the fertilized eggs of C57BL/6JGpt mice. The injected eggs were transplanted into pseudopregnant recipients to obtain positive F0 mice which were confirmed by PCR. A stable F1-generation mouse strain was obtained by mating positive F0-generation mice with C57BL/6JGpt mice. Confirmation of the desired mutant allele was carried out by PCR. Primers for genotyping: 5’-AGAGACTAGGTACGGCAAAGCTGA-3’ and 5’-CCAAGACAATTAGGGATGAGAGGC-3’ (targeted: 243 bp).

### qRT-PCR

Total RNAs of mouse testes were extracted with the RNAprep Pure Tissue Kit (Tiangen, China). RNA was converted into cDNA with FastKing RT Kit (Tiangen). The obtained cDNA was used as templates for the subsequent qRT-PCR with SuperReal PreMix Plus (SYBR Green) (Tiangen) on a CFX Connect RT-PCR Detection System (BioRad, USA). *Actin* was used as an internal control. Primers for *Cfap47*: 5’-GCAGAATACCAGGGTCAGTTACCTA-3’ and 5’-GTTCAACCACACGCACTTTGATG-3’; primers for *Actin*: 5’-GGCTGTATTCCCCTCCATCG-3’ and 5’-CCAGTTGGTAACAATGCCATGT-3’.

### Mouse sperm count, motility, and morphology

Mouse sperm counts were determined using a fertility counting chamber (Makler, Israel). Sperm mobility was assessed via the application of a CASA system (Aidmics Biotechnology, China). The sperm suspension was mounted on a glass slide, air-dried, and fixed with 4% paraformaldehyde for 20 min at room temperature. The slides were stained with Papanicolaou solution (Solarbio, China) and observed using a light microscope (Leica).

### In vitro fertilization

Adult C57BL/6 J female mice were superovulated by injecting 5 IU of pregnant mare serum gonadotropin (PMSG), followed by 5 IU of hCG 48 h later. Sperm capacitation was performed for 1 h using TYH solution. Cumulus-oocyte complexes (COCs) were obtained from the ampulla of the uterine tube at 14 h after hCG injection. COCs were then incubated with ~3 μL sperm suspension (sperm concentration: 1–5 × 10^6^) in human tubal fluid liquid drops at 37 °C under 5% CO_2_. After 6 h, eggs were transferred to liquid drops of KSOM medium. Two-cell embryos were counted at 1 day post fertilization. All reagents were purchased from Aibei Biotechnology.

### Motility of ependymal cilia

Brains were removed, washed in HBSS (Invitrogen) supplemented with 25 mM HEPES (pH 7.4), trimmed for sagittal or coronal sectioning of the third and lateral ventricles, and sectioned into 130-μm slices using a vibratome (Leica Microsystems). Sections were observed with differential interference contrast microscopy using an inverted microscope (Olympus). Videos of ependymal cilia motility were recorded at 135 frames per second with a high-speed camera (Zhongke Junda Vision Technology). The ciliary beat frequency of cilia was measured as described previously.^42^

## Supplementary information

Supplementary information, Figure S1

Supplementary information, Figure S2

Supplementary information, Figure S3

Supplementary information, Figure S4

Supplementary information, Figure S5

Supplementary information, Figure S6

Supplementary information, Figure S7

Supplementary information, Figure S8

Supplementary information, Figure S9

Supplementary information, Figure S10

Supplementary information, Figure S11

Supplementary information, Figure S12

Supplementary information, Figure S13

Supplementary information, Figure S14

Supplementary information, Figure S15

Supplementary information, Figure S16

Supplementary information, Figure S17

Supplementary information, Figure S18

Supplementary information, Figure S19

Supplementary information, Figure S20

Supplementary information, Figure S21

Supplementary information, Figure S22

Supplementary information, Figure S23

Supplementary information, Figure S24

Supplementary information, Figure S25

Supplementary information, Figure S26

Supplementary information, Figure S27

Supplementary information, Figure S28

Supplementary information, Figure S29

Supplementary information, Figure S30

Supplementary information, Figure S31

Supplementary information, Table S1

Supplementary information, Table S2

Supplementary information, Table S3

Supplementary information, Table S4

Supplementary information, Table S5

Supplementary information, Table S6

Supplementary information, Table S7

Supplementary information, Table S8

Supplementary information, Table S9

Supplementary information, Table S10

Supplementary information, Table S11

Supplementary information, Video legend

Supplementary information, Video S1

Supplementary information, Video S2

Supplementary information, Video S3

## Data Availability

The atomic coordinates of the WT mouse sperm CA reported in this work have been deposited in the PDB and are available under accession code 9IJJ. The corresponding map has been deposited in the Electron Microscope Data Bank (EMDB) under accession code EMD-60633. The local refined maps have also been deposited in the EMDB under the accession codes EMD-60428 (C1), EMD-60429 (C1a), EMD-60430 (C1b), EMD-60431 (C2), EMD-60432 (C2-8), EMD-60436 (C2a), EMD-60446 (C2b), EMD-60448 (Cm), EMD-60449 (Cma1), EMD-60454 (Cma2), and EMD-60455 (Cmb). The map of sperm CA from *Cfap47*-KO mice has been deposited in the EMDB under accession code EMD-60597.
