# Supplementary material for: In situ structure of the mouse sperm central apparatus reveals mechanistic insights into asthenozoospermia
Source: Cell Res. 2025 Jun 5;35(8):551–67. doi: 10.1038/s41422-025-01135-2 (PMC12297659; doi:10.1038/s41422-025-01135-2)
Supplement: Supplementary file 1 — Supplementary information, Figure S1 [file 41422_2025_1135_MOESM1_ESM.pdf]

Supplementary information, Figure S1

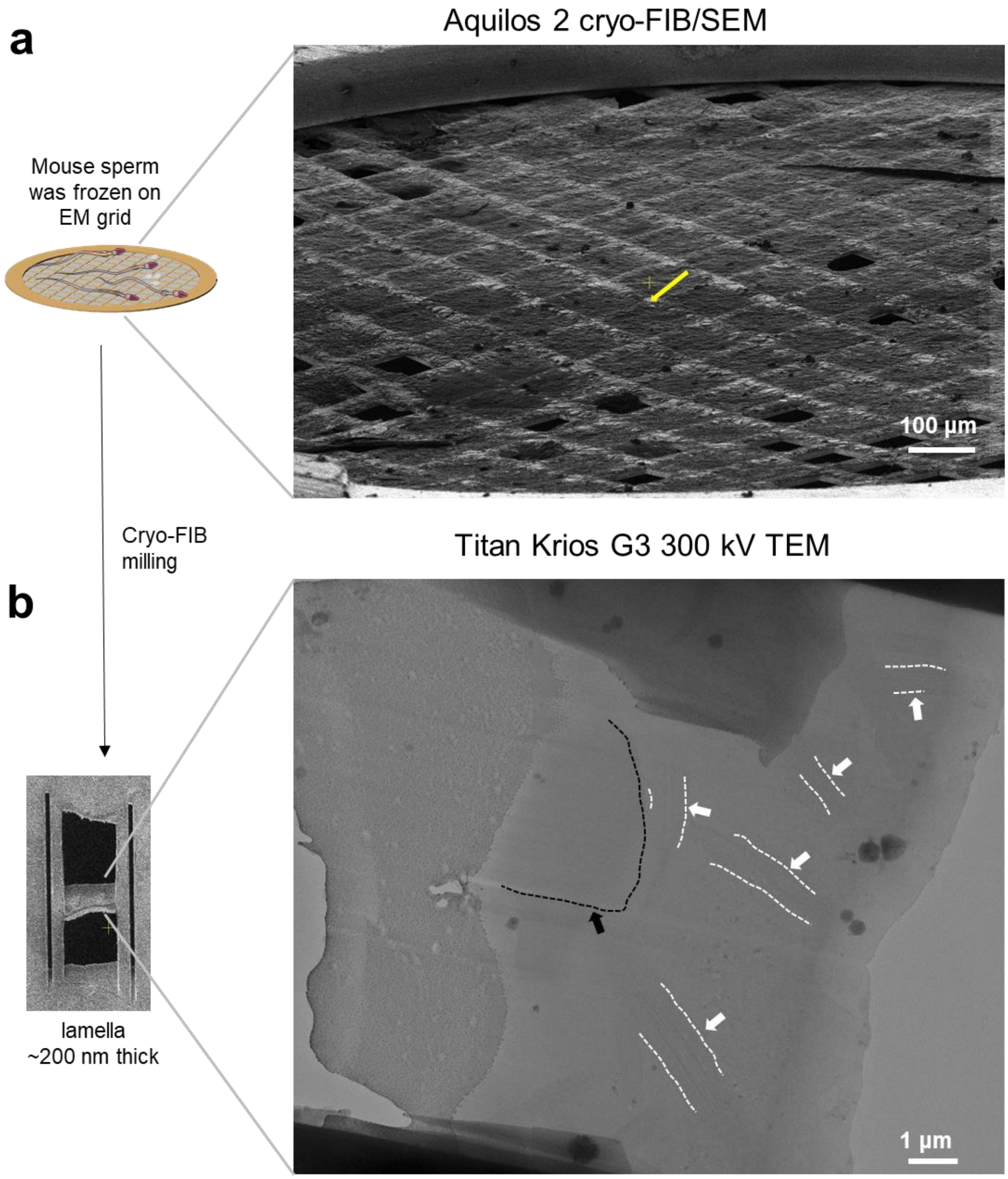

Fig. S1 Cryo-FIB milling of mouse sperm axoneme. a PBS-diluted mouse sperm was plunge-frozen onto an EM grid. The grid was then transferred into an Aquilos 2 cryo-FIB/SEM system, where low magnification SEM imaging (100x) shows densely packed sperm cells on the grid (yellow arrow). **b** A thin cryo-lamella (~200 nm thick) containing the sperm axoneme was generated by cryo-FIB milling. Then the lamella was examined in a Titan Krios G3 300 kV TEM. The white arrow indicates sperm axonemes. The white dashed line marks the outline of the sperm tail, while the black arrow and dashed line mark the sperm head.
