# Supplementary figures and images for: In situ structure of the mouse sperm central apparatus reveals mechanistic insights into asthenozoospermia
Source: Cell Res. 2025 Jun 5;35(8):551–67. doi: 10.1038/s41422-025-01135-2 (PMC12297659; doi:10.1038/s41422-025-01135-2)

## Supplementary information, Figure S2

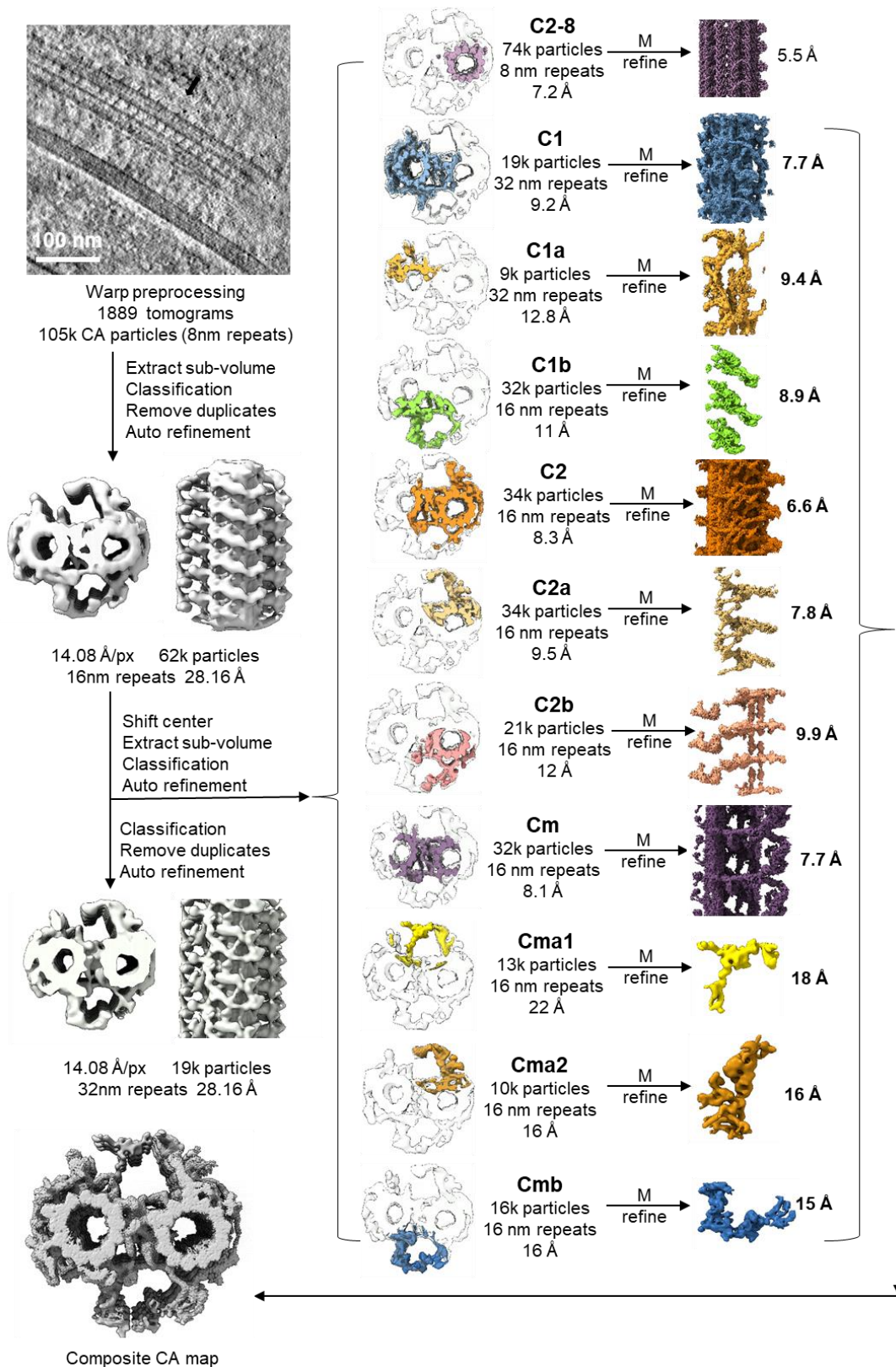

**Fig. S2 Data processing procedure for mouse sperm CA.**

Supplement: Supplementary file 2 — Supplementary information, Figure S2 [file 41422_2025_1135_MOESM2_ESM.pdf]
