# Supplementary material for: In situ structure of the mouse sperm central apparatus reveals mechanistic insights into asthenozoospermia
Source: Cell Res. 2025 Jun 5;35(8):551–67. doi: 10.1038/s41422-025-01135-2 (PMC12297659; doi:10.1038/s41422-025-01135-2)
Supplement: Supplementary file 3 — Supplementary information, Figure S3 [file 41422_2025_1135_MOESM3_ESM.pdf]

Supplementary information, Figure S3

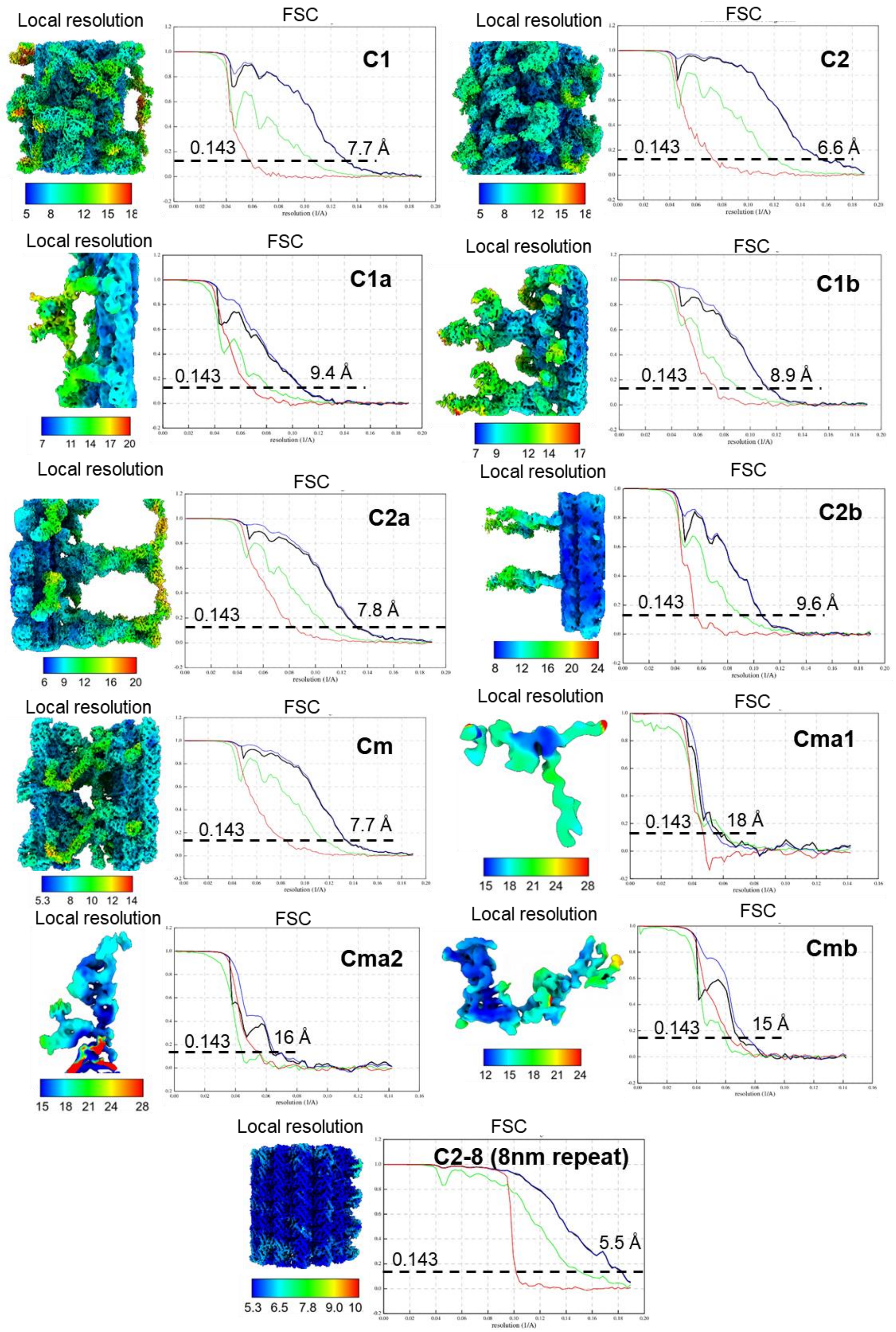

Fig. S3 Local resolution and gold standard Fourier shell correlation (FSC) for all the density maps of mouse sperm CA structure. The resolution at the FSC cut-off criteria of 0.143 are indicated accordingly. The local regions are described in Supplementary information, Fig. S2.
