# Supplementary material for: In situ structure of the mouse sperm central apparatus reveals mechanistic insights into asthenozoospermia
Source: Cell Res. 2025 Jun 5;35(8):551–67. doi: 10.1038/s41422-025-01135-2 (PMC12297659; doi:10.1038/s41422-025-01135-2)
Supplement: Supplementary file 4 — Supplementary information, Figure S4 [file 41422_2025_1135_MOESM4_ESM.pdf]

Supplementary information, Figure S4

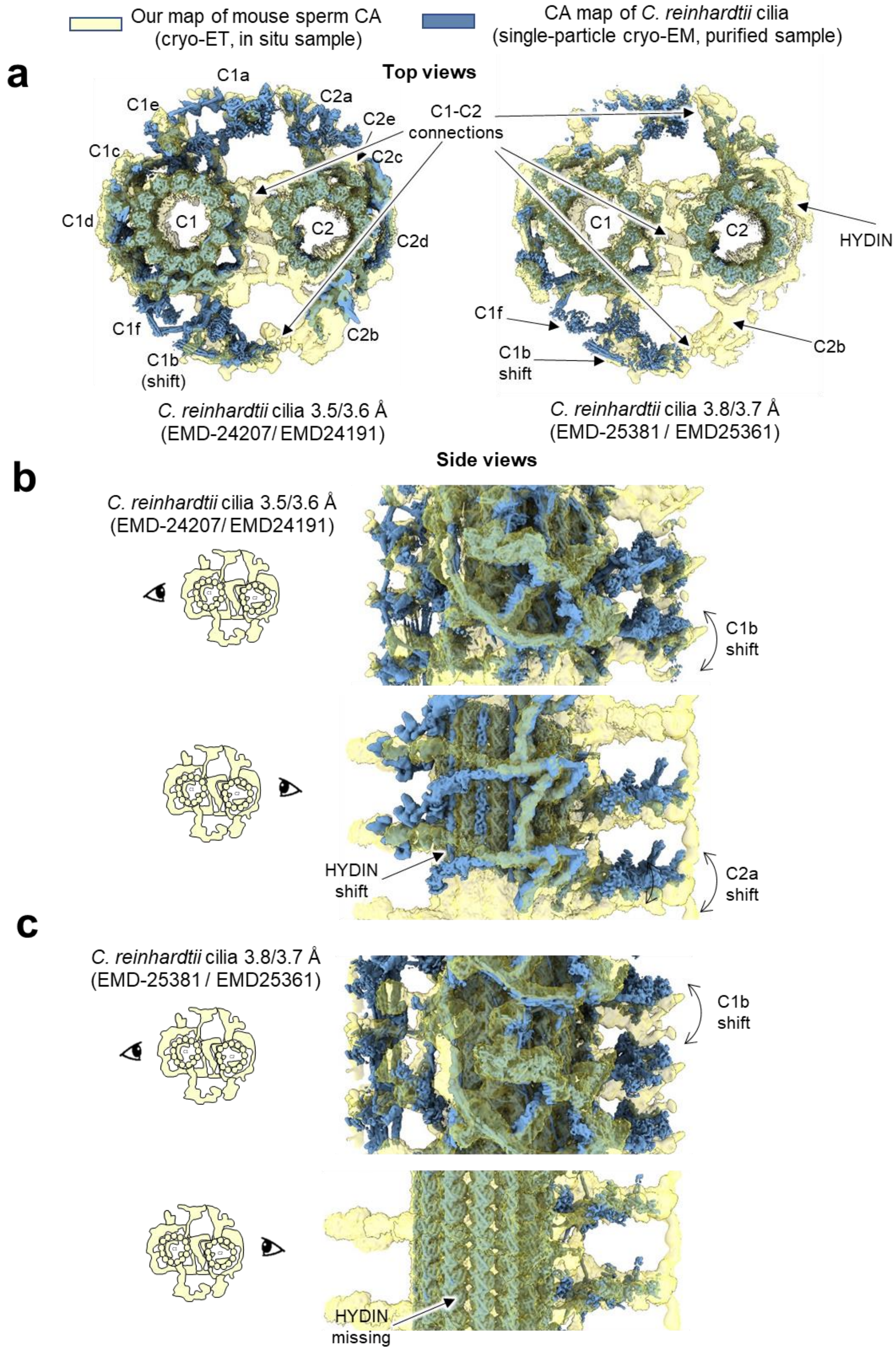

Fig. S4 Comparison of our CA map with that from *C. reinhardtii*. Our map of mouse sperm CA (yellow), derived from in situ cryo-ET, is fitted into two CA maps of *C. reinhardtii* cilia (blue), which were resolved through single-particle cryo-EM of purified samples. Transverse section (**a**) and side (**b** and **c**) views are shown. The projection names and major structural differences have been indicated.
