# Supplementary material for: In situ structure of the mouse sperm central apparatus reveals mechanistic insights into asthenozoospermia
Source: Cell Res. 2025 Jun 5;35(8):551–67. doi: 10.1038/s41422-025-01135-2 (PMC12297659; doi:10.1038/s41422-025-01135-2)
Supplement: Supplementary file 5 — Supplementary information, Figure S5 [file 41422_2025_1135_MOESM5_ESM.pdf]

Supplementary information, Figure S5

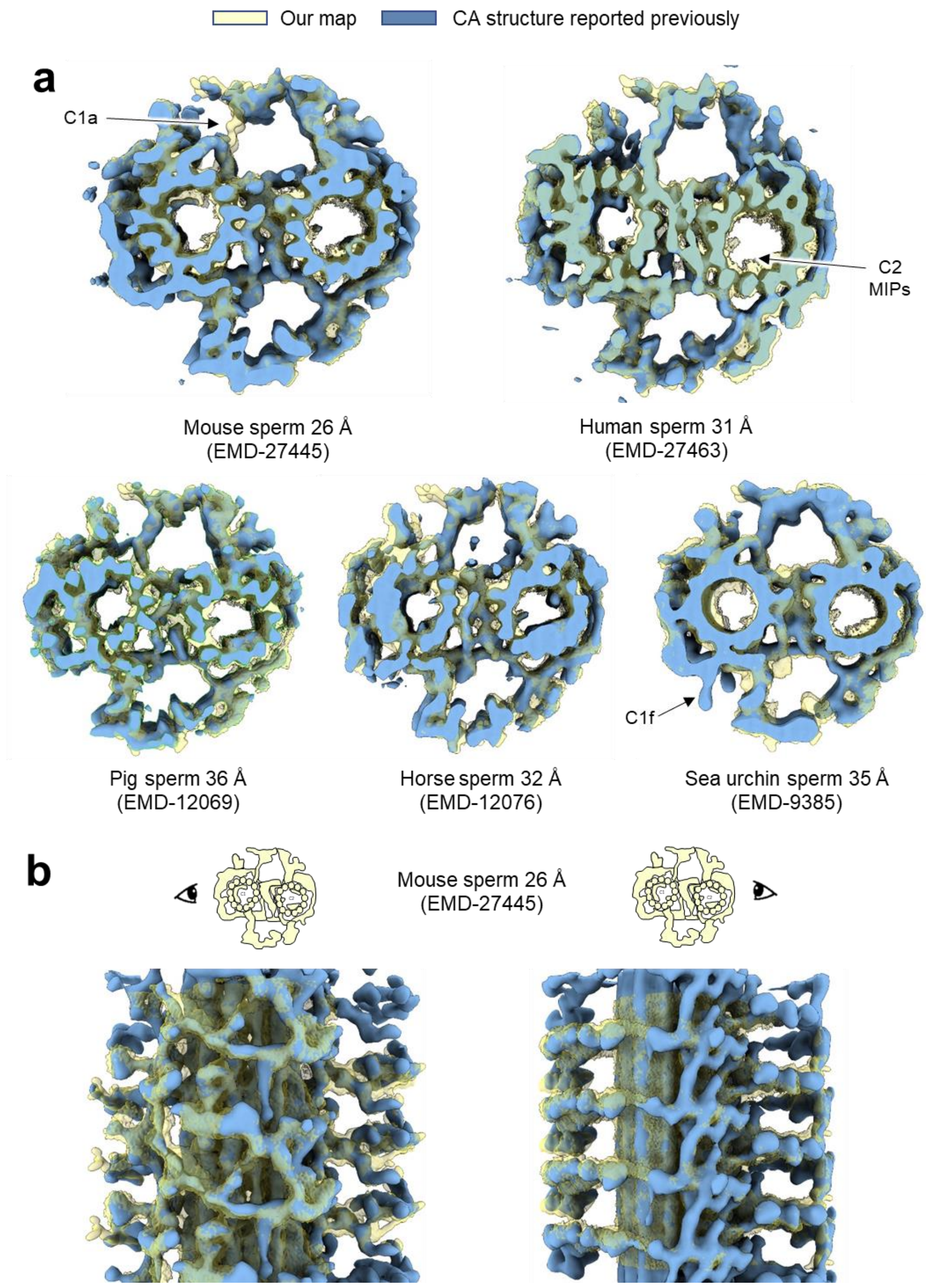

Fig. S5 Comparison of our CA map with other reported CA maps. a Our CA map (yellow) is fitted into other CA maps from five species (blue) and displayed in transverse section view. **b** Our CA map (yellow) is fitted into previously reported low-resolution map of mouse sperm CA (blue) and displayed in two side views.
