# Supplementary material for: In situ structure of the mouse sperm central apparatus reveals mechanistic insights into asthenozoospermia
Source: Cell Res. 2025 Jun 5;35(8):551–67. doi: 10.1038/s41422-025-01135-2 (PMC12297659; doi:10.1038/s41422-025-01135-2)
Supplement: Supplementary file 6 — Supplementary information, Figure S6 [file 41422_2025_1135_MOESM6_ESM.pdf]

Supplementary information, Figure S6

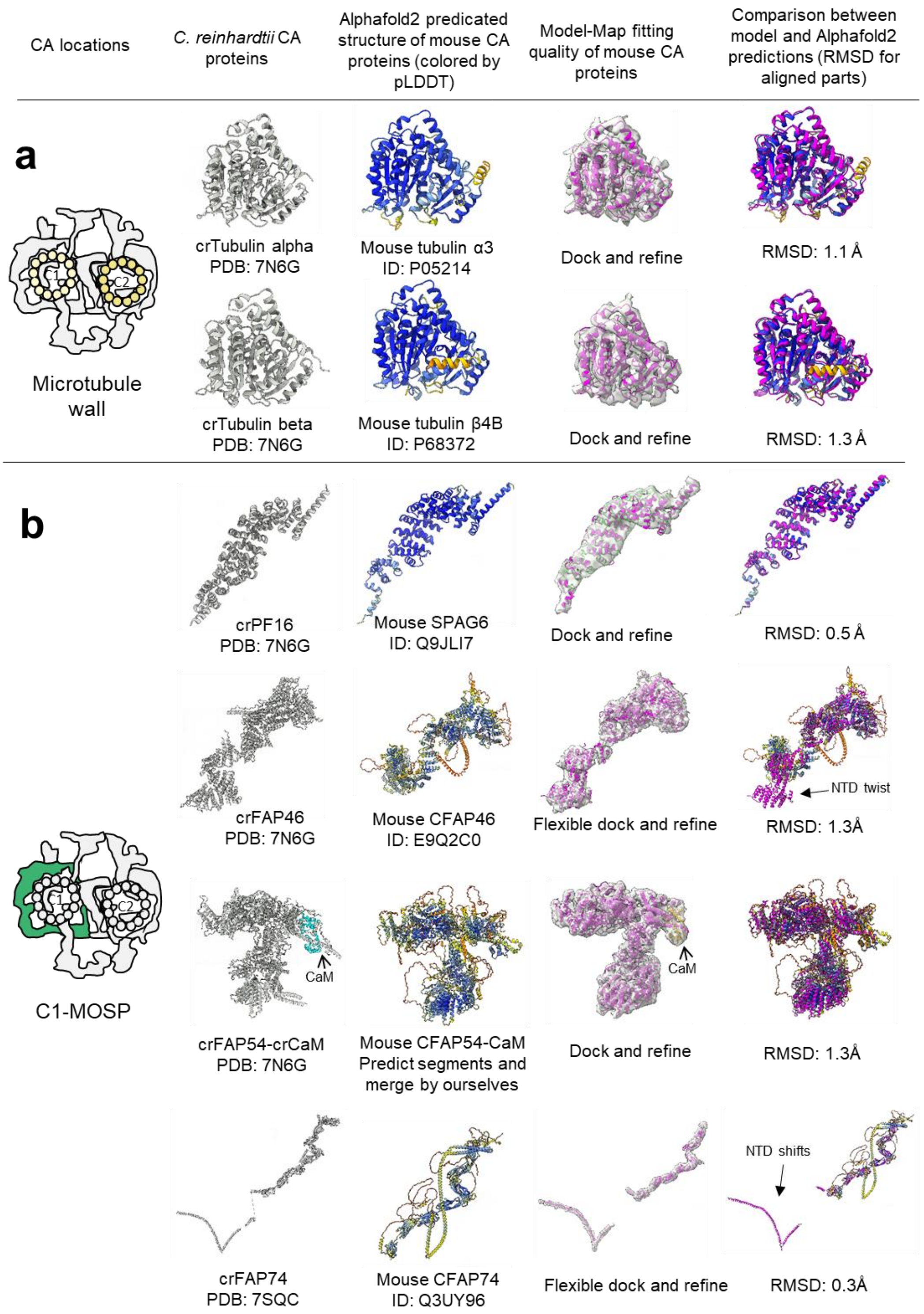

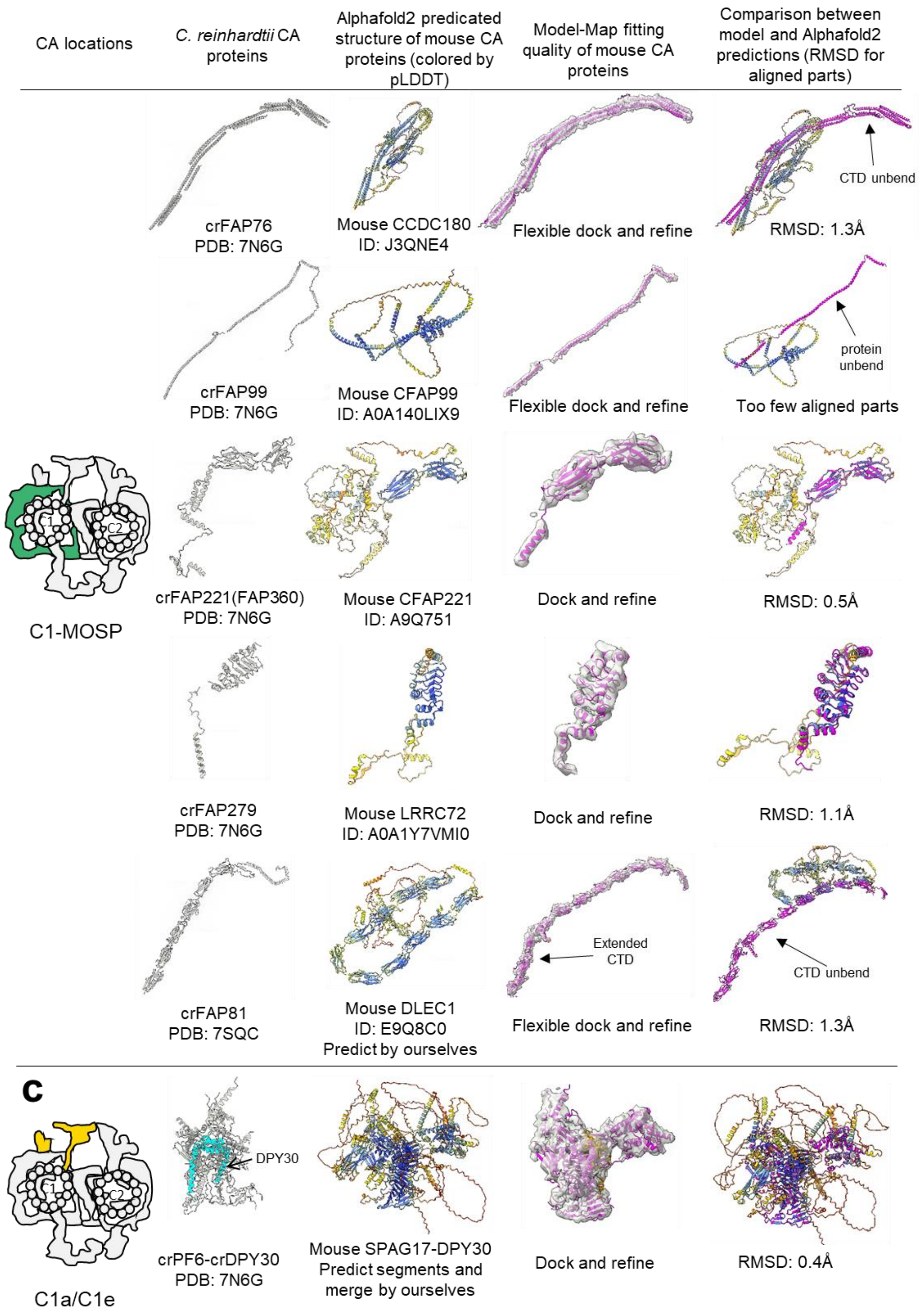
CA locations
C. reinhardtii CA proteins
Alphafold2 predicated structure of mouse CA proteins (colored by pLDDT)
Model-Map fitting quality of mouse CA proteins
Comparison between model and Alphafold2 predictions (RMSD for aligned parts)
crFAP76
PDB: 7N6G
Mouse CCDC180
ID: J3QNE4
Flexible dock and refine
CTD unbend
RMSD: 1.3Å
crFAP99
PDB: 7N6G
Mouse CFAP99
ID: A0A140LIX9
Flexible dock and refine
protein unbend
Too few aligned parts
C1
C2
C1-MOSP
crFAP221(FAP360)
PDB: 7N6G
Mouse CFAP221
ID: A9Q751
Dock and refine
RMSD: 0.5Å
crFAP279
PDB: 7N6G
Mouse LRRC72
ID: A0A1Y7VMI0
Dock and refine
RMSD: 1.1Å
crFAP81
PDB: 7SQC
Mouse DLEC1
ID: E9Q8C0
Predict by ourselves
Extended CTD
Flexible dock and refine
CTD unbend
RMSD: 1.3Å
c
C1
C2
C1a/C1e
DPY30
crPF6-crDPY30
PDB: 7N6G
Mouse SPAG17-DPY30
Predict segments and merge by ourselves
Dock and refine
RMSD: 0.4Å

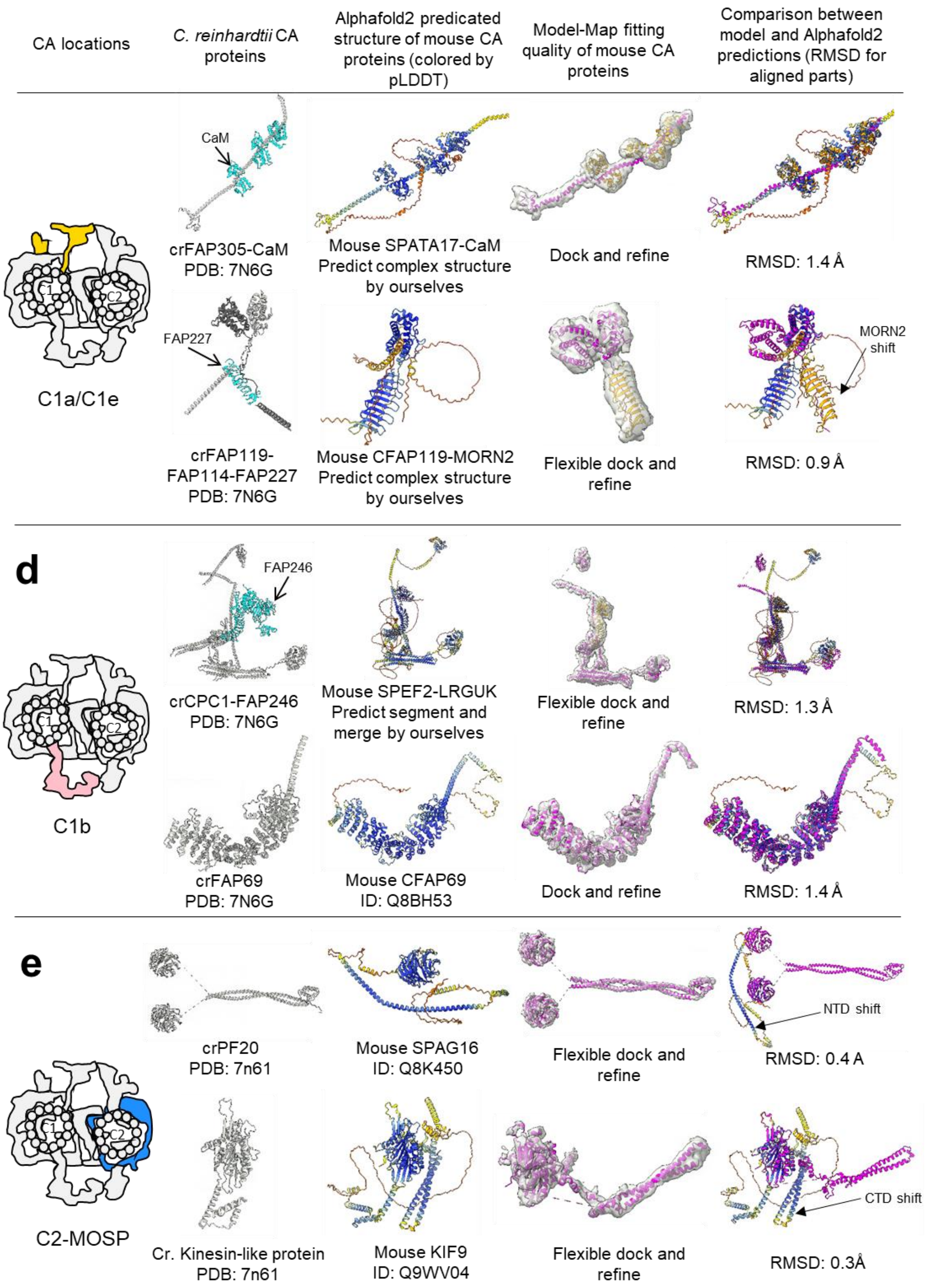

CA locations
C. reinhardtii CA proteins
Alphafold2 predicated structure of mouse CA proteins (colored by pLDDT)
Model-Map fitting quality of mouse CA proteins
Comparison between model and Alphafold2 predictions (RMSD for aligned parts)
CaM
crFAP305-CaM
PDB: 7N6G
Mouse SPATA17-CaM
Predict complex structure by ourselves
Dock and refine
RMSD: 1.4 Å
C1
C2
C1a/C1e
FAP227
MORN2 shift
crFAP119-FAP114-FAP227
PDB: 7N6G
Mouse CFAP119-MORN2
Predict complex structure by ourselves
Flexible dock and refine
RMSD: 0.9 Å
d
FAP246
crCPC1-FAP246
PDB: 7N6G
Mouse SPEF2-LRGUK
Predict segment and merge by ourselves
Flexible dock and refine
RMSD: 1.3 Å
C1b
crFAP69
PDB: 7N6G
Mouse CFAP69
ID: Q8BH53
Dock and refine
RMSD: 1.4 Å
e
crPF20
PDB: 7n61
Mouse SPAG16
ID: Q8K450
Flexible dock and refine
NTD shift
RMSD: 0.4 A
C2-MOSP
Cr. Kinesin-like protein
PDB: 7n61
Mouse KIF9
ID: Q9WV04
Flexible dock and refine
CTD shift
RMSD: 0.3Å

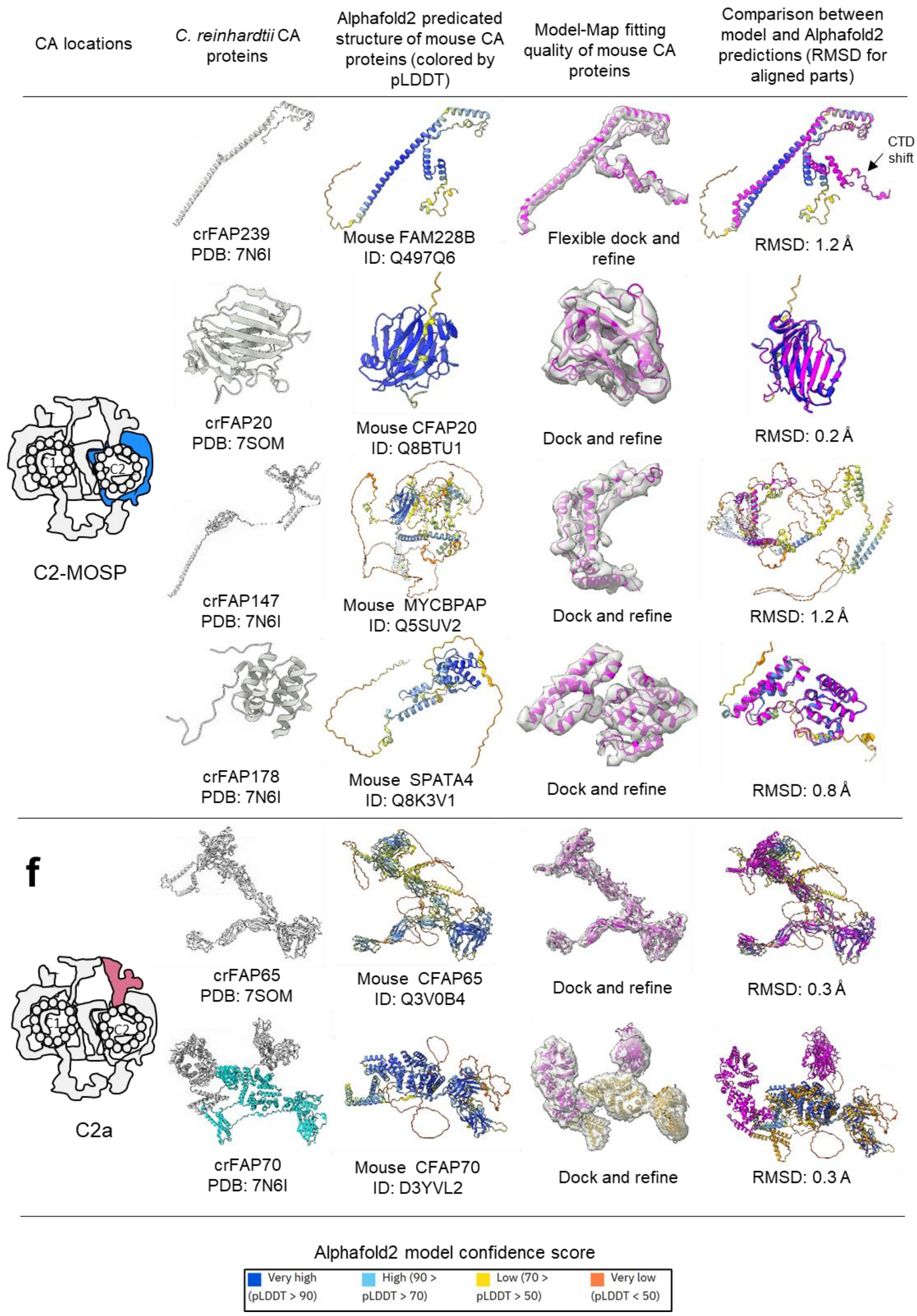

Fig. S6 The model building details of CA components that have homologs in *C. reinhardtii* CA structure. For each component, it shows the protein location in CA, the model of homologous protein in *C. reinhardtii* CA structure (cr for short), the Alphafold2 predicted structure (colored by pLDDT score), the model-map fitting quality of our refined model, and the difference between the Alphafold2 predicted structure and our refined model. Root-mean-square deviation (RMSD) are calculated

using Matchmaker tool in ChimeraX, only taking into account the aligned pairs. Protein components are grouped according to their positions in the CA structure, including microtubule wall (**a**), C1-MOSP (**b**), C1a/C1e projections (**c**), C1b projection (**d**), C2-MOSP (**e**), C2a projection (**f**).
