# Supplementary material for: In situ structure of the mouse sperm central apparatus reveals mechanistic insights into asthenozoospermia
Source: Cell Res. 2025 Jun 5;35(8):551–67. doi: 10.1038/s41422-025-01135-2 (PMC12297659; doi:10.1038/s41422-025-01135-2)
Supplement: Supplementary file 8 — Supplementary information, Figure S8 [file 41422_2025_1135_MOESM8_ESM.pdf]

Supplementary information, Figure S8

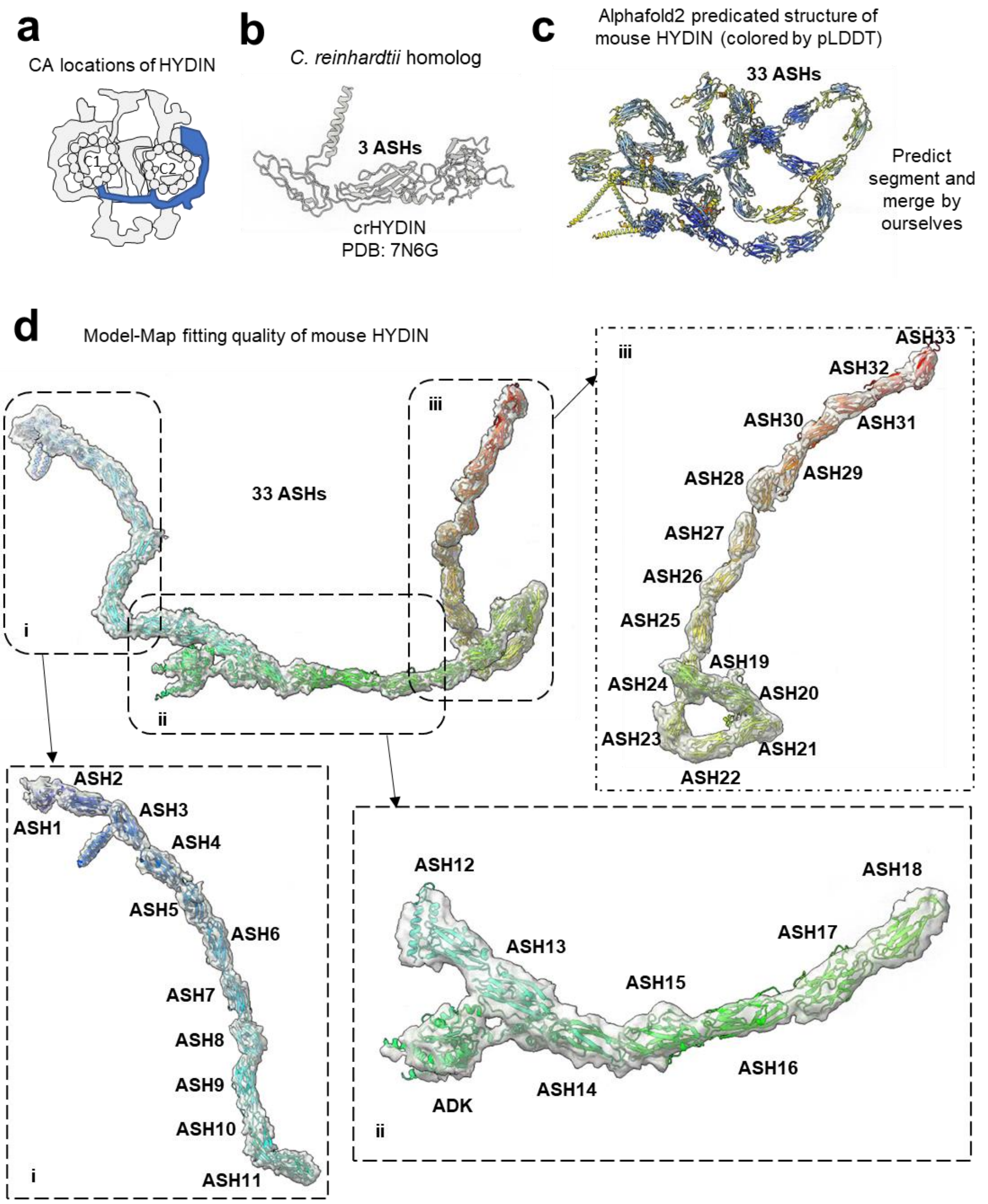

Fig. S8 Model building details of HYDIN. a HYDIN location in mouse sperm CA. **b** The structure model of HYDIN's homologous protein in *C. reinhardtii* (cr for short). **c** Alphafold predicted structure of full-length mouse HYDIN protein, colored by pLDDT score. Due to the sequence length limitation for direct prediction, we predicted several fragments separately and merged them. **d** Model-map fitting quality of the HYDIN model in our CA structure, including overall view and three zoomed-in views.
