# Supplementary material for: In situ structure of the mouse sperm central apparatus reveals mechanistic insights into asthenozoospermia
Source: Cell Res. 2025 Jun 5;35(8):551–67. doi: 10.1038/s41422-025-01135-2 (PMC12297659; doi:10.1038/s41422-025-01135-2)
Supplement: Supplementary file 9 — Supplementary information, Figure S9 [file 41422_2025_1135_MOESM9_ESM.pdf]

**Supplementary information, Figure S9**

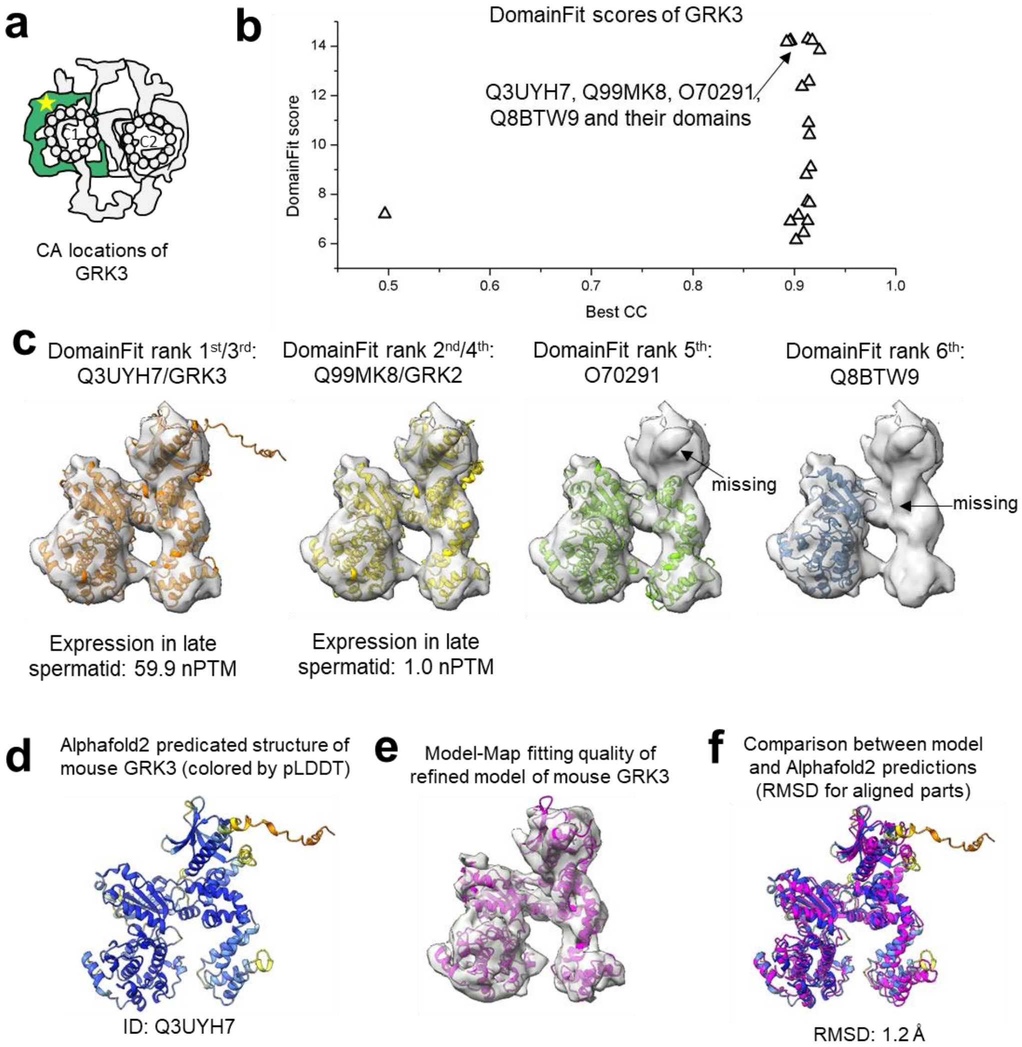


**Fig. S9 Details of GRK3 identification and model building. a** Localization of GRK3 in mouse sperm CA (yellow star). **b** The DomainFit score plot for GRK3 density. Reported proteomes of mouse sperm[18] are used as the search candidates. UniProt IDs of top hits are labeled. The x-axis represents the best cross-correlation (CC) of model-map fitting. **c** Model-map fitting quality for the top hits. For proteins with similar fitting quality, their expression levels in late spermatids are indicated according to The Human Protein Atlas database. GRK3 exhibits the highest fitting quality and is highly expressed in sperm. **d** The Alphafold2 predicted structure of GRK3, colored by pLDDT score. **e** Model-map fitting quality of refined GRK3 model (magentas) within our CA structure. **f** Structural comparison between the Alphafold2 predicted model (pLDDT coloring) and the refined GRK3 model (magentas). RMSD values were calculated using the Matchmaker tool in ChimeraX, considering only aligned atom pairs.
