# Supplementary material for: In situ structure of the mouse sperm central apparatus reveals mechanistic insights into asthenozoospermia
Source: Cell Res. 2025 Jun 5;35(8):551–67. doi: 10.1038/s41422-025-01135-2 (PMC12297659; doi:10.1038/s41422-025-01135-2)
Supplement: Supplementary file 10 — Supplementary information, Figure S10 [file 41422_2025_1135_MOESM10_ESM.pdf]

Supplementary information, Figure S10

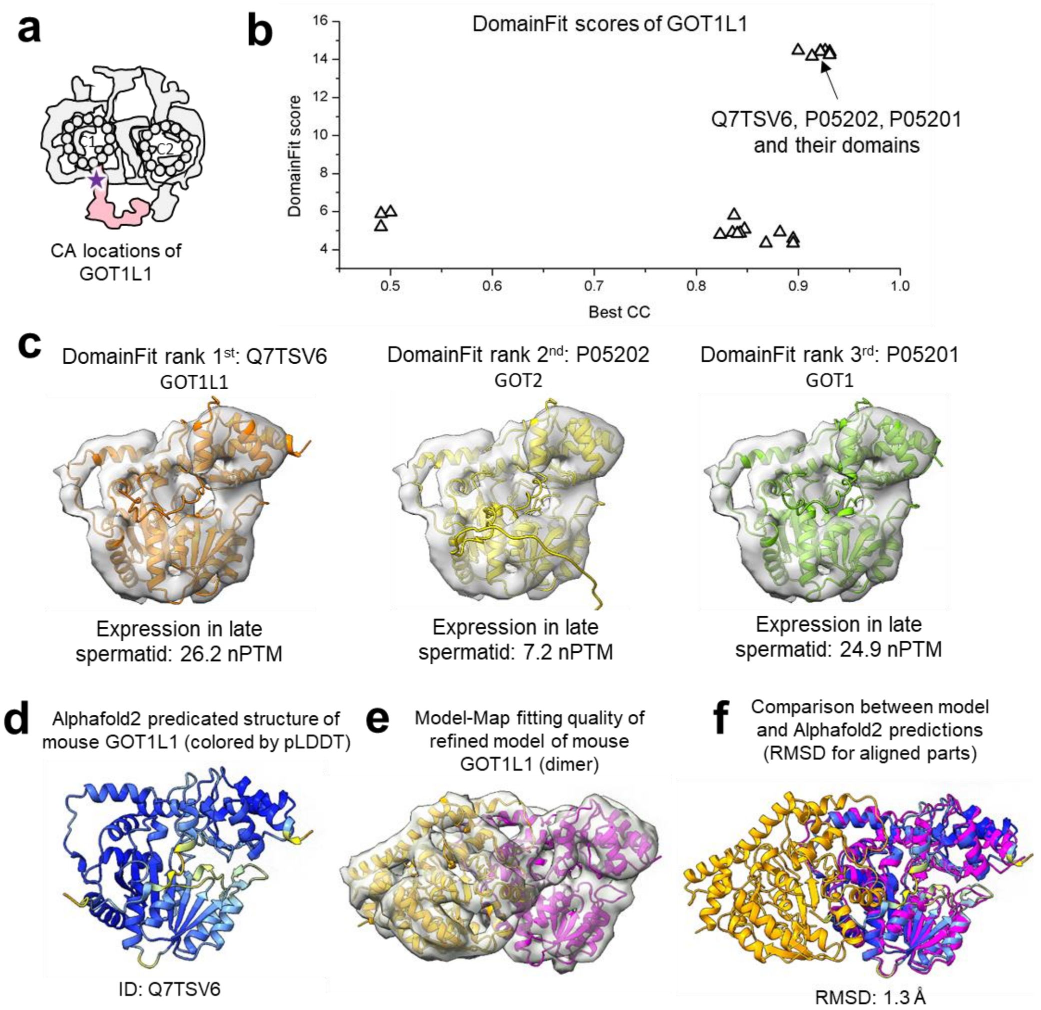

Fig. S10 Details of GOT1L1 identification and model building. a Localization of GOT1L1 in mouse sperm CA (magentas star). **b** The DomainFit score plot for GOT1L1 density. Reported proteomes of mouse sperm[18] are used as the search candidates. UniProt IDs of top hits are labeled. The x-axis represents the best cross-correlation (CC) of model-map fitting. **c** Model-map fitting quality for the top hits. For proteins with similar fitting quality, their expression levels in late spermatids are indicated according to The Human Protein Atlas database. GOT1L1 exhibits the highest fitting quality and is more highly expressed in sperm. **d** The Alphafold2 predicted structure of GOT1L1, colored by pLDDT score. **e** Model-map fitting quality of refined GOT1L1 dimeric model (magentas and yellow) within our CA structure. **f** Structural comparison between the Alphafold2 predicted model (pLDDT coloring) and the refined GOT1L1 model (magentas and yellow). RMSD values were calculated using the Matchmaker tool in ChimeraX, considering only aligned atom pairs.
