# Supplementary material for: In situ structure of the mouse sperm central apparatus reveals mechanistic insights into asthenozoospermia
Source: Cell Res. 2025 Jun 5;35(8):551–67. doi: 10.1038/s41422-025-01135-2 (PMC12297659; doi:10.1038/s41422-025-01135-2)
Supplement: Supplementary file 11 — Supplementary information, Figure S11 [file 41422_2025_1135_MOESM11_ESM.pdf]

Supplementary information, Figure S11

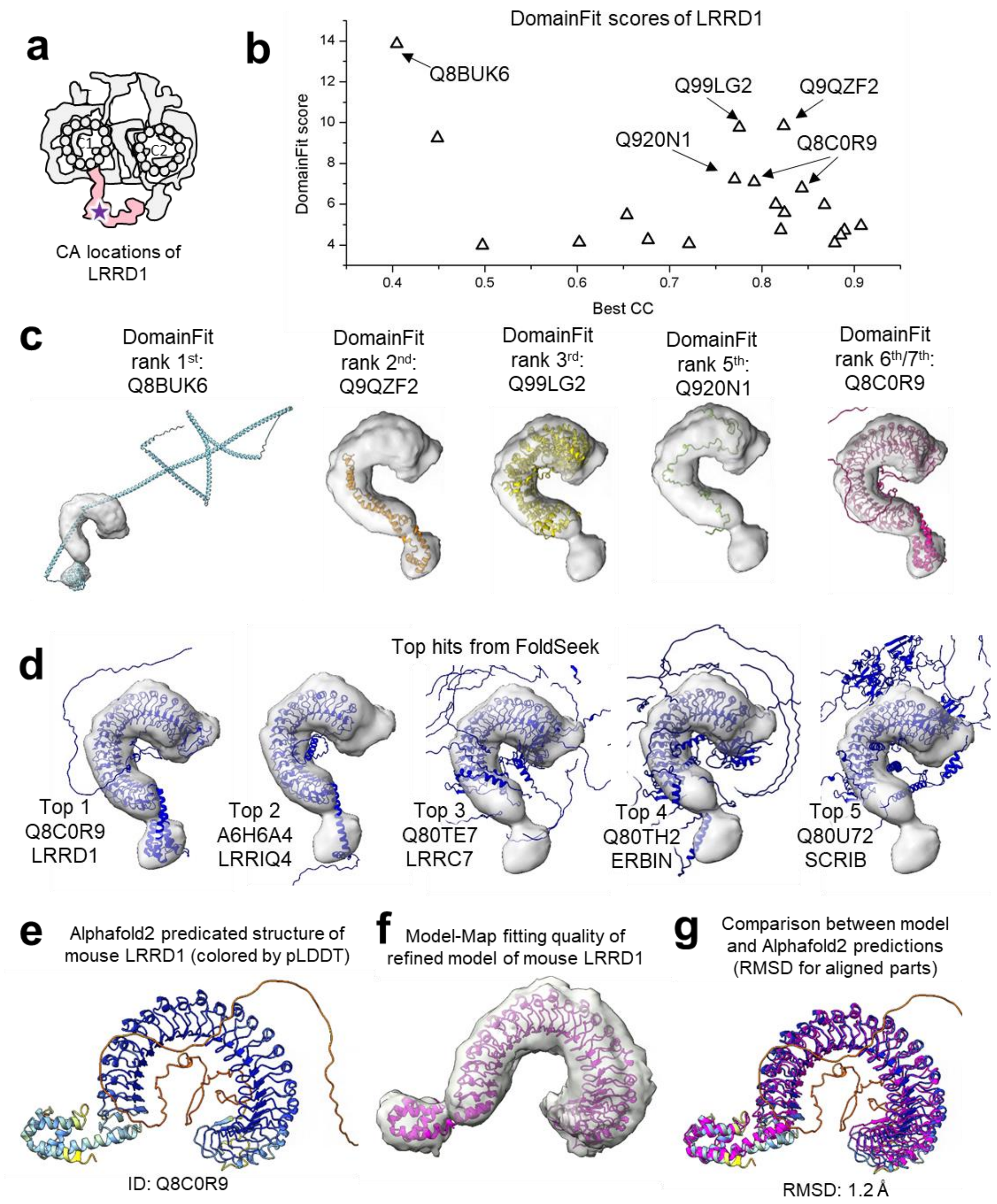

Fig. S11 Details of LRRD1 identification and model building. a Localization of LRRD1 in mouse sperm CA (magentas star). **b** The DomainFit score plot for LRRD1 density. Reported proteomes of mouse sperm[18] are used as the search candidates. UniProt IDs of top hits are labeled. The x-axis represents the best cross-correlation (CC) of model-map fitting. **c** Model-map fitting quality for the top hits. LRRD1 exhibits the highest fitting quality. **d** To find alternatives with similar folds, the structure of LRRD1 was used as inputs to FoldSeek to query the AlphaFold database of mouse proteome. No hit matches the density as well as LRRD1. **e** The Alphafold2 predicted structure of LRRD1, colored by pLDDT score. **f** Model-map fitting quality of refined LRRD1 model (magentas) within our CA structure. **g** Structural comparison between the

Alphafold2 predicted model (pLDDT coloring) and the refined LRRD1 model (magentas). RMSD values were calculated using the Matchmaker tool in ChimeraX, considering only aligned atom pairs.
