# Supplementary material for: In situ structure of the mouse sperm central apparatus reveals mechanistic insights into asthenozoospermia
Source: Cell Res. 2025 Jun 5;35(8):551–67. doi: 10.1038/s41422-025-01135-2 (PMC12297659; doi:10.1038/s41422-025-01135-2)
Supplement: Supplementary file 14 — Supplementary information, Figure S14 [file 41422_2025_1135_MOESM14_ESM.pdf]

Supplementary information, Figure S14

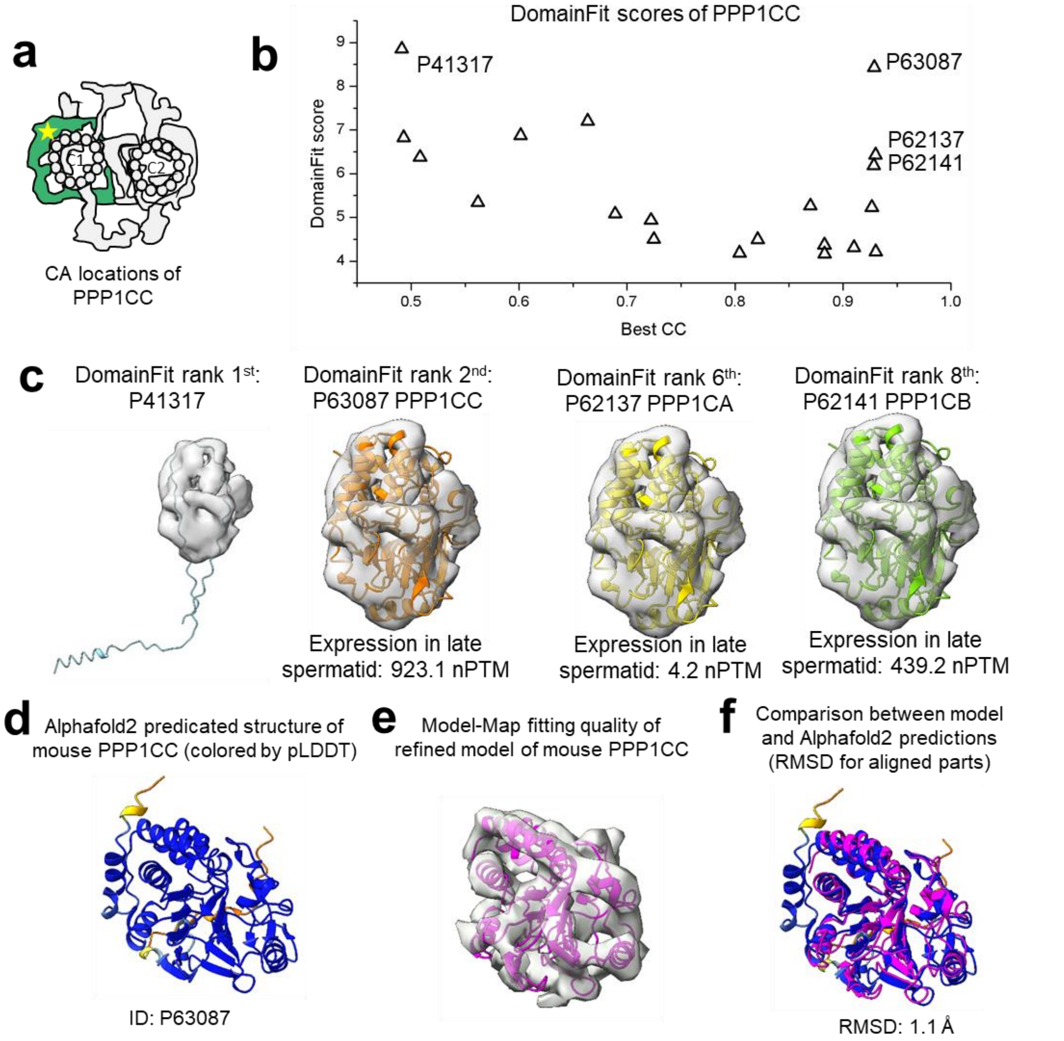

Fig. S14 Details of PPP1CC identification and model building. a Localization of PPP1CC in mouse sperm CA (yellow star). **b** The DomainFit score plot for PPP1CC density. Reported proteomes of mouse sperm[18] are used as the search candidates. UniProt IDs of top hits are labeled. The x-axis represents the best cross-correlation (CC) of model-map fitting. **c** Model-map fitting quality for the top hits. For proteins with similar fitting quality, their expression levels in late spermatids are indicated according to The Human Protein Atlas database. PPP1CC exhibits the highest fitting quality and is more highly expressed in sperm. **d** The Alphafold2 predicted structure of PPP1CC, colored by pLDDT score. **e** Model-map fitting quality of refined PPP1CC model (magentas) within our CA structure. **f** Structural comparison between the Alphafold2 predicted model (pLDDT coloring) and the refined PPP1CC model (magentas). RMSD values were calculated using the Matchmaker tool in ChimeraX, considering only aligned atom pairs.
