# Supplementary material for: In situ structure of the mouse sperm central apparatus reveals mechanistic insights into asthenozoospermia
Source: Cell Res. 2025 Jun 5;35(8):551–67. doi: 10.1038/s41422-025-01135-2 (PMC12297659; doi:10.1038/s41422-025-01135-2)
Supplement: Supplementary file 15 — Supplementary information, Figure S15 [file 41422_2025_1135_MOESM15_ESM.pdf]

Supplementary information, Figure S15

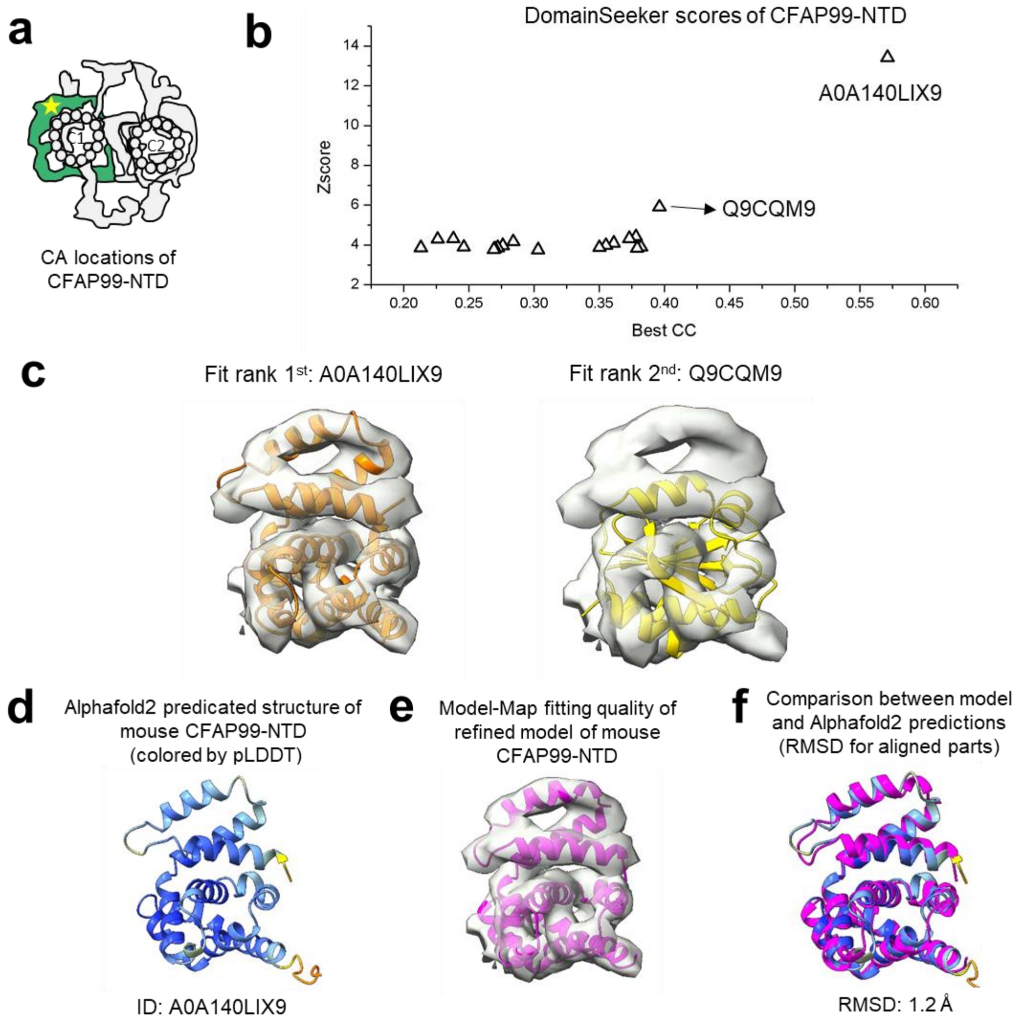

Fig. S15 Details of CFAP99-NTD identification and model building. a Localization of CFAP99-NTD in mouse sperm CA (yellow star). **b** The DomainSeeker score plot for CFAP99-NTD density. The entire mouse proteomes in UniProt database are used as the search candidates. UniProt IDs of top hits are labeled. The x-axis represents the best cross-correlation (CC) of model-map fitting. **c** Model-map fitting quality for the top hits. CFAP99-NTD exhibits the highest fitting quality. **d** The Alphafold2 predicted structure of CFAP99-NTD, colored by pLDDT score. **e** Model-map fitting quality of refined CFAP99-NTD model (magentas) within our CA structure. **f** Structural comparison between the Alphafold2 predicted model (pLDDT coloring) and the refined CFAP99-NTD model (magentas). RMSD values were calculated using the Matchmaker tool in ChimeraX, considering only aligned atom pairs.
