# Supplementary material for: In situ structure of the mouse sperm central apparatus reveals mechanistic insights into asthenozoospermia
Source: Cell Res. 2025 Jun 5;35(8):551–67. doi: 10.1038/s41422-025-01135-2 (PMC12297659; doi:10.1038/s41422-025-01135-2)
Supplement: Supplementary file 18 — Supplementary information, Figure S18 [file 41422_2025_1135_MOESM18_ESM.pdf]

Supplementary information, Figure S18

a

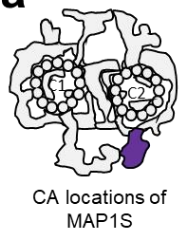

b

crFAP312 (UniParc ID: UPI00015F657D) blast against mouse proteomes

Rank	Uniprot ID	Name	Sequence Length	Blast score
1	**Q8C052**	**MAP1S**	973	99
2	Q99KG7	HPS4	671	81
3 / 4	Q9DAQ4-3<br>Q9DAQ4	CB081	506/598	80
5	Q9DD19	RASF7	359	79

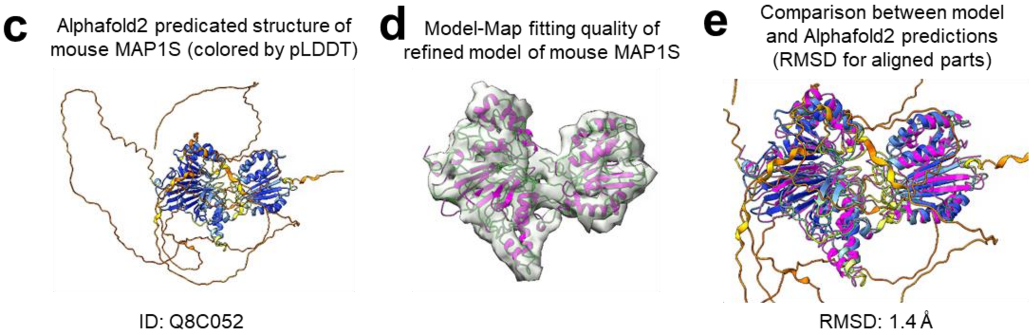

Fig. S18 Details of MAP1S identification and model building. a Localization of MAP1S in mouse sperm CA. **b** FAP312 was reported to be a C2 component in CA.[73] In the blast result of FAP312 in *C. reinhardtii* (crFAP312) against mouse proteomes in UniProt database, MAPS1S showed the highest score. **c** The Alphafold2 predicted structure of MAP1S, colored by pLDDT score. **d** Model-map fitting quality of refined MAP1S model (magentas) within our CA structure. **e** Structural comparison between the Alphafold2 predicted model (pLDDT coloring) and the refined MAP1S model (magentas). RMSD values were calculated using the Matchmaker tool in ChimeraX, considering only aligned atom pairs.
