# Supplementary material for: In situ structure of the mouse sperm central apparatus reveals mechanistic insights into asthenozoospermia
Source: Cell Res. 2025 Jun 5;35(8):551–67. doi: 10.1038/s41422-025-01135-2 (PMC12297659; doi:10.1038/s41422-025-01135-2)
Supplement: Supplementary file 19 — Supplementary information, Figure S19 [file 41422_2025_1135_MOESM19_ESM.pdf]

Supplementary information, Figure S19

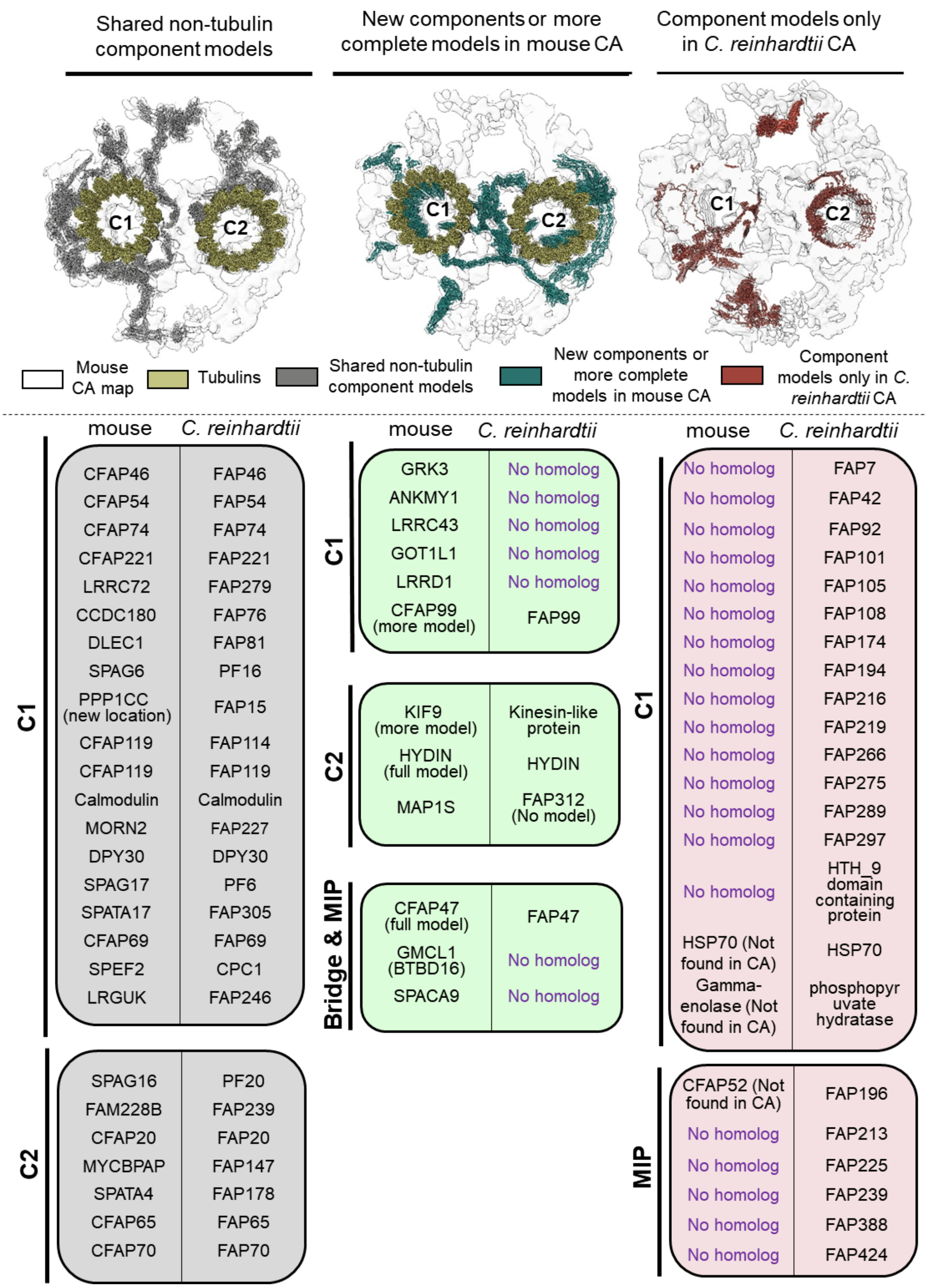

Fig. S19 Comparison of the CA structure of mouse sperm with that of *C. reinhardtii* cilia. For a parallel comparison, the CA models of *C. reinhardtii* (PDB entry 7N6G, 7N61, 7SQC and 7SOM) were also fitted into the mouse CA map for display. Supplementary information Table S3 provides a detailed analysis of protein homologs.
