# Supplementary material for: In situ structure of the mouse sperm central apparatus reveals mechanistic insights into asthenozoospermia
Source: Cell Res. 2025 Jun 5;35(8):551–67. doi: 10.1038/s41422-025-01135-2 (PMC12297659; doi:10.1038/s41422-025-01135-2)
Supplement: Supplementary file 20 — Supplementary information, Figure S20 [file 41422_2025_1135_MOESM20_ESM.pdf]

Supplementary information, Figure S20

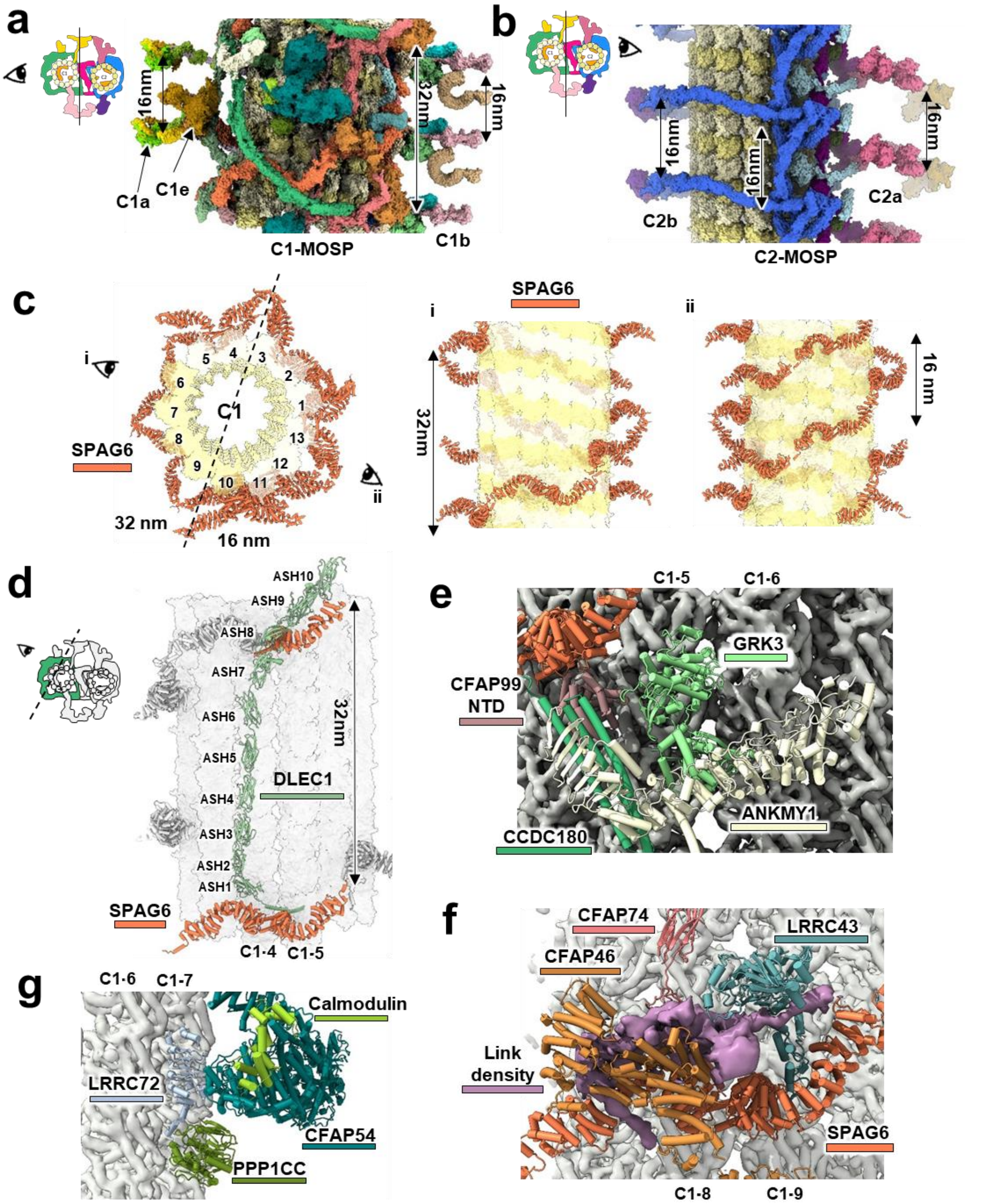

Fig. S20 Detailed structural composition of MOSPs in C1 and C2. a The side view of C1 half of the CA, displaying both 16 nm and 32 nm repeats. **b** The side view of C2 half of the CA, displaying predominant 16 nm repeat. **c** SPAG6 is identified as the primary component of C1-MOSP, forming 16 nm and 32 nm repeats around the C1 microtubule. This is displayed in one cross-sectional view and two side views. **d** The ASH protein DLEC1 interacts with two SPAG6 proteins via its NTD and CTD, establishing the 32 nm periodicity. **e** The assembly of CFAP99, GRK3, ANKMY1, and CCDC180 in C1-MOSP. **f** The assembly of CFAP46, CFAP77, LRRC43, and SPAG6

in C1-MOSP, along with an undefined link density (pink). **g** The arrangement of CFAP54, LRRC72, calmodulin, and PPP1CC in C1-MOSP.
