# Supplementary material for: In situ structure of the mouse sperm central apparatus reveals mechanistic insights into asthenozoospermia
Source: Cell Res. 2025 Jun 5;35(8):551–67. doi: 10.1038/s41422-025-01135-2 (PMC12297659; doi:10.1038/s41422-025-01135-2)
Supplement: Supplementary file 21 — Supplementary information, Figure S21 [file 41422_2025_1135_MOESM21_ESM.pdf]

Supplementary information, Figure S21

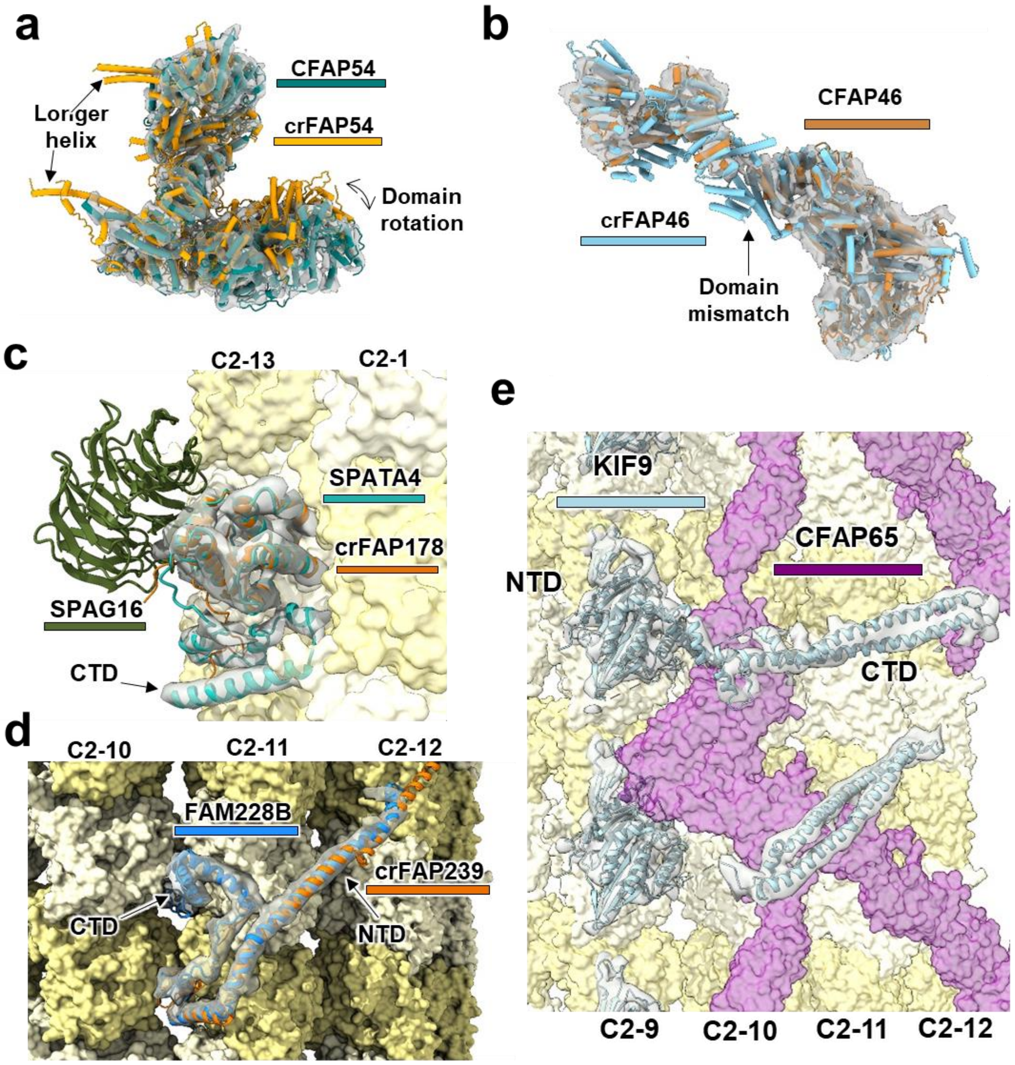

Fig. S21 Structural details of some C1-MOSP and C2-MOSP components. a Structural differences between CFAP54 in our model and its homologous protein FAP54 in *C. reinhardtii* CA (crFAP54) (PDB entry 7N6G). **b** Structural differences between CFAP46 in our model and its homologous protein FAP46 in *C. reinhardtii* CA (crFAP46) (PDB entry 7N6G). **c** Structural differences between SPATA4 in our model and its homologous protein FAP178 in *C. reinhardtii* CA (crFAP178) (PDB entry 7N61). **d** Structural differences between FAP228B in our model and its homologous protein FAP239 in *C. reinhardtii* CA (crFAP239) (PDB entry 7N6I). **e** Location and structure of the KIF9 NTD and CTD.
