# Supplementary material for: In situ structure of the mouse sperm central apparatus reveals mechanistic insights into asthenozoospermia
Source: Cell Res. 2025 Jun 5;35(8):551–67. doi: 10.1038/s41422-025-01135-2 (PMC12297659; doi:10.1038/s41422-025-01135-2)
Supplement: Supplementary file 22 — Supplementary information, Figure S22 [file 41422_2025_1135_MOESM22_ESM.pdf]

Supplementary information, Figure S22

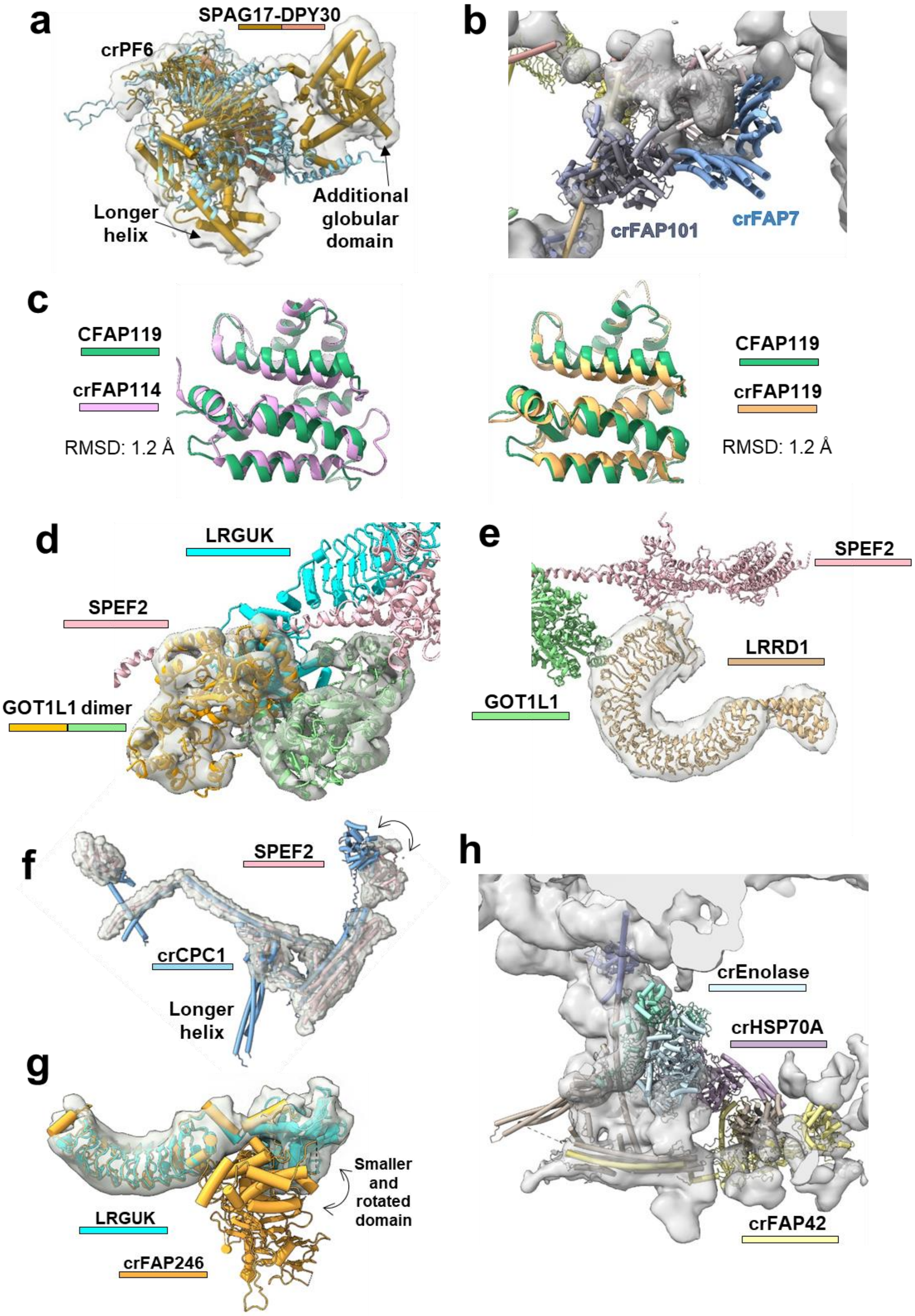

Fig. S22 Structural details of some components in C1 projections. a Structural differences between SPAG17 in our model and its homologous protein PF6 in *C. reinhardtii* CA (crPF6) (PDB entry 7N6G). **b** Two C1a components present in *C. reinhardtii* CA (PDB entry 7N6G), FAP7 (crFAP7) and FAP101 (crFAP101), are absent in mouse sperm CA. **c** Structure comparison between CFAP119 in our model

(green) crFAP114 and (light pink) or crFAP119 (light orange) in *C. reinhardtii* CA (PDB entry 7N6G). RMSD values were calculated using the Matchmaker tool in ChimeraX, considering only aligned atom pairs. **d** Location and structure of the newly identified GOT1L1 dimer in the C1b projection. **e** Location and structure of the newly identified LRRD1 in the C1b projection. **f** Structural differences between SPEF2 in our model and its homologous protein CPC1 in *C. reinhardtii* CA (crCPC1) (PDB entry 7N6G). **g** Structural differences between LRGUK in our model and its homologous protein FAP246 in *C. reinhardtii* CA (crFAP246) (PDB entry 7N6G). **h** Three C1b components present in *C. reinhardtii* CA (PDB entry 7N6G), Enolase (crEnolase), HSP70A (crHSP70A) and FAP42 (crFAP42), are absent in mouse sperm CA.
