# Supplementary material for: In situ structure of the mouse sperm central apparatus reveals mechanistic insights into asthenozoospermia
Source: Cell Res. 2025 Jun 5;35(8):551–67. doi: 10.1038/s41422-025-01135-2 (PMC12297659; doi:10.1038/s41422-025-01135-2)
Supplement: Supplementary file 23 — Supplementary information, Figure S23 [file 41422_2025_1135_MOESM23_ESM.pdf]

Supplementary information, Figure S23

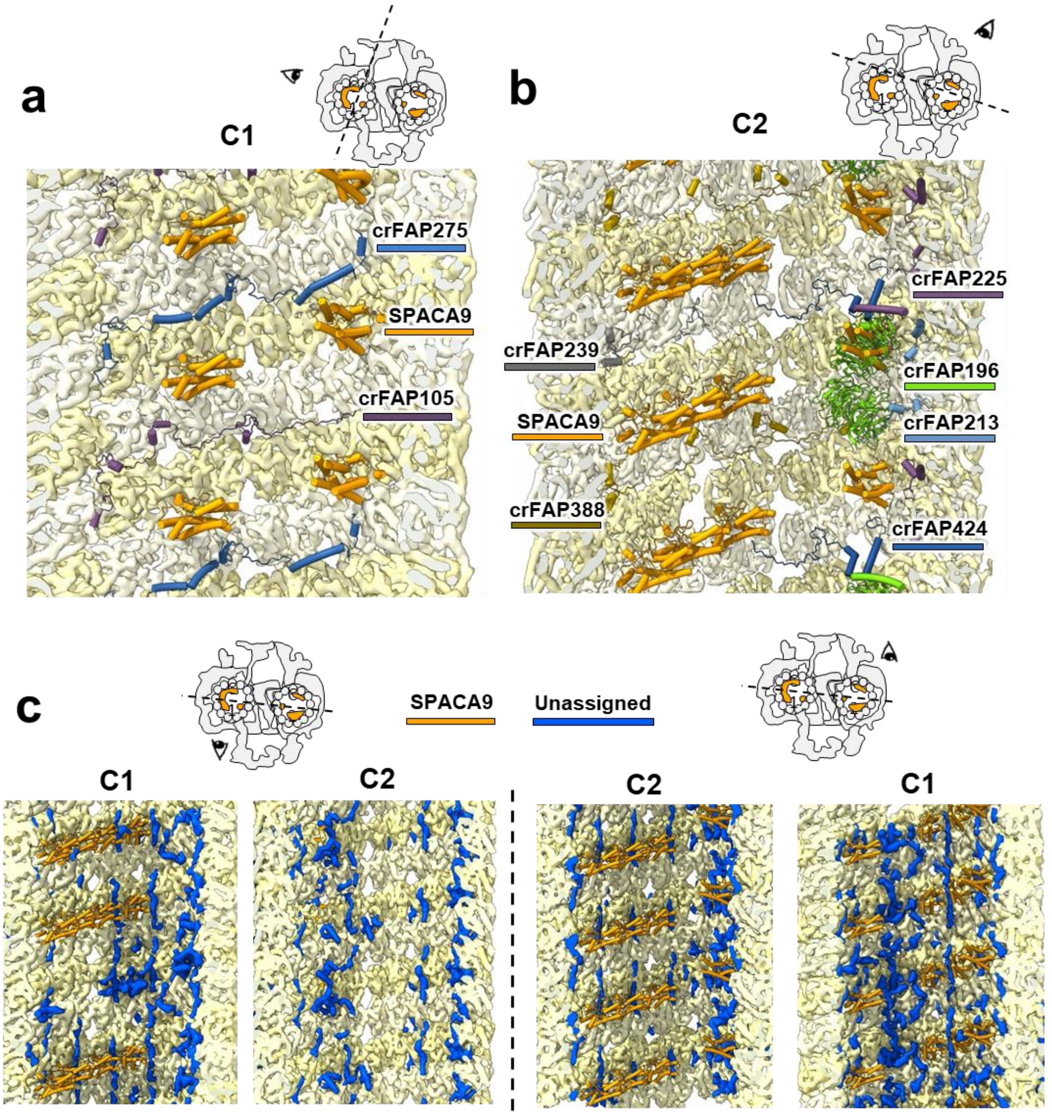

Fig. S23 Structural comparison of MIPs in mouse sperm and *C. reinhardtii* CA. a The C1 microtubule MIPs (crFAP105 and crFAP275) in *C. reinhardtii* CA (PDB entry 7SQC) are superimposed onto our CA structure, compared with our SPACA9 models. **b** The C2 microtubule MIPs (crFAP239, crFAP388, crFAP225, crFAP196, crFAP213 and crFAP424) in *C. reinhardtii* CA (PDB entry 7SOM) are superimposed onto our CA structure, compared with our SPACA9 models. **c** Unassigned MIP densities in the structure of mouse sperm CA.
