# Supplementary material for: In situ structure of the mouse sperm central apparatus reveals mechanistic insights into asthenozoospermia
Source: Cell Res. 2025 Jun 5;35(8):551–67. doi: 10.1038/s41422-025-01135-2 (PMC12297659; doi:10.1038/s41422-025-01135-2)
Supplement: Supplementary file 24 — Supplementary information, Figure S24 [file 41422_2025_1135_MOESM24_ESM.pdf]

Supplementary information, Figure S24

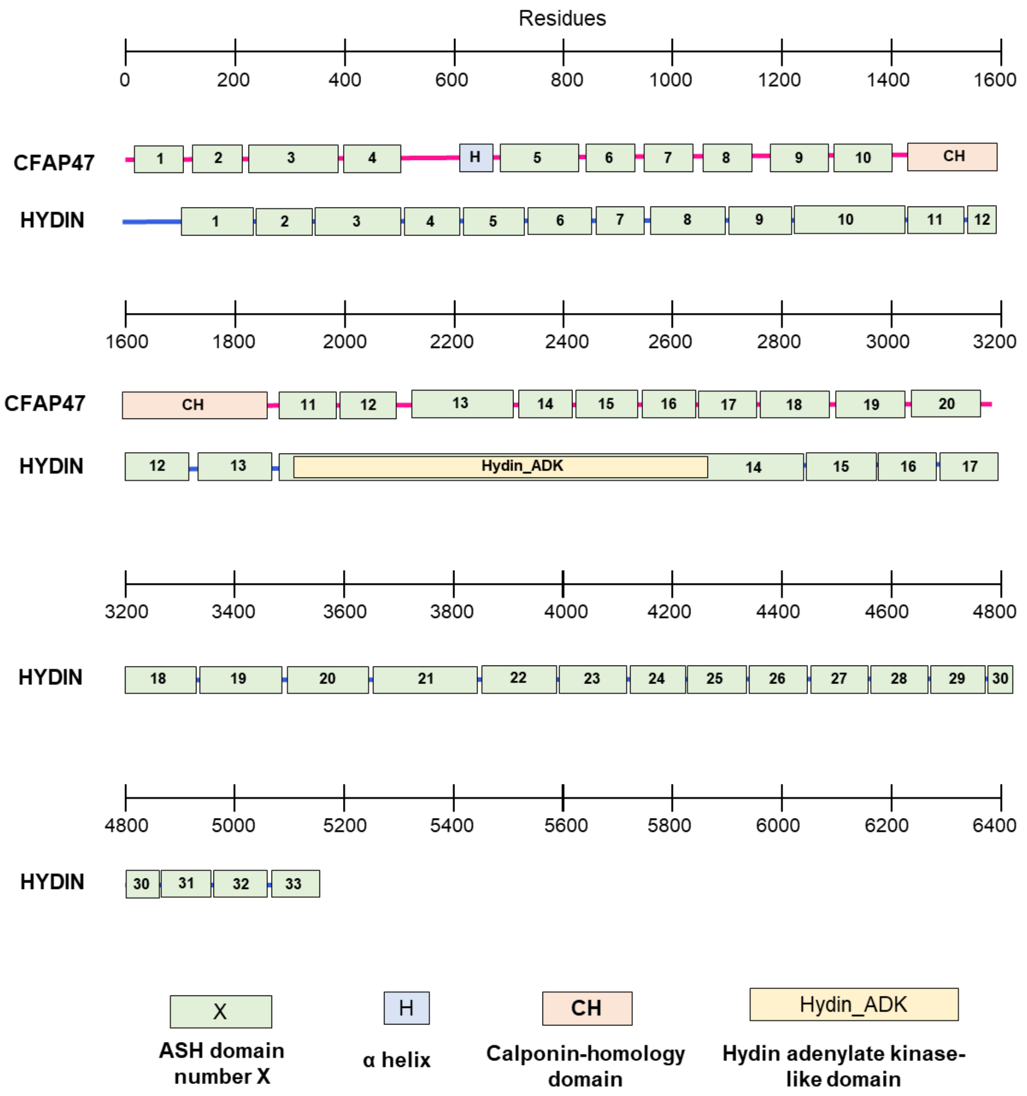

Fig. S24 Domain organization of mouse CFAP47 and HYDIN proteins. CFAP47 contains 20 ASH domains, and HYDIN contains 33 ASH domains. The scale indicates the sequence number of the residues, with each line representing 1600 residues.
