# Supplementary material for: In situ structure of the mouse sperm central apparatus reveals mechanistic insights into asthenozoospermia
Source: Cell Res. 2025 Jun 5;35(8):551–67. doi: 10.1038/s41422-025-01135-2 (PMC12297659; doi:10.1038/s41422-025-01135-2)
Supplement: Supplementary file 25 — Supplementary information, Figure S25 [file 41422_2025_1135_MOESM25_ESM.pdf]

Supplementary information, Figure S25

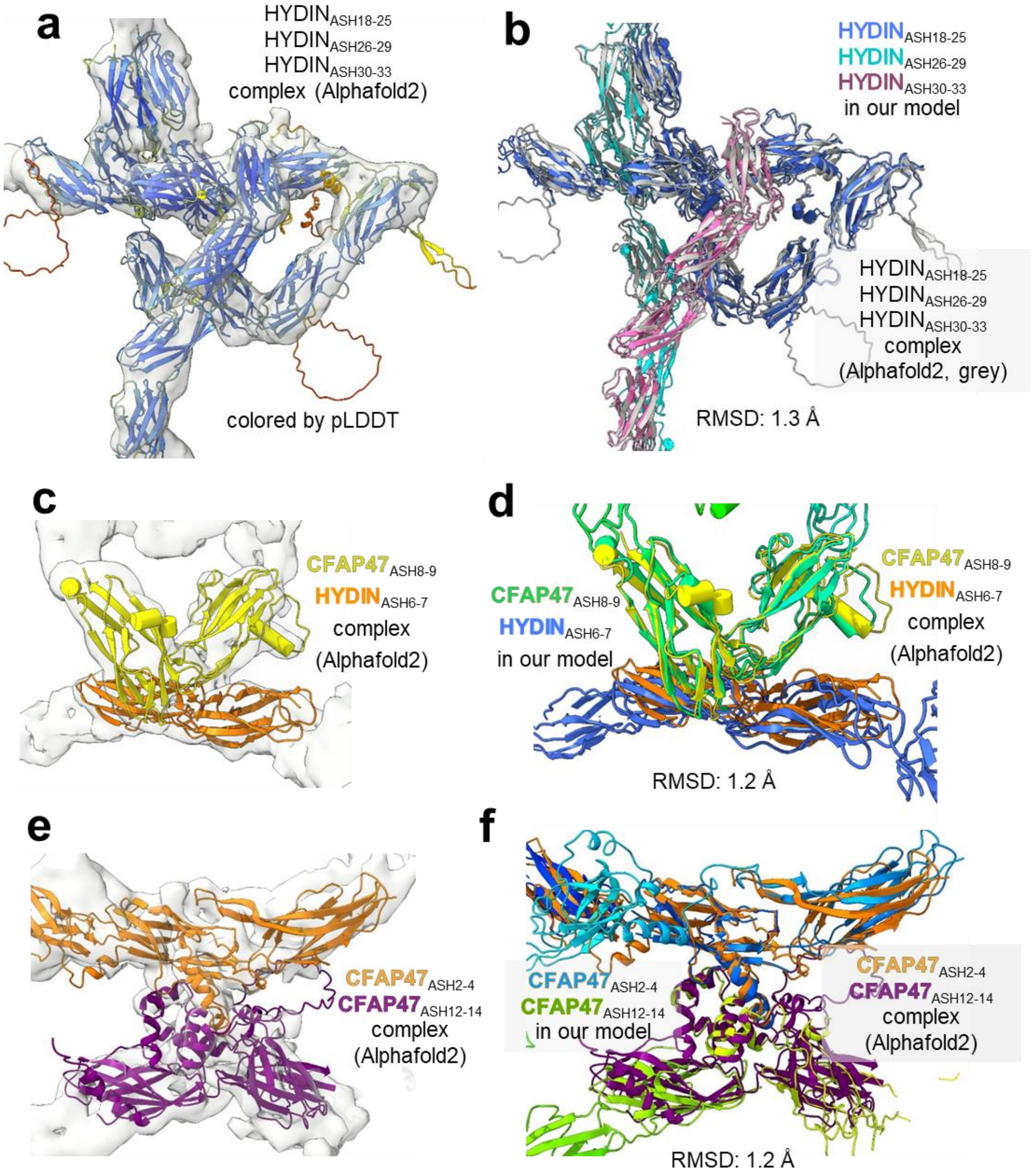

Fig. S25 Structural details of mouse HYDIN and CFAP47. a The Alphafold2 predicted structure of HYDIN CTD trimer, including ASH18-25 domains, ASH26-29 domains, and ASH30-33 domains, is directly fitted into our density map. **b** The Alphafold2 predicted structure in (**a**) (grey) is superposed with the corresponding structure in our model (blue, cyan and pink). RMSD values were calculated using the Matchmaker tool in ChimeraX, considering only aligned atom pairs. **c** The Alphafold2 predicted structure of mouse CFAP47 ASH8-9 domains (yellow) and HYDIN ASH6-7 domains (coral) is directly fitted into our density map. **d** The Alphafold2 predicted structure in (**c**) (yellow and coral) is superposed with the corresponding structure in our model (green and blue). RMSD values were calculated using the Matchmaker tool in

ChimeraX, considering only aligned atom pairs. **e** The Alphafold2 predicted structure of mouse CFAP47 ASH2-4 domains (coral) and ASH12-14 domains (violet) is directly fitted into our density map. **f** The Alphafold2 predicted structure in (**e**) (coral and violet) is superposed with the corresponding structure in our model (blue and green). RMSD values were calculated using the Matchmaker tool in ChimeraX, considering only aligned atom pairs.
