# Supplementary material for: In situ structure of the mouse sperm central apparatus reveals mechanistic insights into asthenozoospermia
Source: Cell Res. 2025 Jun 5;35(8):551–67. doi: 10.1038/s41422-025-01135-2 (PMC12297659; doi:10.1038/s41422-025-01135-2)
Supplement: Supplementary file 26 — Supplementary information, Figure S26 [file 41422_2025_1135_MOESM26_ESM.pdf]

Supplementary information, Figure S26

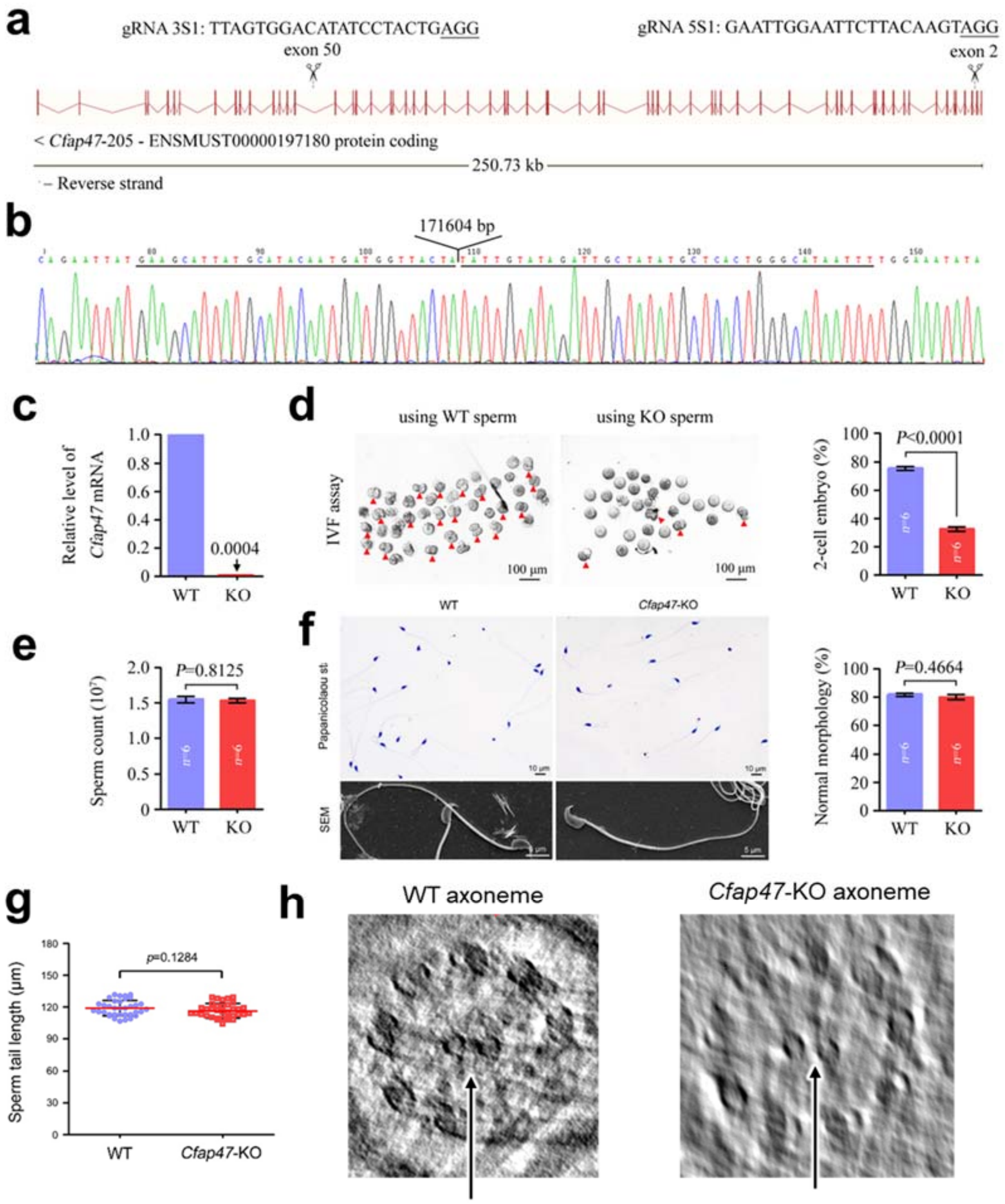

Fig. S26 Details of *Cfap47*-KO mice sperm. **a** Genomic features and knockout strategy of mouse *Cfap47* by CRISPR/Cas9 technology. **b** A Sanger sequencing showing a 171604-bp deletion in the *Cfap47* gene. **c** qRT-PCR analysis of *Cfap47* mRNA expression in testis samples. *Actin* served as an internal control. **d** Representative 2-cell embryos (arrowheads) from IVF in mice (scale bars, 100 μm). The percentage of 2-cell embryos in groups using sperm from WT or *Cfap47*-KO mice. **e** Sperm count using a fertility counting chamber. **f** Sperm morphology by Papanicolaou stain (scale bars, 10 μm) and SEM (scale bars, 5 μm). The percentage of sperm with normal morphology in WT and *Cfap47*-KO mice. **g** Statistical analysis of sperm tail

length in WT and *Cfap47*-KO mice. Student's *t* test; error bars represent standard error of the mean (n=5). **h** Tomogram slice of 9+2 axoneme structure in WT or *Cfap47*-KO mouse sperm, visualized using Slicer Mode in IMOD based on deconvoluted tomograms from Warp. Differences in CA bridge is indicated by arrowheads. Raw data can be found in Supplementary information, Table S11.
