# Supplementary material for: In situ structure of the mouse sperm central apparatus reveals mechanistic insights into asthenozoospermia
Source: Cell Res. 2025 Jun 5;35(8):551–67. doi: 10.1038/s41422-025-01135-2 (PMC12297659; doi:10.1038/s41422-025-01135-2)
Supplement: Supplementary file 27 — Supplementary information, Figure S27 [file 41422_2025_1135_MOESM27_ESM.pdf]

Supplementary information, Figure S27

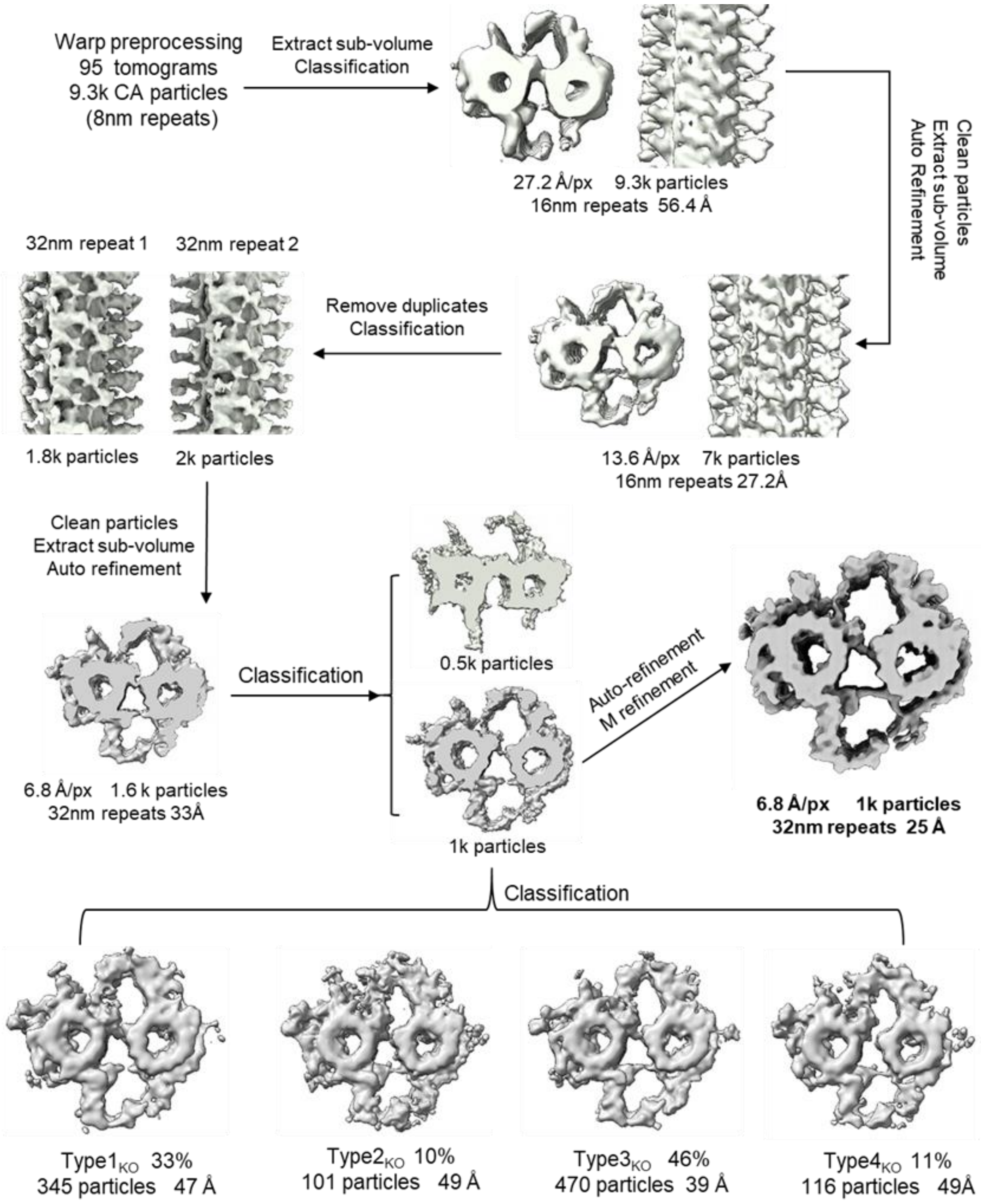

Fig. S27 Data processing procedure for sperm CA structure from *Cfap47*-KO mice.
