# Supplementary material for: In situ structure of the mouse sperm central apparatus reveals mechanistic insights into asthenozoospermia
Source: Cell Res. 2025 Jun 5;35(8):551–67. doi: 10.1038/s41422-025-01135-2 (PMC12297659; doi:10.1038/s41422-025-01135-2)
Supplement: Supplementary file 28 — Supplementary information, Figure S28 [file 41422_2025_1135_MOESM28_ESM.pdf]

Supplementary information, Figure S28

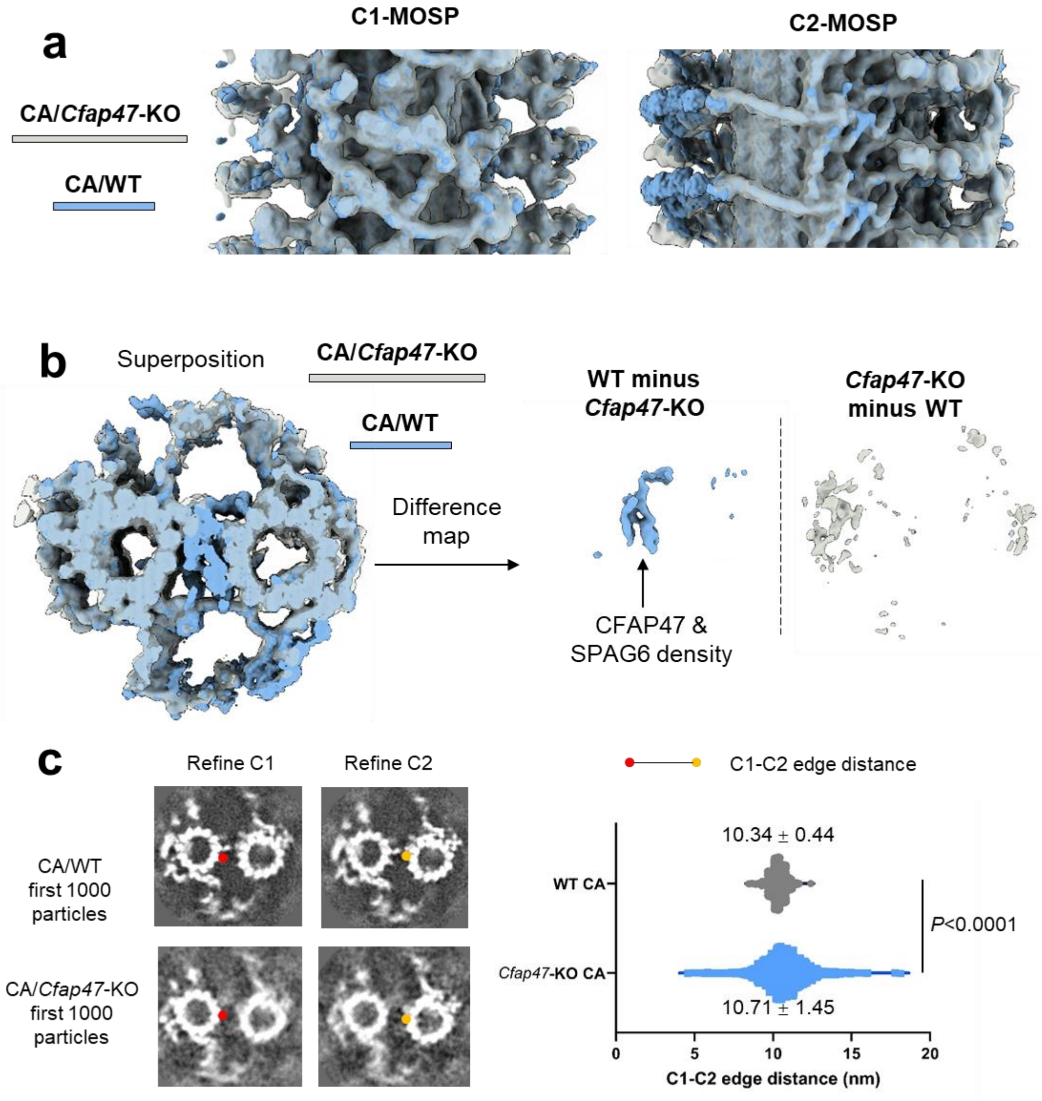

Fig. S28 Structural comparison of CA in *Cfap47*-KO and WT mice. a Side views of CA structure in *Cfap47*-KO mice (grey) overlaid with WT CA structure (blue). **b** Difference maps of sperm CA between WT and *Cfap47*-KO mice. **c** From the WT and *Cfap47*-KO datasets, we selected the first 1,000 particles and aligned them, respectively, to C1 and C2 microtubule. From the C1-refined data, we defined the C1 edge (red dot) at the 432aa of β-tubulin on protofilament 13 of the C1 microtubule. From the C2-refined data, we defined the C2 edge (yellow dot) at the 432aa of β-tubulin on protofilament 1 of the C2 microtubule. We then calculated the edge distance between C1 and C2 in both datasets. The distances data are presented as mean ± SD (n = 1,000). A two-tailed t-test was used for statistical analysis (*P*<0.0001).
