# Supplementary material for: In situ structure of the mouse sperm central apparatus reveals mechanistic insights into asthenozoospermia
Source: Cell Res. 2025 Jun 5;35(8):551–67. doi: 10.1038/s41422-025-01135-2 (PMC12297659; doi:10.1038/s41422-025-01135-2)
Supplement: Supplementary file 29 — Supplementary information, Figure S29 [file 41422_2025_1135_MOESM29_ESM.pdf]

Supplementary information, Figure S29

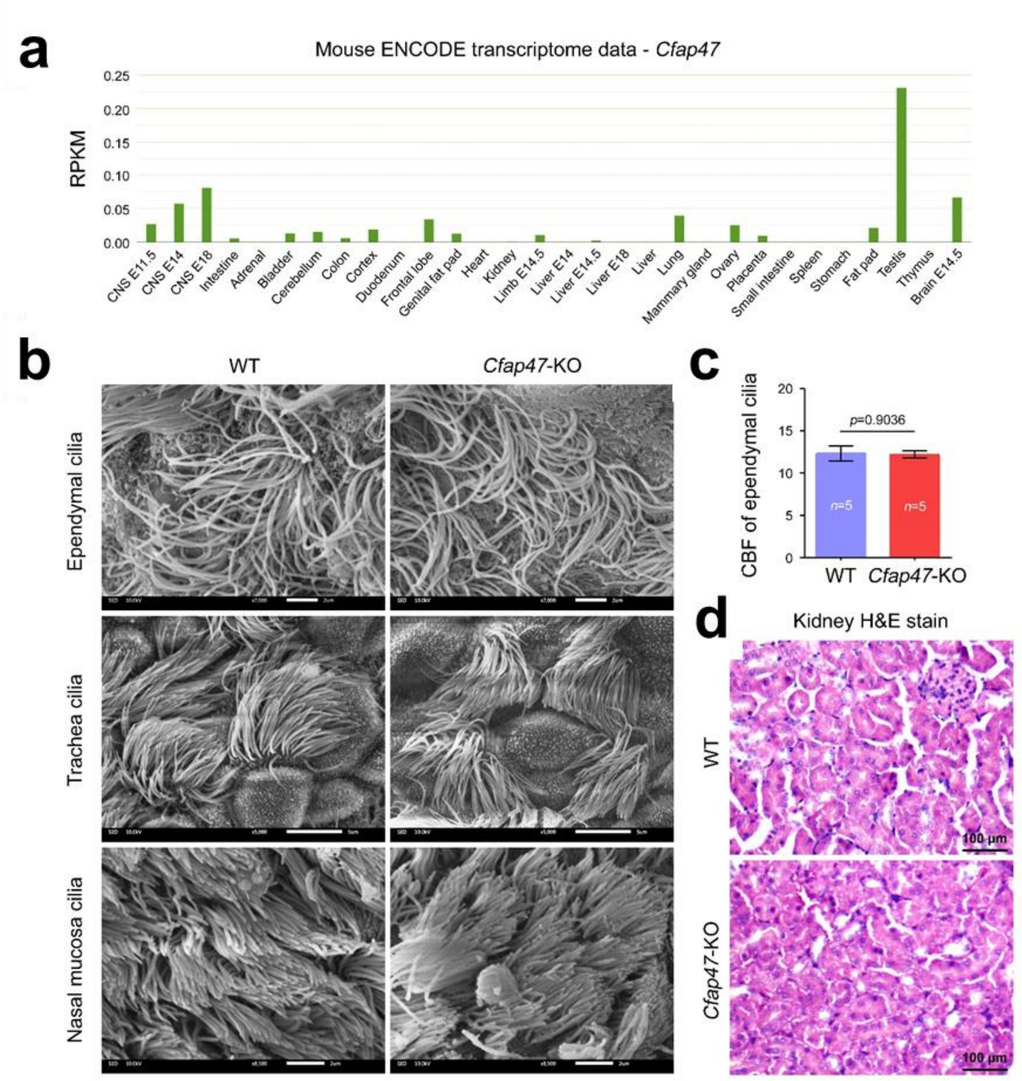

Fig. S29 The effects of *Cfap47*-KO on other cilia tissues. a The relative mRNA level of *Cfap47* was revealed by mouse ENCODE transcriptome data. **b** SEM analyses of ependymal cilia, trachea cilia, and nasal mucosa cilia in wild-type (WT) mice and *Cfap47*-KO mice. Scale bars, 2 µm or 5 µm. **c** The ciliary beat frequency (CBF) was calculated from video microscopy of ependymal cilia. Student's *t* test; error bars represent standard error of the mean (*n*=5). **d** The representative morphology of kidney tissues in 10-week-old WT mice and *Cfap47*-KO mice as revealed by hematoxylin and eosin (H&E) staining. Scale bar, 100 µm.
