# Supplementary material for: In situ structure of the mouse sperm central apparatus reveals mechanistic insights into asthenozoospermia
Source: Cell Res. 2025 Jun 5;35(8):551–67. doi: 10.1038/s41422-025-01135-2 (PMC12297659; doi:10.1038/s41422-025-01135-2)
Supplement: Supplementary file 30 — Supplementary information, Figure S30 [file 41422_2025_1135_MOESM30_ESM.pdf]

Supplementary information, Figure S30

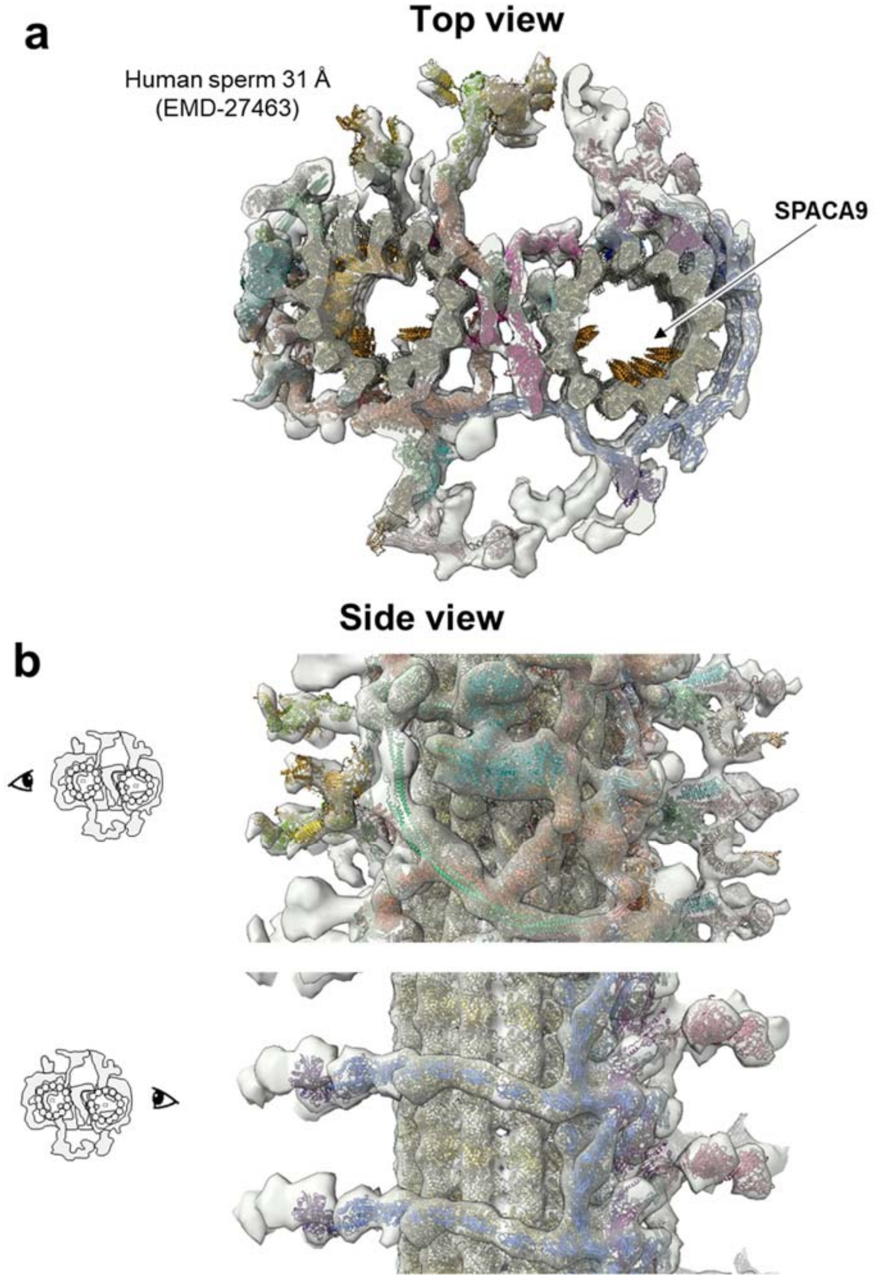

Fig. S30 Our structure model of mouse sperm CA fits well into human CA density map, shown in both top view (a) and side (b). The low-resolution map of human sperm CA (EMD-27463) is displayed in transparent gray.
