# Supplementary material for: In situ structure of the mouse sperm central apparatus reveals mechanistic insights into asthenozoospermia
Source: Cell Res. 2025 Jun 5;35(8):551–67. doi: 10.1038/s41422-025-01135-2 (PMC12297659; doi:10.1038/s41422-025-01135-2)
Supplement: Supplementary file 31 — Supplementary information, Figure S31 [file 41422_2025_1135_MOESM31_ESM.pdf]

Supplementary information, Figure S31

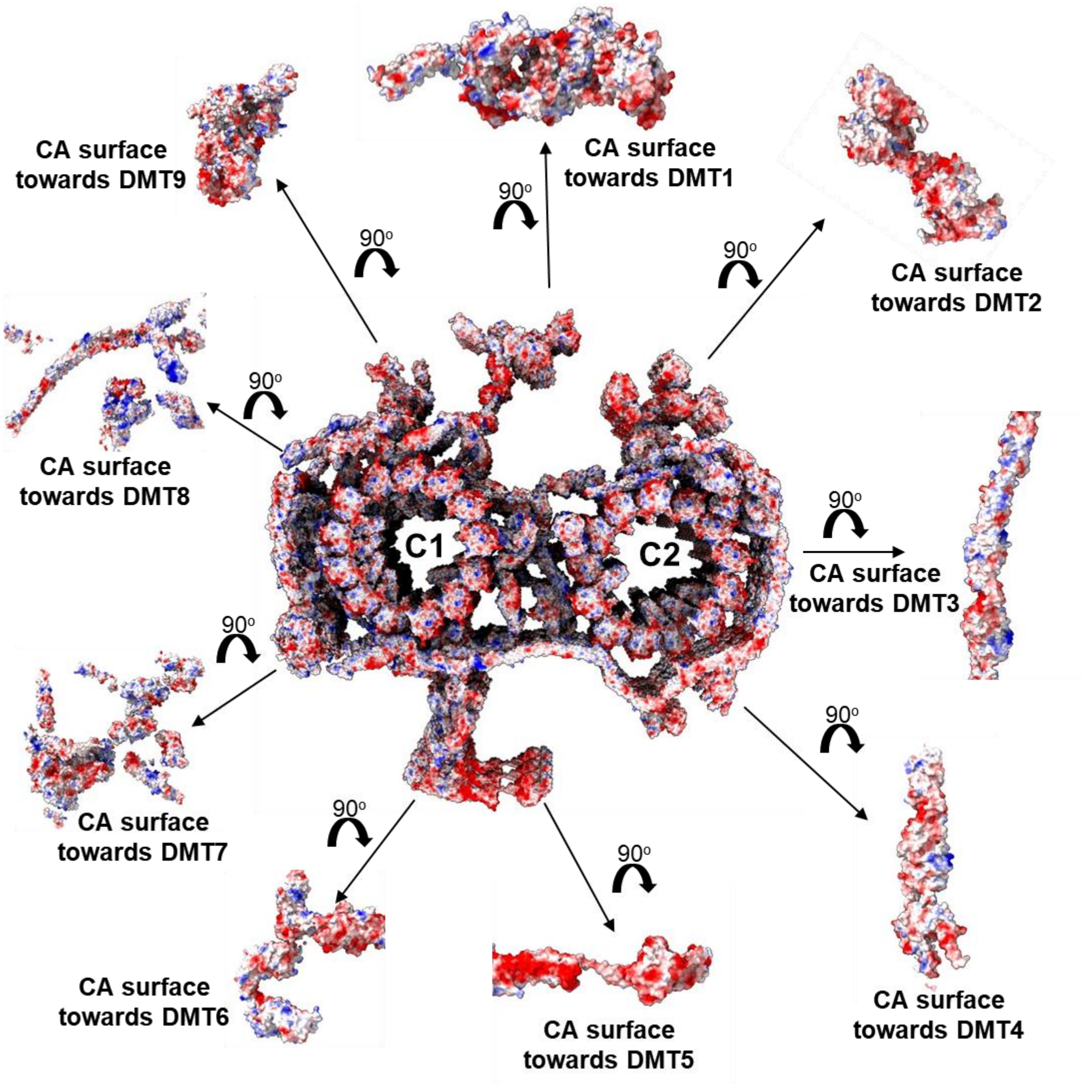

Fig. S31 Surface electrostatics of mouse sperm CA for the overall structure and the surfaces towards 9 DMTs. Red represents a negatively charged surface, while blue represents a positively charged surface.
