# Supplementary material for: In situ structure of the mouse sperm central apparatus reveals mechanistic insights into asthenozoospermia
Source: Cell Res. 2025 Jun 5;35(8):551–67. doi: 10.1038/s41422-025-01135-2 (PMC12297659; doi:10.1038/s41422-025-01135-2)
Supplement: Supplementary file 32 — Supplementary information, Table S1 [file 41422_2025_1135_MOESM32_ESM.pdf]

Supplementary information, Table S1. Statistics of cryo-ET data collection, image processing and model building.

**Data acquisition**		
Data set	WT sperm CA	*Cfap47*-KO sperm CA
Microscope	Titan Krios G2	
Voltage (kV)	300	
Detector	Gatan K2	
Energy filter	Gatan GIF Quantum, 20 eV	
Mode	Super resolution	Counting mode
Pixel size (Å)	0.88	3.4
Stage tilting angle	-50° to 67° or -66° to 51°	
Number of images	40	
Exposure per image (e/Å²)	3 or 3.5	
Exposure per tilt (e/Å²)	120 to 140	
Defocus range (µm)	-1 to -4	
Software	SerialEM	
**Sub tomogram analysis**		
Software	RELION-3.1/Warp	
Data set	WT sperm CA	*Cfap47*-KO sperm CA
EMDB ID entry	EMD-60633*(composite map)	EMD-60597
Number of tomograms	1889	95
Number of particles	33796	1050
Symmetry	C1	
Resolution (Å)	6.6 ~ 18	25
Map pixel size (Å)	2.64	6.8
**Model building and refinement**		
Data set	WT mouse sperm CA	
PDB entry	9IJJ	
Rmsd (bond) (Å)	0.005	
Rmsd (angle) (°)	1.011	
MolProbity score	2.04	
No. of residues	207000	
No. of subunits	466	
Ramachandran plot		
Outliers	0.1	
Allowed	4.85	
Favoured	95.05	
Rotamer outliers (%)	0.14	

*This map was composed by ten maps, including C1 (EMD-60428, 7.7 Å), C1a (EMD-60429, 9.4 Å), C1b (EMD-60430, 8.9 Å), C2 (EMD-60431, 6.6 Å), C2a (EMD-60436, 7.8 Å), C2b (EMD-60446, 9.6 Å), Cm (EMD-60448, 7.7 Å), Cma1 (EMD-60449, 18 Å), Cma2 (EMD-60454, 16 Å), and Cmb (EMD-60455, 15 Å).
