# Supplementary material for: In situ structure of the mouse sperm central apparatus reveals mechanistic insights into asthenozoospermia
Source: Cell Res. 2025 Jun 5;35(8):551–67. doi: 10.1038/s41422-025-01135-2 (PMC12297659; doi:10.1038/s41422-025-01135-2)
Supplement: Supplementary file 33 — Supplementary information, Table S2 [file 41422_2025_1135_MOESM33_ESM.pdf]

Supplementary information, Table S2. The mass spectrometry assay and previously reported proteomes of mouse sperm verified the presence of the CA component proteins in mouse sperm.

Primary Location	Components	Full name	Uniprot ID	MS list*	Proteomes[18]
Microtubule wall	Tubulin α3	Tubulin alpha-3 chain	P05214	√	√
	Tubulin β-4B	Tubulin beta-4B chain	P68372	√	√
C1-MOSP	CFAP46	Cilia and flagella associated protein 46	E9Q2C0	√	-
	CFAP54	Cilia- and flagella-associated protein 54	Q8C6S9	√	√
	CFAP74	Cilia- and flagella-associated protein 74	Q3UY96	√	√
	CFAP99	Cilia and flagella associated protein 99	A0A140LIX9	√	-
	CFAP221	Cilia- and flagella-associated protein 221	A9Q751	√	√
	LRRC72	Leucine rich repeat containing 72	A0A1Y7VMI0	√	-
	CCDC180	Coiled-coil domain containing 180	J3QNE4	√	-
	DLEC1	Deleted in lung and esophageal cancer 1	E9Q8C0	√	Q8BLA1 (isoform)
	SPAG6	Sperm-associated antigen 6	Q9JLI7	√	√
	GRK3	G protein-coupled receptor kinase 3	Q3UYH7	√	√
	PPP1CC	Serine/threonine-protein phosphatase PP1-gamma catalytic subunit	P63087	√	√
	ANKMY1	Ankyrin repeat and MYND domain-containing protein 1	Q8C0W1	√	√
	LRRC43	Leucine-rich repeat-containing protein 43	Q3V0L5	√	√
C1a arm	CFAP119	Cilia- and flagella-associated protein 119	Q6NZQ0	√	√
	Calmodulin	Calmodulin-1	P0DP26	P0DP27**	P0DP27**
	MORN2	MORN repeat containing 2	A0A2I3BRK9	-	Q6UL01 (isoform)
	DPY30	Protein dpy-30 homolog	Q99LT0	√	√
	SPAG17	Sperm-associated antigen 17	Q5S003	√	√
	SPATA17	Spermatogenesis-associated protein 17	Q9D552	√	√
C1b arm	CFAP69	Cilia- and flagella-associated protein 69	Q8BH53	√	√
	SPEF2	Sperm flagellar protein 2	Q8C9J3	√	√
	LRGUK	Leucine-rich repeat and guanylate kinase domain-containing protein	Q9D5S7	√	√
	GOT1L1	Putative aspartate aminotransferase, cytoplasmic 2	Q7TSV6	√	√

	LRRD1	Leucine-rich repeat and death domain-containing protein 1	Q8C0R9	√	√
C2-MOSP	SPAG16	Sperm-associated antigen 16 protein	Q8K450	√	√
	KIF9	Kinesin-like protein KIF9	Q9WV04	√	√
	FAM228B	Protein FAM228B	Q497Q6	√	√
	CFAP20	Cilia- and flagella-associated protein 20	Q8BTU1	√	√
	MYCBPAP	MYCBP-associated protein	Q5SUV2	√	√
	SPATA4	Spermatogenesis-associated protein 4	Q8K3V1	√	√
C2a arm	CFAP65	Cilia- and flagella-associated protein 65	Q3V0B4	√	√
	CFAP70	Cilia- and flagella-associated protein 70	D3YVL2	√	√
C2b arm	HYDIN	Hydrocephalus-inducing protein	Q80W93	√	√
	MAP1S	Microtubule-associated protein 1S	Q8C052	-	√
Bridge	CFAP47	Cilia and flagella-associated protein 47	A0A0G2JEB6	√	-
	GMCL1(BTBD16)	Germ cell-less protein-like 1(BTB/POZ domain-containing protein 16)	Q920G9 (E9Q173)	-(√)	√(√)
MIP	SPACA9	Sperm acrosome-associated protein 9	Q7TPM5	√	√

* This list is updated from the mass spectrometry results previously reported[17], with the searching parameters being reset. The complete protein list from this MS analysis is shown in Supplementary information, Table S9.

** P0DP27 has the identical amino acid sequence as P0DP26.
