# Supplementary material for: In situ structure of the mouse sperm central apparatus reveals mechanistic insights into asthenozoospermia
Source: Cell Res. 2025 Jun 5;35(8):551–67. doi: 10.1038/s41422-025-01135-2 (PMC12297659; doi:10.1038/s41422-025-01135-2)
Supplement: Supplementary file 34 — Supplementary information, Table S3 [file 41422_2025_1135_MOESM34_ESM.pdf]

Supplementary information, Table S3. Comparison of the CA structure of mouse sperm with that of *C. reinhardtii* cilia.

Shared components

**Mouse**	***C. reinhardtii***	**Mouse**	***C. reinhardtii***	**Mouse**	***C. reinhardtii***
Tubulin α3	Tubulin α	CFAP119	FAP119	MYCBPAP	FAP147
Tubulin β4	Tubulin β	Calmodulin	Calmodulin	SPATA4	FAP178
CFAP46	FAP46	MORN2	FAP227	CFAP65	FAP65
CFAP54	FAP54	DPY30	DPY30	CFAP70	FAP70
CFAP74	FAP74	SPAG17	PF6	CFAP99	FAP99
CFAP221	FAP221	SPATA17	FAP305	KIF9	Kinesin-like protein
LRRC72	FAP279	CFAP69	FAP69	HYDIN	HYDIN
CCDC180	FAP76	SPEF2	CPC1	MAP1S	FAP312*
DLEC1	FAP81	LRGUK	FAP246	CFAP47	FAP47
SPAG6	PF16	SPAG16	PF20	HSP70*	HSP70
PPP1CC	FAP15	FAM228B	FAP239	Gamma-enolase*	phosphopyruvate hydratase
CFAP119	FAP114	CFAP20	FAP20	CFAP52*	FAP196

Species-specific component

**Mouse**	**C. reinhardtii**	**Top 1 in blast result****	**NCBI accession ID**	**Query coverage**	**percent identity**
GRK3	No homolog	Phototropin	Q8LPD9.1	42%	29.89%
ANKMY1	No homolog	Flagellar radial spoke protein 1	Q27YU0.1	8%	35.06%
LRRC43	No homolog		No significant similarity found.		
GOT1L1	No homolog		No significant similarity found.		
LRRD1	No homolog		No significant similarity found.		
GMCL1	No homolog		No significant similarity found.		
BTBD16	No homolog		No significant similarity found.		
SPACA9	No homolog		No significant similarity found.		
No homolog	FAP7		No significant similarity found		
No homolog	FAP42	Guanylate kinase	Q64520.2	8%	40.00%
No homolog	FAP92		No significant similarity found.		
No homolog	FAP101	Dynein regulatory complex subunit 5	A6H639.1	18%	28.74%
No homolog	FAP105		No significant similarity found.		
No homolog	FAP108		No significant similarity found.		
No homolog	FAP174	c-Myc-binding protein	Q9EQS3.5	73%	42.86%
No homolog	FAP194	Sperm-associated antigen 6	Q9JLI7.1	96%	27.65%
No homolog	FAP216		No significant similarity found.		
No homolog	FAP219		No significant similarity found.		
No homolog	FAP266	Radial spoke head 10 homolog B	E9PYQ0.1	35%	41.79%
No homolog	FAP275		No significant similarity found.		
No homolog	FAP289		No significant similarity found.		
No homolog	FAP297		No significant similarity found.		
No homolog	HTH_9 domain	CFAP221	A9Q751.1	7%	34.67%

	containing protein				
No homolog	FAP213		No significant similarity found.		
No homolog	FAP225	Calcyphosin-like protein	Q6P8Y1.4	7%	38.18%
No homolog	FAP239		No significant similarity found.		
No homolog	FAP388		No significant similarity found.		
No homolog	FAP424		No significant similarity found.		

*These homologous proteins have not been found in the CA structures of their respective species.

**Homolog proteins were searched using the NCBI BLAST tool, with the selected species being either *C. reinhardtii* (taxid: 3055) or mouse (taxid: 10090).
