# Supplementary material for: In situ structure of the mouse sperm central apparatus reveals mechanistic insights into asthenozoospermia
Source: Cell Res. 2025 Jun 5;35(8):551–67. doi: 10.1038/s41422-025-01135-2 (PMC12297659; doi:10.1038/s41422-025-01135-2)
Supplement: Supplementary file 36 — Supplementary information, Table S5 [file 41422_2025_1135_MOESM36_ESM.pdf]

Supplementary information, Table S5. Semen analysis using CASA in patient 1 and 2 with *CFAP47* deficiency.

	Patient 1*	Patient 2*	Control**	Reference value***
Age (years)	31	33	33.4 ± 5.7	-
Chromosome karyotype	46,XY	46,XY	46,XY	46,XY
Duration of infertility (years)	2	5	-	-
**Semen parameters**				
Semen volume (mean ± SD, mL)	2.2 ± 0.2	2.0 ± 0.2	3.6 ± 1.4	≥ 1.4
Semen pH (mean ± SD)	7.2 ± 0.0	7.3 ± 0.1	7.3 ± 0.1	≥ 7.2
Sperm concentration (mean ± SD, 10^6/mL)	24.5 ± 6.4	114.3 ± 12.7	99.6 ± 59.5	≥ 16
Total sperm count (mean ± SD, 10^6/ejaculate)	53.5 ± 19.1	227.1 ± 14.2	333.3 ± 216.6	≥ 39
Rapid progressive motility (Grade a, mean ± SD, %）	2.4 ± 3.3	4.9 ± 0.9	28.1 ± 14.8	-
Slow progressive motility (Grade b, mean ± SD, %）	5.3 ± 1.8	9.2 ± 1.8	23.3 ± 7.0	-
Non-progressive motility (Grade c, mean ± SD, %）	7.6 ± 3.5	6.4 ± 0.6	8.4 ± 3.8	-
Immotility (Grade d, mean ± SD, %）	84.9 ± 1.6	79.6 ± 2.6	40.2 ± 13.3	-
Progressive motility (a + b, mean ± SD, %)	7.6 ± 5.1	14.1 ± 2.4	51.4 ± 13.7	≥ 30
Total sperm motility (a + b + c, mean ± SD, %)	15.2 ± 1.6	20.5 ± 2.6	59.8 ± 13.3	≥ 42
**Sperm locomotion parameters**				
Curvilinear velocity (VCL) (mean ± SD, μm/s)	37.9 ± 10.5	39.2 ± 0.7	55.7 ± 11.8	-
Straight-line velocity (VSL) (mean ± SD, μm/s)	16.3 ± 4.5	15.9 ± 0.1	25.2 ± 5.6	-
Average path velocity (VAP) (mean ± SD, μm/s)	11.2 ± 1.3	10.5 ± 0.2	16.1 ± 2.9	-
Mean angular displacement(MAD, °)	30.8 ± 10.3	32.4 ± 1.1	37.2 ± 5.6	-
Beat-cross frequency (mean ± SD, BCF, Hz)	4.7 ± 3.3	5.6 ± 0.1	7.5 ± 1.8	-
Amplitude of lateral head displacement (ALH) (mean ± SD, μm)	0.7 ± 0.2	0.8 ± 0.1	0.9 ± 0.2	-
Linearity (LIN) (mean ± SD)	0.9 ± 0.0	0.9 ± 0.1	0.9 ± 0.1	-
Wobble (mean ± SD, WOB, = VAP/VCL)	0.6 ± 0.1	0.6 ± 0.1	0.6 ± 0.1	-
Straightness (mean ± SD, STR, = VSL/VAP)	1.5 ± 0.2	1.6 ± 0.1	1.5 ± 0.1	-

*Three different semen samples.

**100 cases of normal males.

*** The reference values refer to the World Health Organization laboratory manual for the examination and processing of human semen. 6th Edition. “-”: no reference range.

CASA: computer-assisted sperm analysis.
