# Supplementary material for: In situ structure of the mouse sperm central apparatus reveals mechanistic insights into asthenozoospermia
Source: Cell Res. 2025 Jun 5;35(8):551–67. doi: 10.1038/s41422-025-01135-2 (PMC12297659; doi:10.1038/s41422-025-01135-2)
Supplement: Supplementary file 37 — Supplementary information, Table S6 [file 41422_2025_1135_MOESM37_ESM.pdf]

Supplementary information, Table S6. The clinical outcomes of *CFAP47* mutation patient 1's wife (L153 II-5) receiving ART treatment.

Characteristics	L153 II-5
**Basal information**	
Female age (years)	26
BMI (kg/m^2)	17.67
AMH (ng/mL)	4.46
FSH (mIU/mL)	6.56
LH (mIU/mL)	4.23
E2 (pg/mL)	28
PRL (ng/mL)	9.35
**Ovarian stimulation**	
Protocol	Short-acting GnRH-agonist long protocol
E2 level on the trigger day (pg/mL)	2914
No. of follicles ≥ 14 mm on the trigger day	13
No. of follicles ≥ 18 mm on the trigger day	6
**Laboratory indicators**	
No. of oocytes retrieved	13
No. of MII oocytes (%)	13/13 (100)
Insemination method	ICSI
No. of 2PN (%)	11/13 (84.6)
No. of Day 3 transferable embryos (%)	9/11 (81.8)
No. of blastocysts (%)	7/9 (77.8)
No. of embryo transfer	1 (FET)
No. of implantation	0
No. of ongoing pregnancy/live birth	0

Abbreviations: BMI, body mass index; AMH, anti-Mullerian hormone; FSH, follicle-stimulating hormone; LH, luteinizing hormone; E2, estradiol; PRL, prolactin; GnRH, gonadotrophin-releasing hormone; MII, metaphase II; 2PN, two pronucleus; FET, frozen-thawed embryo transfer.
