# Supplementary material for: In situ structure of the mouse sperm central apparatus reveals mechanistic insights into asthenozoospermia
Source: Cell Res. 2025 Jun 5;35(8):551–67. doi: 10.1038/s41422-025-01135-2 (PMC12297659; doi:10.1038/s41422-025-01135-2)
Supplement: Supplementary file 38 — Supplementary information, Table S7 [file 41422_2025_1135_MOESM38_ESM.pdf]

Supplementary information, Table S7. The disease-associated mutations that are related to CA components in mouse sperm.

Proteins	Uniprot ID Human/Mouse	Human mutations	Corresponding site in Mouse
CFAP54	Q96N23 /Q8C6S9	c.3317del (p.Phe1106Serfs*19)[74]	F1110S fs
		c.4885C>T (p.Arg1629Cys)[74]	R1634C
		c.937G>A (p.Gly313Arg)[74]	G318R
		c.2649_2657delinC (p.Glu883Aspfs*47)[75]	E887D fs
		c.7312_7313insCGCAGGCTGAATTCTTGG (p.T2438delinsTQAEFLA)[75]	M2447 delins
CFAP74	Q9C0B2 /Q3UY96	c.907del (p.Gln303LysfsTer65)[76]	Q300K fs
		c.4380_4381dup (p.Phe1461SerfsTer12)[76]	F1455S fs
		c.1706dup (p.Gly570TrpfsTer10)[76]	G567W fs
		c.983G>A (p.Gly328Asp)[77]	G325D
SPAG6	O75602 /Q9JLI7	c.308C>A (p.Ala103Asp)[78]	A103D
		c.585delA (p.Lys196Serfs*6)[78,79]	K196S fs
		c.143_145del (p.48_49del)[79]	P48L49-del
		c.C928T (p.Arg310Trp)[80]	R310W
CFAP69	A5D8W1 /Q8BH53	c.2061dup (p.Pro688Thrfs*5)[81]	P689T fs
		c.1069_1070insAC (p.Leu357Hisfs*11)[82]	L358H fs
		c.647G>A (p.Trp216*)[82]	W217*
		c.860+1G>A (p. splicing)[83]	splicing
		c.763C>T (p.Gln255*)[83]	Q256*
SPEF2	Q9C093 /Q8C9J3	c.910C>T (p.Arg304*)[84,85]	R247*
		c.3400delA (p.Ile1134Serfs*13)[40,84]	I1036S fs
		c.3240delT (p.Phe1080Leufs*2)[84]	F982L fs
		c.1860_1861insCT (p.Ala621Leufs*59)[86]	P564L fs
		c.4447+1G>A (p. splicing)[87]	splicing
		c.1339C>T (p.Arg447*)[87]	R390*
		c.1645G>T (p.Glu549*)[87]	N492*
		c.1693C>T (p.Arg547*)[88]	A490*
		c.2629del (p.Ile877Phefs*6)[85]	I811F fs
		c.2734delC (p.P912Lfs*74)[89]	M846L fs
		c.12delC (p.I4fs)[90]	I4 fs
		c.1745-2A>G (p. splicing) [90]	splicing
		c.4102G>T (p.E1368*)[90]	E1272*
		c.4323dupA (p.I1441fs)[90]	I1345 fs
		c.C4096T (p.Arg1366*)[40]	R1270*
		c.2649dupA (p.Asn887Glufs*2) [40]	I821E fs
		c.3922dupA (p.Lys1307fs)[40]	K1211 fs
SPAG16	Q8N0X2 /Q8K450	c.1464-1465insC; 1469-1470insA (p.Phe489Leufs*46)[91]	F497L fs
KIF9	Q9WV04 /Q9WV04	c.1433delinsA (p.N478Tfs*39)[92]	H478T fs
		c.1861A>T (p.K621*)[92]	K621*
CFAP20	Q9Y6A4 /Q8BTU1	c.337C>T (p.Arg113Trp)[93]	R113W
		c.397del (p.Gln133Serfs*5)[93]	Q133S fs
		c.164+1G>A (p. splicing)[93]	splicing
CFAP65	Q6ZU64 /Q3V0B4	c.C2284T (p.Arg762*)[94]	R690*
		c.1775delC (p.Pro592Leufs*8)[95]	P515L fs
		c.3072_3079dup (p.Arg1027Profs*41)[95]	R955P fs
		c.1946delC (p.Pro649Argfs*5)[95]	P577R fs
		c.1580delT (p.Leu527Argfs*31)[95]	L450R fs
		c.4855C>T (p.Arg1619*)[95]	R1551*

		c.2675G>A (p.Trp892*)[96]	W820*
CFAP70	Q5T0N1 /D3YVL2	c.1723-1G>T (p. splicing)[97]	splicing
		c.2962C>T (p.R988*)[98]	R1008*
HYDIN	Q4G0P3 /Q80W93	c.922A>T (p.Lys307*)[88,99]	K354*
		c.6140C>G (p.Ser2047*)[85,88]	S2083*
		c.2851C>T (p.Arg951*)[100]	R997*
		c.8487_8489delinsCA (p.Pro2830Hisfs*23)[101]	P2868H fs
		c.1529del (p.Phe510Serfs*43) [101]	F556S fs
		c.10886_10902del (p.Asp3629Valfs*9) [101]	S3666V fs
		c.14157T>G (p.Tyr4719*)[101]	Y4752*
		c.3252dup (p.Val1085Argfs*15)[101]	V1132R fs
		c.283C>T (p.Gln95*)[101]	Q141*
		c.2419_2422del (p.Val807llefs*13)[101]	I853I fs
		c.1095del (p.Phe365Leufs*64)[101]	I411L fs
		c.1147C>T (p.Arg383*)[101]	R429*
		c.4888A>T (p.Lys1630*)[101]	K1670*
		c.12144dup (p.Thr4049Hisfs*9) [101]	L4082H fs
		c.7214_7215del (p.Ser2405Cysfs*2) [101]	S2445C fs
		c.13709del (p.Pro4570Leufs*22) [101]	P4603L fs
		c.1003A>T (p.Lys335*)[88]	K380*
		c.2618T>G (p.Leu873Arg)[88]	L919R
		c.2616_2617ins TGGCACTGAC (p.Leu873Trpfs*3)[88]	L919W fs

74. Tian, S. *et al.* Biallelic mutations in CFAP54 cause male infertility with severe MMAF and NOA. *J Med Genet* **60**, 827-834 (2023).

75. Zhao, X. *et al.* Lack of CFAP54 causes primary ciliary dyskinesia in a mouse model and human patients. *Front Med* **17**, 1236-1249 (2023).

76. Biebach, L. *et al.* Recessive mutations in CFAP74 cause primary ciliary dyskinesia with normal ciliary ultrastructure. *Am J Respir Cell Mol Biol* **67**, 409-413 (2022).

77. Sha, Y. *et al.* Biallelic mutations of CFAP74 may cause human primary ciliary dyskinesia and MMAF phenotype. *J Hum Genet* **65**, 961-969 (2020).

78. Xu, C. *et al.* Homozygous SPAG6 variants can induce nonsyndromic asthenoteratozoospermia with severe MMAF. *Reprod Biol Endocrinol* **20**, 41 (2022).

79. Wu, H. *et al.* Patients with severe asthenoteratospermia carrying SPAG6 or RSPH3 mutations have a positive pregnancy outcome following intracytoplasmic sperm injection. *J Assist Reprod Genet* **37**, 829-840 (2020).

80. Khan, M. R. *et al.* Genome sequencing of Pakistani families with male infertility identifies deleterious genotypes in SPAG6, CCDC9, TKTL1, TUBA3C, and M1AP. *Andrology* https://doi.org/10.1111/andr.13570 (2023).

81. Tang, X. *et al.* A novel variant in CFAP69 causes asthenoteratozoospermia with treatable ART outcomes and a literature review. *J Assist Reprod Genet* **40**, 2175-2184 (2023).

82. He, X. *et al.* Novel homozygous CFAP69 mutations in humans and mice cause severe asthenoteratospermia with multiple morphological abnormalities of the sperm flagella. *J Med Genet* **56**, 96-103 (2019).

83. Dong, F. N. *et al.* Absence of CFAP69 causes male infertility due to multiple morphological abnormalities of the flagella in human and mouse. *Am J Hum Genet* **102**, 636-648 (2018).

84. Liu, C. *et al.* Homozygous mutations in SPEF2 induce multiple morphological abnormalities of the sperm flagella and male infertility. *J Med Genet* **57**, 31-37 (2020).

85. Aprea, I. *et al.* Pathogenic gene variants in CCDC39, CCDC40, RSPH1, RSPH9, HYDIN, and SPEF2 cause defects of sperm flagella composition and male infertility. *Front Genet* **14**, 1117821 (2023).

86. Mori, M. *et al.* Novel SPEF2 variant in a Japanese patient with primary ciliary dyskinesia: A case report and literature review. *J Clin Med* **12**, 317 (2022).

87. Lu, W. *et al.* Novel SPEF2 variants cause male infertility and likely primary ciliary dyskinesia. *J Assist Reprod Genet* **41**, 1485-1498 (2024).

88. Cindrić, S. *et al.* SPEF2- and HYDIN-mutant cilia lack the central pair-associated protein SPEF2, aiding primary ciliary dyskinesia diagnostics. *Am J Respir Cell Mol Biol* **62**, 382-396 (2020).
89. Sha, Y. *et al.* Biallelic mutations in Sperm flagellum 2 cause human multiple morphological abnormalities of the sperm flagella (MMAF) phenotype. *Clin Genet* **96**, 385-393 (2019).
90. Liu, W. *et al.* Loss-of-function mutations in SPEF2 cause multiple morphological abnormalities of the sperm flagella (MMAF). *J Med Genet* **56**, 678-684 (2019).
91. Zhang, Z. *et al.* A heterozygous mutation disrupting the SPAG16 gene results in biochemical instability of central apparatus components of the human sperm axoneme. *Biol Reprod* **77**, 864-871 (2007).
92. Meng, Z. *et al.* Identification of bi-allelic KIF9 loss-of-function variants contributing to asthenospermia and male infertility in two Chinese families. *Front Endocrinol (Lausanne)* **13**, 1091107 (2022).
93. Chrystal, P. W. *et al.* The inner junction protein CFAP20 functions in motile and non-motile cilia and is critical for vision. *Nat Commun* **13**, 6595 (2022).
94. Wang, W. *et al.* Biallelic mutations in CFAP65 lead to severe asthenoteratospermia due to acrosome hypoplasia and flagellum malformations. *J Med Genet* **56**, 750-757 (2019).
95. Li, W. *et al.* Biallelic mutations in CFAP65 cause male infertility with multiple morphological abnormalities of the sperm flagella in humans and mice. *J Med Genet* **57**, 89-95 (2020).
96. Zhang, X. *et al.* A novel homozygous CFAP65 mutation in humans causes male infertility with multiple morphological abnormalities of the sperm flagella. *Clin Genet* **96**, 541-548 (2019).
97. Beurois, J. *et al.* CFAP70 mutations lead to male infertility due to severe astheno-teratozoospermia. A case report. *Hum Reprod* **34**, 2071-2079 (2019).
98. Jin, H. J. *et al.* CFAP70 is a solid and valuable target for the genetic diagnosis of oligo-astheno-teratozoospermia in infertile men. *EBioMedicine* **93**, 104675 (2023).
99. Olbrich, H. *et al.* Recessive HYDIN mutations cause primary ciliary dyskinesia without randomization of left-right body asymmetry. *Am J Hum Genet* **91**, 672-684 (2012).
100. Benjamin, A. T. *et al.* A Novel Homozygous Nonsense HYDIN Gene Mutation p.(Arg951*) in Primary Ciliary Dyskinesia. *Indian J Pediatr* **86**, 664-665 (2019).
101. Fleming, A. *et al.* Combined approaches, including long-read sequencing, address the diagnostic challenge of HYDIN in primary ciliary dyskinesia. *Eur J Hum Genet* **32**, 1074-1085 (2024).
