# Supplementary material for: In situ structure of the mouse sperm central apparatus reveals mechanistic insights into asthenozoospermia
Source: Cell Res. 2025 Jun 5;35(8):551–67. doi: 10.1038/s41422-025-01135-2 (PMC12297659; doi:10.1038/s41422-025-01135-2)
Supplement: Supplementary file 39 — Supplementary information, Table S8 [file 41422_2025_1135_MOESM39_ESM.pdf]

Supplementary information, Table S8. The primers for PCR and qPCR reactions.

Gene	Region	Primer	Sequence (5' to 3')	Product size
CFAP47 (PCR)	Exon 26	Forward	AAGCCAATGACTGGCAGGTA	257 bp
		Reverse	TGTCATCACAGAAAAGAAGATTGG	
	Exon 27	Forward	GCAACAGCAGAAAACTGCAT	219 bp
		Reverse	CTCAATGACAAGCACAGCTTC	
	Exon 28	Forward	GATGGTGTTTTGCCTCCCTA	292 bp
		Reverse	TGACCCTTGAAGACAACTCTCC	
	Exon 61	Forward	AGTTGGAATCCTGTGTAGCTTT	300 bp
		Reverse	CAACCATTTAGACTACATTCTTCTCA	
	Exon 62	Forward	TTCACGTAGTGTCATTTAAAATCTCC	265 bp
		Reverse	AAATTTCATTCCAAAGTTCAGCA	
	Exon 63	Forward	GGCCTATTGCAGCAGGATT	227 bp
		Reverse	GGTTTAAATCCTACAGTGATGAGAGTT	
CFAP47 (qPCR)	Exon 28	Forward	GATGGTGTTTTGCCTCCCTA	108 bp
		Reverse	GTTTCCCTTTGCAGGTTCAG	
	Exon 48	Forward	ATGGTAGGGAGCACATCTTTG	100 bp
		Reverse	TTTTGTGTCACATTCCCCACT	
	Exon 62	Forward	GCGTAACAGAAGGGATCTGG	125 bp
		Reverse	TTTCAGCCTAAGTTCAAAAACAGA	

The transcript used in this study was NM_001304548.2.
