# Supplementary material for: In situ structure of the mouse sperm central apparatus reveals mechanistic insights into asthenozoospermia
Source: Cell Res. 2025 Jun 5;35(8):551–67. doi: 10.1038/s41422-025-01135-2 (PMC12297659; doi:10.1038/s41422-025-01135-2)
Supplement: Supplementary file 41 — Supplementary information, Table S10 [file 41422_2025_1135_MOESM41_ESM.pdf]

Supplementary information, Table S10. Color scheme of CA component proteins in our structure.

Primary Location*	Mouse Sperm	Color name (ChimeraX)
Microtubule wall	Tubulin α3	lemon chiffon
	Tubulin β-4B	khaki
C1-MOSP	CFAP46	peru
	CFAP54	teal
	CFAP74	light coral
	CFAP99	rosy brown
	CFAP221	pale turquoise
	LRRC72	light steel blue
	CCDC180	medium sea green
	DLEC1	dark sea green
	SPAG6	coral
	GRK3	pale green
	PPP1CC	olive drab
	ANKMY1	light goldenrod yellow
	LRRC43	cadet blue
	Unknown1	fire brick
	Unknown2	medium violet red
	Unknown3	orange red
C1a arm	CFAP119	chartreuse
	Calmodulin	yellow green
	MORN2	gold
	DPY30	dark salmon
	SPAG17	dark goldenrod
	SPATA17	thistle
C1b arm	CFAP69	wheat
	SPEF2	pink
	LRGUK	cyan
	GOT1L1	light green
	LRRD1	burly wood
C2-MOSP	SPAG16	dark olive green
	KIF9	light blue
	FAM228B	dodger blue
	CFAP20	steel blue
	MYCBPAP	blue
	SPATA4	light sea green
C2a arm	CFAP65	purple
	CFAP70	pale violet red
C2b arm	HYDIN	royal blue
	MAP1S	Rebecca purple
Bridge	CFAP47	deep pink
	GMCL1(BTBD16)	dark sea green
MIP	SPACA9	orange
	Unknown4	dark blue
	Unknown5	medium slate blue
	Unknown6	dark slate gray

* For CA proteins present in multiple locations, one of them is designated as its primary location.
