# Supplementary material for: In situ structure of the mouse sperm central apparatus reveals mechanistic insights into asthenozoospermia
Source: Cell Res. 2025 Jun 5;35(8):551–67. doi: 10.1038/s41422-025-01135-2 (PMC12297659; doi:10.1038/s41422-025-01135-2)
Supplement: Supplementary file 43 — Supplementary information, Video legend [file 41422_2025_1135_MOESM43_ESM.pdf]

Supplementary information, Video S1.

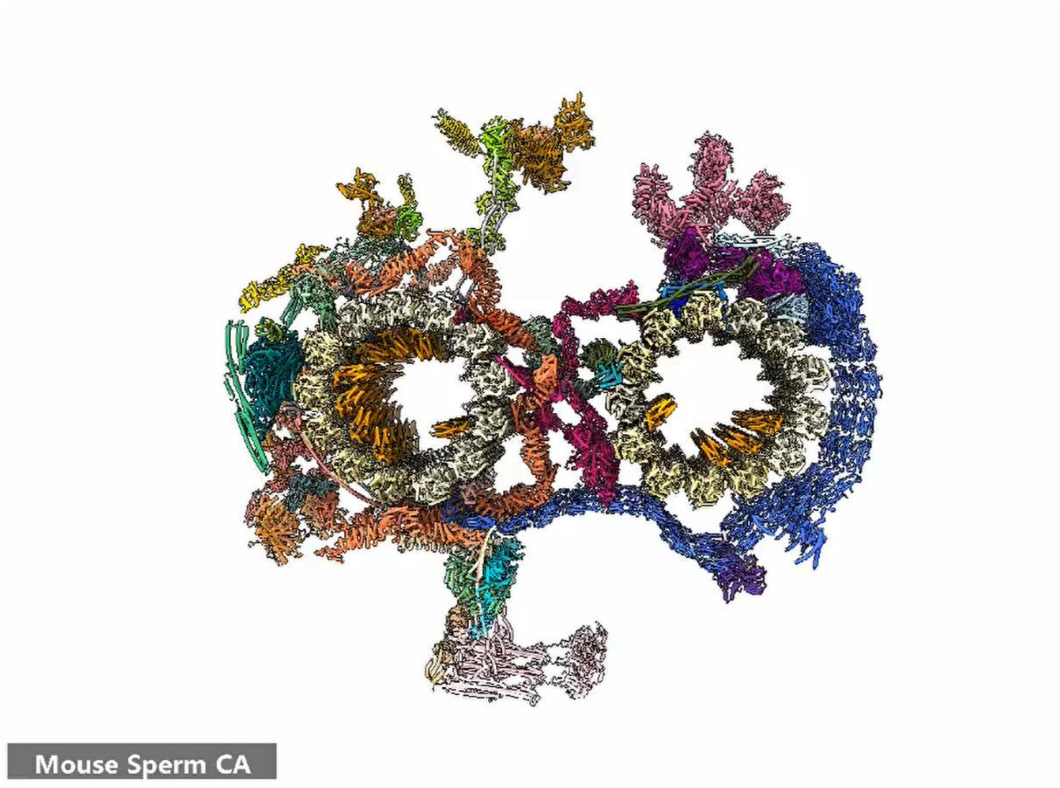

Video S1. Structural organization of mouse sperm CA.

Supplementary information, Video S2.

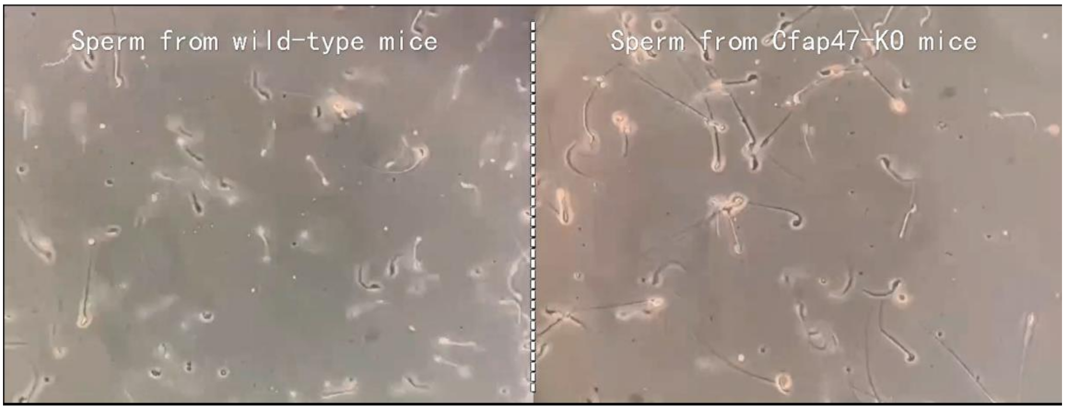

Video S2. The motility of sperm from wild-type and *Cfap47*-KO mice.

Supplementary information, Video S3.

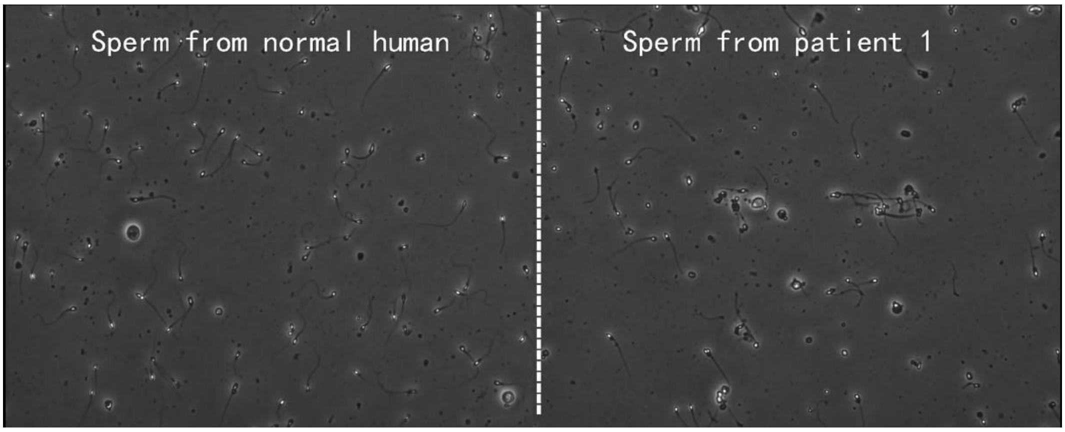

Video S3. The motility of sperm from normal human and patient 1.
